# Machine Learning in Computational Surface Science and Catalysis: Case Studies on Water and Metal–Oxide Interfaces

**DOI:** 10.3389/fchem.2020.601029

**Published:** 2020-11-30

**Authors:** Xiaoke Li, Wolfgang Paier, Joachim Paier

**Affiliations:** ^1^Institut für Chemie, Humboldt-Universität zu Berlin, Berlin, Germany; ^2^Fraunhofer Institute for Telecommunications, Heinrich Hertz Institute HHI, Berlin, Germany

**Keywords:** MgO, magnetite, density functional theory, machine learning, force fields

## Abstract

The goal of many computational physicists and chemists is the ability to bridge the gap between atomistic length scales of about a few multiples of an Ångström (Å), i. e., 10^−10^ m, and meso- or macroscopic length scales by virtue of simulations. The same applies to timescales. Machine learning techniques appear to bring this goal into reach. This work applies the recently published on-the-fly machine-learned force field techniques using a variant of the Gaussian approximation potentials combined with Bayesian regression and molecular dynamics as efficiently implemented in the Vienna *ab initio* simulation package, VASP. The generation of these force fields follows active-learning schemes. We apply these force fields to simple oxides such as MgO and more complex reducible oxides such as iron oxide, examine their generalizability, and further increase complexity by studying water adsorption on these metal oxide surfaces. We successfully examined surface properties of pristine and reconstructed MgO and Fe_3_O_4_ surfaces. However, the accurate description of water–oxide interfaces by machine-learned force fields, especially for iron oxides, remains a field offering plenty of research opportunities.

## Introduction

Machine learning (ML) is currently attracting the interest of a broad community. Searching the keywords *machine learning* and *chemistry* combined by the Boolean AND yields about 4,300 hits solely for the year 2019. This is because the potential of ML or “self-learning” algorithms has been widely recognized, while substantially affecting not only the chemistry research but also our everyday lives. For example, these algorithms made developments such as state-of-the-art voice and face recognition possible (Galbally et al., 2014; Meng et al., 2015). Music, smartphones, and even cars benefit from ML. With respect to applications in the realm of computational chemistry, especially cheminformatics and molecular modeling, ML techniques like multivariate regression and artificial neural networks (ANNs) have been used since the early days of computational disciplines. The intensity of research in ML-related computational chemistry and physics has gained tremendous impetus just in recent years. This is largely due to the steady increase in efficiency of hardware, as for instance, fast graphical processing units, tensor-processing units (Jouppi et al., 2018), and other application-specific integrated circuits speeding up computations to an unprecedented extent of meanwhile hundreds of PFLOPs (peta = 10^15^; FLOP = floating-point operation) per second. This vast increase as well as the development of special matrix execution units tailor-made for ML applications such as the training of ANNs nourishes the hope of being able to tackle complex “real-world problems.”

Turning to a chemical application, the development or even the mere optimization of catalysts (i.e., materials that enable chemical reactions under more benign conditions compared to running without catalyst) represents an extraordinarily complex physicochemical problem. Many excellent reviews on the importance of so-called structure–activity relationships (SARs) have been published. SAR means that a catalyst's functionality, say a structural element or a functional group, is causally related to its reactivity (Salciccioli et al., 2011; Sauer and Freund, 2015; Schlögl, 2015). To find out which electronic, atomic, or molecular structure elements and—more generally—functionalities relate to specific physicochemical properties of a compound or material (and *vice versa*) represents one of the central interests of chemists and physicists. Hence, given the importance of structure or “topological connectivity,” it does not come as a surprise that graph theoretical approaches and clustering analyses represent essential elements in the mathematical toolbox of chemists (Bartel, 1996). These methods have been widely employed in molecular modeling studies for drug discovery, for instance (Leach, 2001). In addition, multivariate regression such as partial least squares (Hopfinger et al., 1997; Kubinyi, 1997) and ANN (Zupan and Gasteiger, 1991; Gasteiger and Zupan, 1993) have become standard ML tools in the early 1990s of the past century. They are widely applied in quantitative SARs (QSAR) studies.

These days see an overwhelming amount of ML-related research in materials science (Schleder et al., 2019), computational chemistry (Jinnouchi and Asahi, 2017; Cova and Pais, 2019; Janet et al., 2019; Kulik, 2020), and physics (Jinnouchi et al., 2019b, 2020a), including electronic structure theory (Brockherde et al., 2017). Essentially, this research can be divided into two branches: first, data mining or big data applications that involve the fast screening or filtering of gigantic structure-property databases (Ghiringhelli et al., 2015; Draxl and Scheffler, 2018), and second, research with focus on multiscale atomistic simulations that tackle the problem of bridging length and timescales. This can be accomplished by developing ML density functional theory (DFT) or entirely bypassing the need to solve the Kohn–Sham (KS) Schrödinger equation, by developing force fields (FFs) using interatomic potentials such as the Gaussian approximation potentials (GAPs; Bartók et al., 2010) or the many-body potentials relying on ANN (Behler et al., 2008).

The present work systematically explores capabilities of so-called “on-the-fly” machine-learned FFs (MLFFs) (Jinnouchi et al., 2019b) using a variant of the GAP approach together with molecular dynamics (MD) runs, as recently implemented in the Vienna *ab initio* simulation package (VASP). We apply these FFs to relevant problems in surface and interface science valuable for catalysis research. After recapitulating essentials of Bayesian regression, on which MLFF is based, we assess its performances on simple MgO clusters, surfaces, and the adsorption of water on the MgO(001) surface. Moreover, we examine by virtue of MLFF and DFT more complex FeO_x_ clusters, the stability of ideal surface terminations of Fe_3_O_4_(111), and the adsorption of water molecules on that surface. The present study also includes calculated stabilities of some steps or line defects on Fe_3_O_4_(111), which are, to the best of our knowledge, computationally out of reach, when using established first-principles methods such as DFT (due to system size). It is important to clarify that the main motivation of the present work lies not in competing with highly accurate results from an electronic structure method point of view, but we want to address practical aspects of MLFFs, their *transferability* or *generalizability*, meaning applicability beyond training sets instead.

## Models and Methods

The MLFF approach of Jinnouchi et al. (2019b) is a typical example of an active learning (AL) method. The key concept behind such algorithms is a dynamically generated training dataset, where the learning algorithm can query a so-called teacher, which provides a target-signal **y** for any given input ***X***. This incremental generation of training data is especially useful in applications where the creation/computation of an extensive training dataset is prohibitively costly or simply not feasible.

The bottleneck in the construction of an MLFF is the need for a sufficiently dense reference dataset, which is constructed from costly in-time quantum mechanics–based (QM) calculations. Importantly, there are no *a priori* rules to decide how large these datasets must be in order to span the relevant part of the feature space.

The active-learning scheme solves these problems, by offering an unbiased and systematic selection of relevant training structures during simulation. Only structures that have been identified as relevant serve as input for QM calculations. Therefrom obtained results enter the reference dataset, which in turn serves for updating the MLFF. This approach avoids many unnecessary QM calculations, which yields enormous savings in computer time.

For a comprehensive description of the on-the-fly MLFF generation during MD simulations implemented in VASP and employed in the present work, we refer to Jinnouchi et al. (2019a) and Jinnouchi et al. (2019b). AL is amply discussed in, e.g., Artrith and Behler (2012), Miwa and Ohno (2017), Jacobsen et al. (2018), Zhang et al. (2019), and (Jinnouchi et al., 2020c).

### FF Generation

The beauty of FFs lies in its separable ansatz (1) for the total (potential) energy *U* as well as for gradients of *U*, etc.:

(1) $$\begin{array}{l} U=\sum_{i=1}^{N_{atoms}}U_{i}, \end{array}$$

with *U*_*i*_ determined by the local environment of atom *i* and interpretable as an *effective* atomic contribution. This requires a representation of *U* in terms of atomic structure information. The approach (2) used in the present work employs the so-called GAP ansatz:

(2) $$\begin{array}{l} U_{i}=\sum_{i_{B}=1}^{N_{B}}w_{i_{B}}\cdot K\left(X_{i},X_{i_{B}}\right). \end{array}$$

This means that a set of *N*_*B*_ local reference structures is chosen and expressed with descriptors ***X***_*i*_*B*__. The coefficients *w*_*i*_*B*__ are determined via fitting, and the kernel function *K* represents a similarity measure between a certain local configuration *i* and a reference configuration *i*_*B*_. For the descriptors and the kernel function, a variant of the so-called smooth overlap of atomic positions has been adopted (Bartók et al., 2013; Jinnouchi et al., 2019a). The big advantage of this method is the capability of fitting energies and gradients together with their uncertainties, a key component in AL. In Jinnouchi et al. (2019a), also the symmetrically inequivalent components of the stress tensor have been used, but this has not been done in the present work.

The ansatz expressed in (1) allows representing energies and gradients for all training structures in a compact matrix-vector form,

(3) $$\begin{array}{l} \text{y}=\phi w, \end{array}$$

with a column vector **w** containing the *N*_*i*_*B*__ coefficients *w*_*i*_*B*__, and ***ϕ*** is a matrix containing *K*(***X***_*i*_, ***X***_*i*_*B*__) and its derivatives with respect to atomic coordinates. Importantly, the Bayesian regression assumes the existence of an optimal set of model coefficients **w** that allows inferring energies and gradients based on the descriptor matrix ***ϕ***. Furthermore, it models the relationship between **y** and ***ϕ*** as a Gaussian Process. Thus, all errors and noise are subject to a multidimensional Gaussian or normal distribution.

While both GAP and MLFF employ the Gaussian process for fitting energies, the underlying equations to solve for the model coefficients are different. The GAP method uses kernel ridge regression (RR), whereas MLFF implements a Bayesian regression approach. They are equivalent in the limit of zero regularization, i.e., abstaining from any numerical techniques to avoid overfitting. The basics of Bayesian regression are briefly outlined in the subsequent section.

### Bayesian Regression of Model Coefficients

To estimate FF parameters based on a database of precomputed reference data, we employ a linear regression model (3). Instead of ordinary least squares (OLS) or RR, we use Bayesian linear regression.

The key advantage of the Bayesian approach over OLS or RR lies in the fact that it provides a regularized least-squares solution for the model coefficient **w** as well as a measure of uncertainty for the prediction. The measurement of uncertainty is an important feature, which is used in the employed VASP implementation and allows skipping costly DFT calculations for new atomic configurations that are close to reference data points where accurate DFT results are already available.

The Bayesian regression framework models noise in the training data, as well as the uncertainty in the prediction ***y*** with Gaussian distributions. Model coefficients are defined by the Gaussian distribution $N\left(\hat{w},\Sigma \right)$, with $\hat{w}$ (4) being the optimal model weights given by least-squares solution and the covariance matrix **Σ** (5) (Rasmussen and Williams, 2006) and a regularization weight γ.

(4) $$\begin{array}{l} \hat{w}=\frac{1}{\sigma^{2}}\Sigma \phi^{T}y \end{array}$$

(5) $$\begin{array}{l} \Sigma =\sigma^{2}{\left(\phi^{T}\phi +\;\gamma I\right)}^{-1} \end{array}$$

For a new configuration represented by ϕ^*^ and the corresponding prediction y^*^, we can estimate the uncertainty $P\left(y^{*}|\phi^{*},D\right)$ given the dataset ***D*****=**{***X***_***i***_**, *****y***} by integrating over ***w*** (Rasmussen and Williams, 2006). Dataset ***D*** consists of all training structures ***X***_*i*_ as well as the corresponding energies and gradients ***y***. This yields the following equations

(6) $$\begin{array}{l} P\left(y^{*}|\phi^{*},D\right)=\int_{w}P\left(y^{*}|\phi^{*},w\right)P\left(w|D\right)dw\\ \;=\int_{w}N\left(y^{*}|\phi^{*}w,\sigma^{2}\right)N\left(w|\hat{w},\Sigma \right)dw\\ \;=N\left(y^{*}|\phi^{*}\hat{w},\sigma^{2}+\phi^{*}\Sigma \phi^{*T}\right). \end{array}$$

Hence, the mean ***μ***_pred_ and the variance **Σ**_pred_ of the posterior predictive distribution can be written as follows:

(7) $$\begin{array}{l} \mu_{\text{pred}}=\phi^{*}\hat{w} \end{array}$$

(8) $$\begin{array}{l} \Sigma_{\text{pred}}=\;{\sigma^{2}+\phi }^{*}\Sigma \phi^{*T}. \end{array}$$

Following the proposed method in Jinnouchi et al. (2019a), parameters σ^2^ and γ are optimized via the evidence approximation. This maximizes the marginal likelihood function, which corresponds to the probability that the regression model provides the reference data ***y***. For more details, we refer the interested reader to Jinnouchi et al. (2019a), as well as (Gull and Skilling, 1989; MacKay, 1992; Jinnouchi and Asahi, 2017).

### Challenges for MLFF

The separable ansatz (1) offers tremendous computational savings, but on the other hand sacrifices accuracy in two respects. The first shortcoming in MLFF is its inherent “shortsightedness” due to the decomposition of the potential energy into local contributions. Especially in ionic materials where long-range electrostatic interactions play a role, this may incur problems. Also, long-range van der Waals type of interactions cannot be exactly described by (1). The only solution to that problem is avoiding (1) and employing representations of the energy with descriptors capable to describe long-range interactions. We do not go into details here but refer to current developments in this respect (Chmiela et al., 2017; Grisafi and Ceriotti, 2019; Gkeka et al., 2020).

The second aspect involves the representation of the atomic structure and therefrom incurred short-range many-body interactions. As discussed in Jinnouchi et al. (2019a) and Jinnouchi et al. (2020b), MLFF represents short-range many-body interactions as a nonlinear function of two- and three-body descriptors. As a matter of fact, two completely different structures can lead to an identical set of two- and three-body descriptors, in turn yielding (by construction) the identical energy (Glielmo et al., 2018; Pozdnyakov et al., 2020). Descriptors that uniquely represent many-body configurations exist (Shapeev, 2016; Glielmo et al., 2018; Oord et al., 2020); however, they come at an increased computational workload and cost. In addition, the question whether higher-order many-body descriptors substantially improve on accuracy compared with two- and three-body descriptors has not been answered yet.

## Technical Details

### Electronic Structure Calculations and MLFF Generation

All calculations discussed throughout this work were carried out using a development version of the Vienna *ab initio* simulation package VASP.6 (Kresse and Furthmüller, 1996a,b; Kresse and Joubert, 1999). To solve the KS Schrödinger equations, the projector augmented wave (PAW) ansatz (Blöchl, 1994; Marsman and Kresse, 2006) to describe the interaction between valence and core electrons was used. Thus, all calculations use the PAW pseudopotentials released together with VASP version 5.4 and plane waves as a basis set. For the plane wave expansion of Bloch waves, the minimal (default) kinetic energy cutoff was employed except for MD runs on the H_2_O/Fe_3_O_4_(111) surfaces to train the MLFF. These calculations use 600 eV. According to our experience, increasing the cutoff enhances convergence of the self-consistent field (SCF) cycles for magnetite calculations. The PAW dataset for Fe includes the [Ar] 3p core orbitals in the valence space. Hence, in total, 14 valence electrons are treated in the SCF optimization. The pseudopotentials for Mg and O use two and six valence electrons, respectively. Regarding the KS-DFT approximation to exchange and correlation (xc) effects, the Perdew, Burke, and Ernzerhof (PBE) (Perdew et al., 1996) generalized-gradient approximation to the xc energy and potential is employed in the present work. Calculations on MgO were carried out non–spin-polarized, whereas calculations on Fe_3_O_4_ employ spin-polarized PBE [see also (Li and Paier, 2016)].

With respect to the training or learning of MLFFs, we follow the description provided online via the VASP-wiki (VASP, 2020). According to these descriptions, *NVT* ensemble MD simulations for MgO clusters as well as bulk structures were carried out at 2,000 K using a Nosé-Hoover thermostat (Nosé, 1984; Hoover, 1985). MD runs to train the MLFF for Fe_3_O_4_ were carried out at a temperature of 2,500 K. As described below in detail, MD runs to train the MLFF involving H_2_O adsorption use lower temperatures. In principle, also the *NpT* ensemble together with a Langevin thermostat can be used for bulk phases instead. However, because it is important to avoid Pulay stress in variable cell volume calculations, a substantially increased plane wave cutoff and denser FFT grids must be employed in *NpT* runs. As MD runs involving at least 10,000 steps of 1–3 fs per time step are required to train the MLFFs, *NVT* ensemble calculations are preferred because of lower computational workload. We restarted these on-the-fly training simulations according to needs; i.e., for instance, until uncertainties in the prediction were sufficiently small (see below). Importantly, this work relies on MD runs to generate training (data) sets using clusters as well as periodic structures such as surfaces and adsorbates (H_2_O) on surfaces.

Simulations on H_2_O adsorbed on MgO(001) or Fe_3_O_4_(111) surfaces use 150 K, deuterium instead of H, and a significantly smaller time step of 0.2 fs. Regarding integrations over *k* points of Brillouin zones, a Monkhorst-Pack (2 × 4) *k* mesh for the *p*(3 × 2) – MgO(001) and a (5 × 5) one for the primitive Fe_3_O_4_(111) surfaces were used. PBE atomic reference energies for training runs were obtained with spin-polarized calculations (except for the Mg atom) and a 12 × 13 × 14 Ångström (Å)^3^ box and used the tag LASPH = TRUE to include aspherical contributions (*l* > 0) for xc gradient corrections within the PAW sphere around the ionic cores. Furthermore, to enable efficient calculations, an energy break criterion in electronic relaxations (SCF) of 10^−4^ eV was employed. Open-shell (spin-polarized) FeO_x_-related calculations use an energy break criterion of 10^−5^ eV. Input structures for these MLFF-training MD runs are described in detail in the following section.

The FFs were sufficiently long trained, such that the Bayesian errors in the total energy/atom, the force, and the so-called spilling factor (Jinnouchi et al., 2019a) printed at the end of a run (ML_LOGFILE) were smaller than defaulted thresholds. Values for errors in energies/atom and forces typically amount to 0.03 (MgO) to 0.08 (FeO_x_) eV/atom and 0.3 to 0.4 eV/Å, respectively. In addition, we carefully checked that the error in the radial expansion was well below the (empirically determined) value of ±0.02 (Szlachta et al., 2014).

### Structure Models

**Figure 1:**
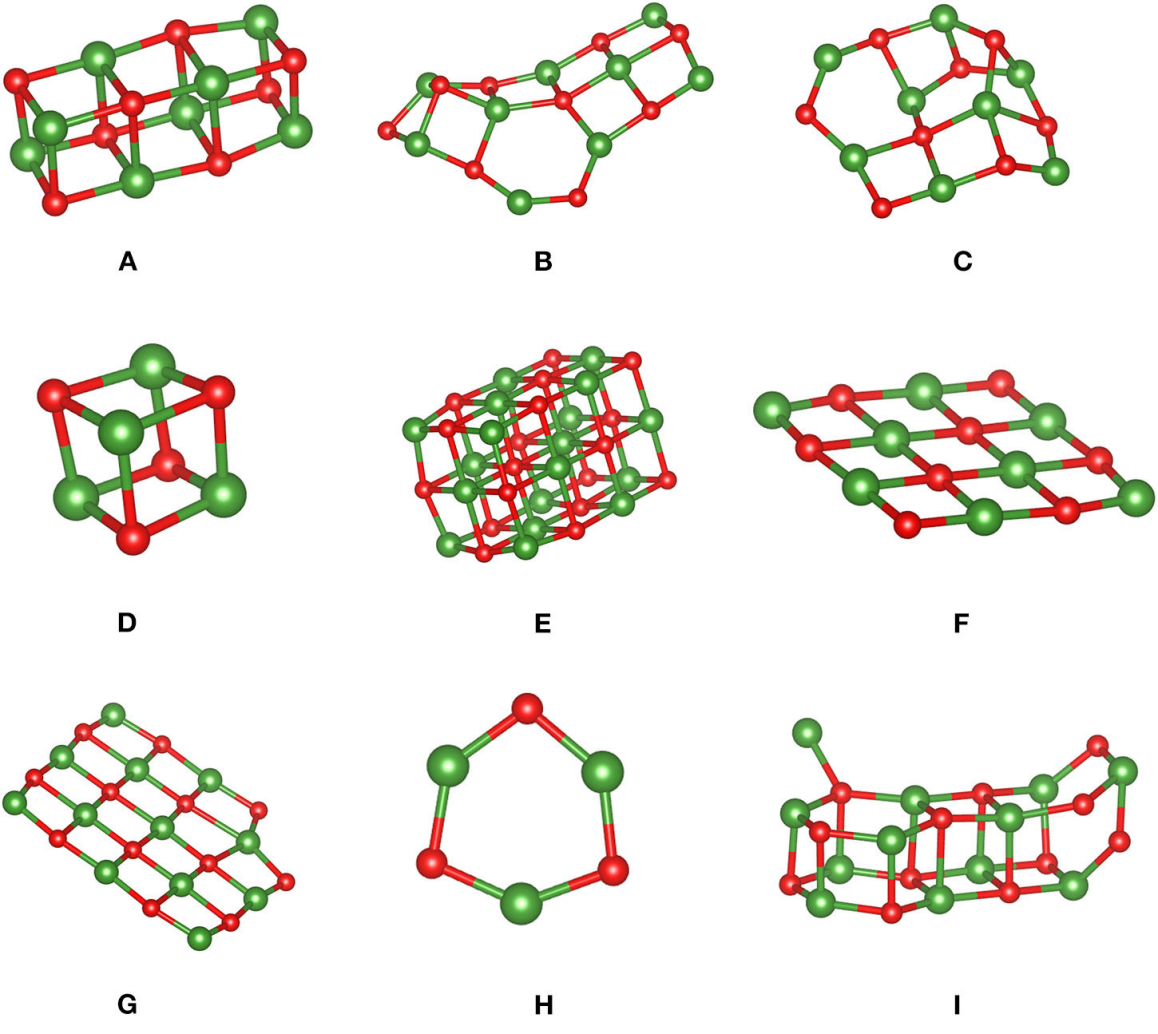
MgO clusters **(A–I)** optimized using PBE and used to assess MLFFs. Color code: Mg is green, and oxygen is red.

The MLFF for MgO nonperiodic clusters and periodic surfaces (without H_2_O) was trained using cluster **A** with composition {Mg_9_O_9_} as shown in Figure 1 (see Results and Discussion). Simulations on that cluster used a cell with a dimension 10 × 10 × 30 Å^3^ (Supplementary Material). To improve on that MLFF, we restarted the training using cluster **I** with composition {Mg_12_O_12_}. Alternatively, we trained a second MLFF for MgO using bulk structures. For this purpose, the cubic, the wurtzite, and the zincblende structures have been employed. Prior to the (restarted) MD training runs, these structures, i.e., lattice parameters, angles, and ionic positions, were optimized using the PBE functional until the maximal atomic force was <0.01 eV/Å. Note that MLFFs of H_2_O adsorbed on the MgO(001) surfaces were obtained starting from the *p*(3 × 2) overlayer structure as published in Wlodarczyk et al. (2011).

For FeO_x_ cluster and surface calculations without water, we do not use clusters for the on-the-fly MLFF generation, because convergence of these open-shell magnetic structures is involved. Instead, we employed a “minimal” five atomic layers thick and symmetric slab model of the Fe_3_O_4_(111) surface (Supplementary Material). We used the previously computed PBE+U lattice parameters, because the PBE and PBE+U parameters differ by 1.4% only (see Kiejna et al., 2012; Yu et al., 2012). This difference is negligibly small for the purpose of the present work to show general performances of MLFFs. The total magnetic moment of the slab was constrained to zero. We initialized local magnetic moments of tetrahedrally and octahedrally coordinated Fe ions to −3.5 and +3.5 μ_B_ corresponding to an antiferromagnetic order as examined many times (Kiejna et al., 2012; Noh et al., 2014; Santos-Carballal et al., 2014), although magnetism becomes certainly less relevant at higher temperatures beyond T_Curie_ reached during the training MD simulations.

MLFFs used in H_2_O adsorption on Fe_3_O_4_(111) – (1 × 1) surfaces were created in a stepwise manner. We generated MLFFs for one, two, three, and four H_2_O molecules per primitive surface unit cell of a 12-atomic-layer-thick asymmetric slab model for an Fe_tet1_ terminated surface, the same as used in Li and Paier (2016). MD runs comprising ca. 10,000 steps (total simulation time: 2 ps) on overlayers containing N_H2O_ water molecules were carried out for each of these coverages but were restarted from the optimized structure with (N_H2O_ – 1) molecules. This means that we started the (2 × H_2_O)/Fe_3_O_4_(111) – (1 × 1) run using the reference structure pool (ML_ABNCAR) obtained from the H_2_O/Fe_3_O_4_(111) – (1 × 1) run, the (3 × H_2_O)/Fe_3_O_4_(111) – (1 × 1) run was started using the (2 × H_2_O)/Fe_3_O_4_(111) – (1 × 1) structure pool, and so on up to a loading of four H_2_O molecules per Fe_3_O_4_(111) – (1 × 1) surface unit cell. Importantly, to avoid desorption events, the temperature was set to 150 K. Adding a correction for missing van der Waals dispersion effects helped to avoid desorption of individual D_2_O molecules during the training of the FFs. We used the approach after Grimme in its D2 variant (Grimme, 2006; Bučko et al., 2010); default van der Waals R_0_ and C_6_ parameters for Mg, Fe, O, and H (D) atoms; and a global scaling factor, s_6_ = 0.75, fitted for the PBE functional (Grimme, 2006).

## Results and Discussion

### MLFF for MgO Clusters and Surfaces

Figure 1 shows structures of the MgO clusters used to assess performances of the trained MLFFs. Note that structure **A** was used to generate an MLFF specifically for clusters (Table 1, FF_cluster_), and it was retrained using structure **I** with a “dangling Mg ion.” A second, independently learned FF was trained on MgO bulk phases including cubic, wurtzite, and zincblende structures (Table 1, FF_bulk_). These structures were designed to span a relatively broad spectrum of motifs comprising rods (structures **A** and **I**), cubes (structures **D** and **E**), sheets with (structures **B** and **C**), and without puckering (structures **F** and **G**), as well as cycles or rings (structure **H**). They were optimized using the PBE functional and hence represent local energy minima. Eventually, these clusters were used as an input for (re-)optimizations of atomic positions using FF_cluster_ and FF_bulk_.

**Table 1:** Relative energies (ΔE in eV/MgO) obtained using PBE—as the reference—and two distinctly generated force fields.

	**A**	**B**	**C**	**D**	**E**	**F**	**G**	**H**	**I**
	**{Mg_**8**_O_**8**_}**	**{Mg_**9**_O_**9**_}**	**{Mg_**8**_O_**8**_}**	**{Mg_**4**_O_**4**_}**	**{Mg_**18**_O_**18**_}**	**{Mg_**8**_O_**8**_}**	**{Mg_**12**_O_**12**_}**	**{Mg_**3**_O_**3**_}**	**{Mg_**12**_O_**12**_}**
ΔE^PBE^	0.64	1.10	1.06	1.43	0.00	0.89	0.84	1.98	0.84
Order	**2**	**7**	**6**	**8**	**1**	**5**	**4**	**9**	**3**
FF_bulk_^a^									
ΔE	0.33	0.60	0.59	0.28	0.13	0.46	0.46	0.00	—^b^
Order	**4**	**8**	**7**	**3**	**2**	**6**	**5**	**1**	—
MSE	−0.64								—
MUE	0.67								—
FF_cluster_^a^									
ΔE	0.40	0.94	0.91	0.97	0.00	0.99	0.86	1.68	0.46
Order	**2**	**6**	**5**	**7**	**1**	**8**	**4**	**9**	**3**
MSE	−0.17								
MUE	0.20								

*FF_bulk_ is trained on bulk structures (see Technical Details), and FF_cluster_ is trained using clusters. The row “order” (bold values) refers to the order of clusters by relative energy ΔE, with 1 as the most stable and 9 as the least stable cluster. MSE, mean signed error; MUE, mean unsigned error*.

a *Structure optimized*.

b *Diverged due to dangling Mg ion (see text)*.

Not surprisingly, the MLFF trained on bulk structures (FF_bulk_) performs worse compared with results obtained using the cluster FF (FF_cluster_). Overall bonding characteristics such as bond distances and angles strongly deviate from PBE reference values. The magnitude of these discrepancies depends on the individual cluster type. Most of the MgO distances obtained using FF_bulk_ are systematically too short. This underestimation can vary between 1.5 and 3%, as is the case for structure **A**, but it can amount up to ca. 30% as in cluster **C** featuring a more complicated structure. Note that the “dangling Mg ion” cannot be described properly as it dissociates away during the structure optimization employing FF_bulk_. Also, the Mg–Mg distance in cluster **H** is grossly underestimated by 56% (it shrinks from its PBE result of 2.908 to 1.263 Å such that a “two-ring motif” featuring an inner Mg ring with bridging O ions is formed. Consequently, the energy ordering based on FF_bulk_ is incorrect, which is also reflected by a too-narrow energy window. This means that the difference between least stable and most stable structure amounts to 0.60 eV/MgO unit instead of the 1.98 eV/MgO obtained using DFT-PBE. Mean signed (MSE) and unsigned (MUE) or absolute errors are of the same magnitude, indicating the systematic underestimation of cluster stabilities obtained with FF_bulk_.

The performance of the FF_cluster_ is substantially better. Structural differences in bond lengths and angles are minute when compared to PBE results. The aforementioned systematic underestimation of Mg-O bond distances is alleviated to within a 1% range. Structure **I** is maintained during optimization using FF_cluster_, and the dangling Mg ion is still bound to the MgO rod, although the angle to the plane determined by the dangling Mg ion and the rod's surface is too small by about 20°. Also, cluster **B** is too pyramidalized (see O-Mg_3_ pyramid on the left-hand side of the structure), indicating underestimated bond angles. Regarding the ordering by relative energy per MgO unit, the order predicted by PBE reference energies is almost identically reproduced except for cluster types being close in energy using PBE. Out of this structure pool, FF_cluster_ correctly identifies the large Mg_18_O_18_ cube (structure **E**) as the most stable, and cubic structure and the (MgO)_3_ ring (structure **H**) as the least stable structure. Their relative energies span a window of 1.68 eV/MgO. This agrees reasonably well with the corresponding PBE result of 1.98 eV/MgO.

**Figure 2:**
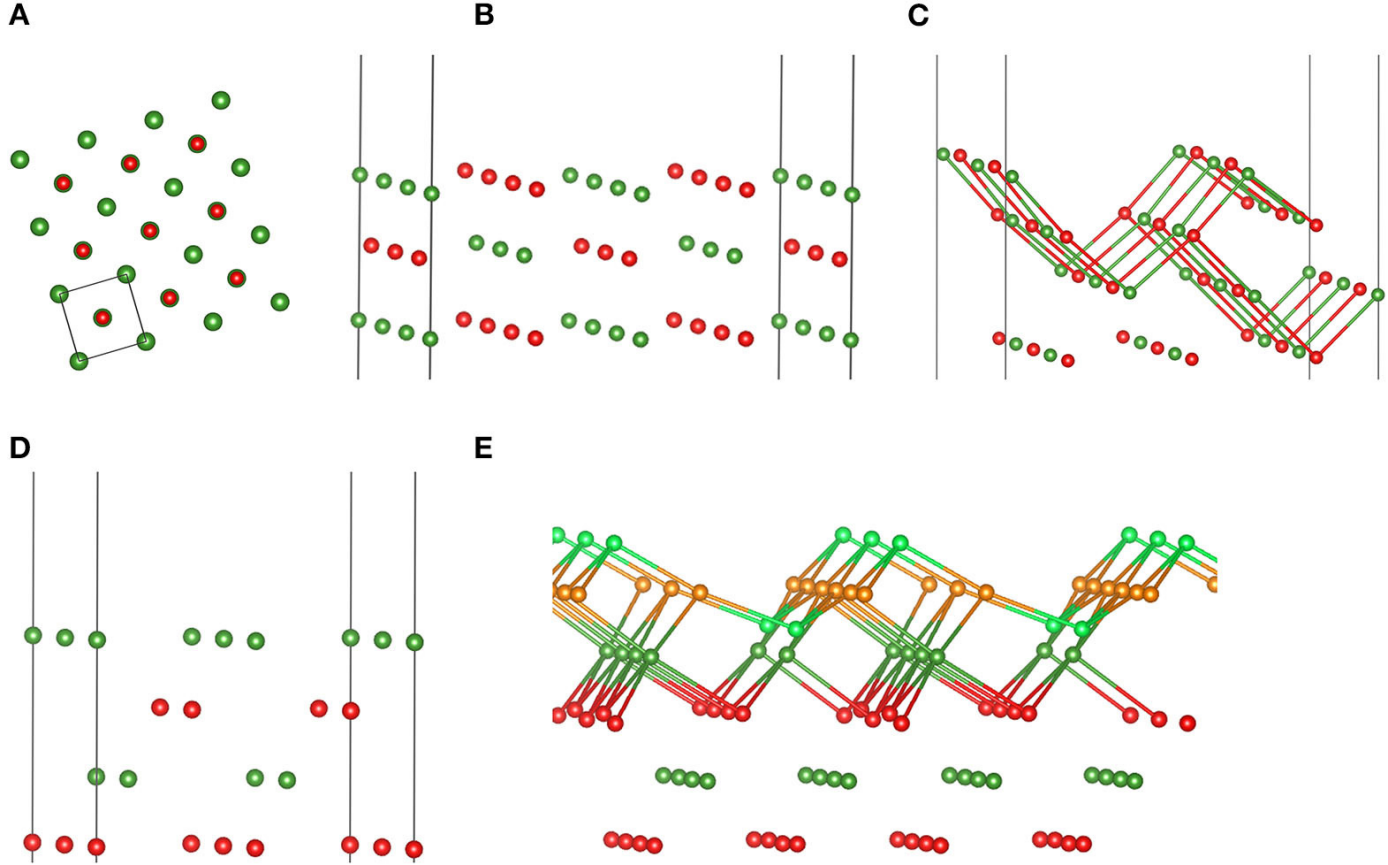
Structure models of the (001) surface in top view **(A)**, the (110) **(B)** and facetted (110) surfaces **(C)** in perspective view, the Mg-terminated (111) surface in perspective view **(D**), as well as the (2 × 2) octopolar reconstructed (111) MgO surface **(E)** employed to assess MLFFs. **(C,D)** display bonds with a maximum length of 2.1 and 2.08 Å, respectively. Color code is the same as in Figure 1, except for Mg in light green and O atoms in orange to indicate optimized (001) facets (see also Watson et al., 1996, and Pojani et al., 1997).

To test transferability or, in ML terminology, generalizability of these MgO FFs, we applied them to various MgO surfaces, which are shown in Figure 2. It is well known from experiment and theory that MgO prefers to expose the (001) cut, as thereby obtained surfaces are uncharged and nonpolar (Tasker, 1979; Noguera, 2000). This results in low surface energies. However, also cuts along (110) and (111) are possible, but they prefer reconstructions formed out of stabilizing (001) facets or—in case of (111)—the so-called (2 × 2) octopolar reconstruction (Gajdardziska-Josifovska et al., 2002). More details on these known reconstructions can be found in Watson et al. (1996), Pojani et al. (1997), and Kuhlenbeck et al. (2013), as well as therein cited references.

Table 2 summarizes the obtained results, i.e., PBE, FF_bulk_, and FF_cluster_ surface energies for MgO surfaces. We repeat that the DFT-PBE results serve as a reference and are thus highlighted in bold print. Interestingly, the performances of both FFs are quite similar based on the overall relative errors given in %. Clearly, because of the vast overestimation of the energy of the (100) surface by FF_cluster_, FF_bulk_ appears to perform slightly better. This is because the errors in the (100, 110), and facetted (110) surface energies are ca. 4, −13, and −6%, respectively. Both FFs correctly predict the (100) surface as most stable, and the (111) surface as least stable surface. The PBE surface energy difference between (100) and (111) is 0.293 eV/Å^2^, whereas FF_bulk_ predicts 0.198, and FF_cluster_ predicts 0.142 eV/Å^2^ as the “stability gap” between most and least stable surface. Hence, both FFs underestimate this energy span significantly, but less by FF_bulk_. Another aspect is the conspicuously large error in the (100) surface energy obtained with the cluster FF. To test the effect of restarting the training of the MLFF using a cluster with a similar atomic structure of the (100) facet as featured by cluster **E** (Figure 1), we also provide these additional results in Table 2. Apparently, the relative error for the (100) surface energy improves satisfactorily, but we sacrifice accuracy in the description of (110) – fac, (111), and the (111) – (2 × 2) surface energies. It seems that similarity in “local structure patterns” improves transferability of FFs from finite clusters to extended surfaces. Nonetheless, FF_bulk_ clearly outperforms FF_cluster_ and FF_restart−E_.

**Table 2:** MgO surface energies (eV/Å^2^) obtained using PBE (as the reference, printed in bold), FF_bulk_, and FF_cluster_.

**γ|eV/Å^**2**^**	**(100)**	**(110)**	**(110)–fac**	**(111)**	**(111) – (2 × 2)**
PBE	**0.053**	**0.133**	**0.101**	**0.346**	**0.137**
FF_bulk_	0.055 (3.78%)	0.116 (−12.8%)	0.095 (−5.9%)	0.253 (−26.9%)	0.104 (−24.1%)
FF_cluster_	0.120 (126%)	0.165 (24.1%)	0.129 (27.7%)	0.262 (−24.3%)	0.163 (19%)
FF_restart−E_	0.048 (−10.9%)	0.105 (−21.2%)	0.057 (−43.6%)	0.221 (−36.2%)	0.099 (−27.9%)

*Relative errors are presented in parenthesis*.

### MLFF for FeO_x_ Clusters and Surfaces

**Figure 3:**
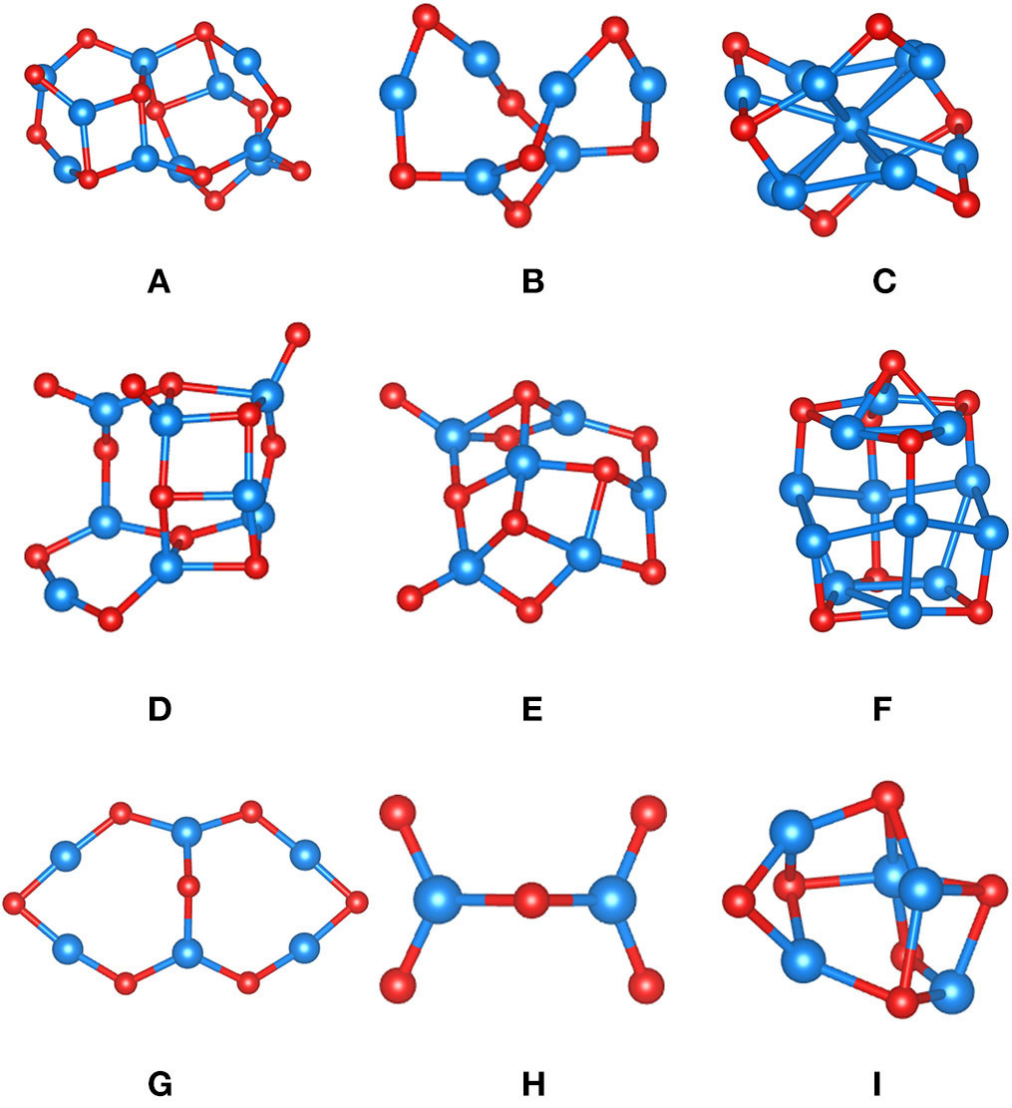
FeO_x_ clusters **(A–I)** optimized using PBE and used to assess MLFFs. Color code: Fe is blue, and oxygen is red.

Figure 3 displays the various FeO_x_ clusters used to assess our MLFF (FF_slab_) for FeO_x_ systems. To increase complexity, the test set contains clusters of varying composition, complying with the property of iron oxide to form various phases of distinct composition. For instance, clusters **C** and **F** are particularly Fe-rich; cluster **H** (composition Fe_2_O_5_) is an O-rich cluster, representing strongly oxidized Fe. Because of that, we cannot provide energies or energy differences per formula unit, as done for MgO clusters (Table 1), but summarize formation energies per Fe ion instead (Table 3). PBE predicts that Fe is rather oxophilic, which agrees with observation (“iron rusts”) and work published in the literature (Kepp, 2016). It is, for instance, seen in the strongly exothermic formation energy of cluster **H**. The MSE is −0.20 eV/Fe, and the MUE is 0.32 eV/Fe, comparable to corresponding results for MgO clusters (Table 2). Overall, we claim that the ordering of clusters by formation energy/Fe atom is fair, given that the most and least stable clusters are correctly predicted. The energy window spanned by them is 7.87 eV/Fe in case of PBE, and it is 8.41 eV/Fe predicted by FF_slab_. The ordering in terms of stability of clusters **B**, **G**, and **I** is incorrect, but we underline that differences in eV/Fe for these three clusters are within ca. 0.1 eV, which is substantially below the statistical deviation between the PBE reference and FF_slab_. In other words, these clusters are too close in stability to be correctly described by our MLFF.

**Table 3:** Formation energies (ΔE in eV/Fe) obtained using (spin-polarized) PBE—as the reference—and an MLFF, which was trained using a 5L symmetric slab model (see text).

	**A**	**B**	**C**	**D**	**E**	**F**	**G**	**H**	**I**
	**{Fe_**10**_O_**12**_}**	**{Fe_**6**_O_**7**_}**	**{Fe_**11**_O_**6**_}**	**{Fe_**8**_O_**12**_}**	**{Fe_**6**_O_**10**_}**	**{Fe_**12**_O_**8**_}**	**{Fe_**6**_O_**7**_}**	**{Fe_**2**_O_**5**_}**	**{Fe_**5**_O_**6**_}**
ΔE^PBE^	−10.12	−9.65	−6.65	−11.18	−11.96	−7.34	−9.61	−14.52	−9.54
Order	**4**	**5**	**9**	**3**	**2**	**8**	**6**	**1**	**7**
FF_slab_^a^									
ΔE	−9.92	−9.71	−6.83	−11.16	−12.99	−7.49	−9.25	−15.24	−9.75
Order	**4**	**6**	**9**	**3**	**2**	**8**	**7**	**1**	**5**
MSE	−0.20								
MUE	0.32								

*The row “order” (bold values) refers to the order of clusters by relative energies ΔE, with 1 as the most stable and 9 as the least stable cluster. MSE, mean signed error; MUE, mean unsigned error*.

a *Structure optimized*.

Next, we describe the similarity of PBE structures and structures obtained after optimization using FF_slab_. Using this FF for structure optimizations, half of clusters could be straightforwardly converged. However, even for clusters **D** and **E**, whose structure optimization appeared to be more involved, atomic connectivity and shape of the clusters agree well with PBE results. Note that the agreement between PBE and FF_slab_ bond lengths may vary, as observed for MgO clusters, depending on the type of bond. For instance, some bridging O-Fe-O bond distances may be off by ca. 10%, but dangling or terminal Fe-O bonds may differ by 1% only. The geometric structures of clusters **C**, **G**, and **H** obtained using FF_slab_ are remarkably close to PBE results (Supplementary Material).

**Figure 4:**
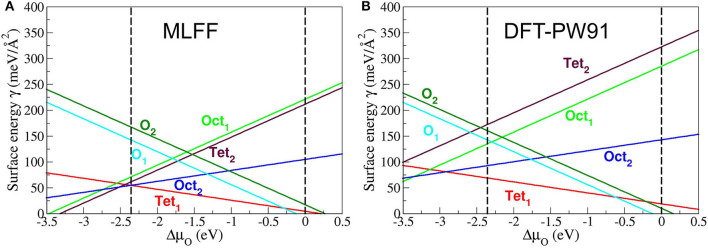
Stability diagrams for the six (ideal) bulk terminations of Fe_3_O_4_(111). **(A)** Diagram obtained using FF_slab_ (see text). **(B)** Diagram obtained using the PW91 functional, which is supposed to perform similarly compared with PBE. **(B)** is redrawn with permission from Kiejna et al. (2012). Copyright (2020) by the American Physical Society.

To assess the generalizability of FF_slab_ to extended FeO_x_ systems like surfaces, we applied it to the six ideal bulk terminations of the (111) surface of magnetite, Fe_3_O_4_. Figure 4 shows two stability plots, i.e., surface (free) energy as a function of the variation of the chemical potential of oxygen, Δμ_O_. The latter is referenced to $\frac{1}{2}E_{O2}$ being half of the total electronic PBE energy of the O_2_ molecule, $\Delta \mu_{O}=\mu_{O}-\frac{1}{2}E_{O2}$ (Reuter and Scheffler, 2001). The vertical dashed lines indicate the thermodynamically meaningful range of Δμ_O_. The lower limit at about −2.5 eV refers to the reduction or decomposition of Fe_3_O_4_ to metallic Fe, and the upper limit at 0 eV corresponds to the condensation of oxygen on the magnetite surface. These limits define “strongly reducing” as well as “strongly oxidizing” conditions. Within this interval, the sequence of the six surface terminations in terms of stabilities follows the one predicted by DFT-PW91 (Figure 4, R.H.S.), except for the two least stable terminations, namely, Fe_oct1_ and Fe_tet2_. These are Fe-rich terminations, contrary to the two most stable Fe_tet1_ and Fe_oct2_ surface terminations. The latter surface structures consist of one and two Fe ions per primitive surface unit cell, respectively. Therefore, Fe_tet1_ and Fe_oct2_ are also known as single- and double-metal terminations. Note that the graph based on the PW91 xc functional is redrawn after Kiejna et al. (2012). Besides the aforementioned qualitative differences, FF_slab_ also predicts quantitative changes affecting the coexistence regions of the Fe_tet1_ and Fe_oct2_ terminations as a function of Δμ_O_. Compared to DFT-PW91 results, FF_slab_ predicts the Fe_tet1_ termination as slightly too stable, while the Fe_oct2_ (Fe-rich) termination is even more stabilized by FF_slab_. Consequently, the coexistence point for these terminations is shifted by ca. 0.5 eV toward more “oxidizing conditions,” i.e., more positive values of Δμ_O_. Given that the FF calculations were carried out within seconds, obtained numerical results are of satisfactory accuracy. This especially refers to the metal-terminated ground state surface structures, as well as both oxygen terminations O_1_ and O_2_.

**Figure 5:**
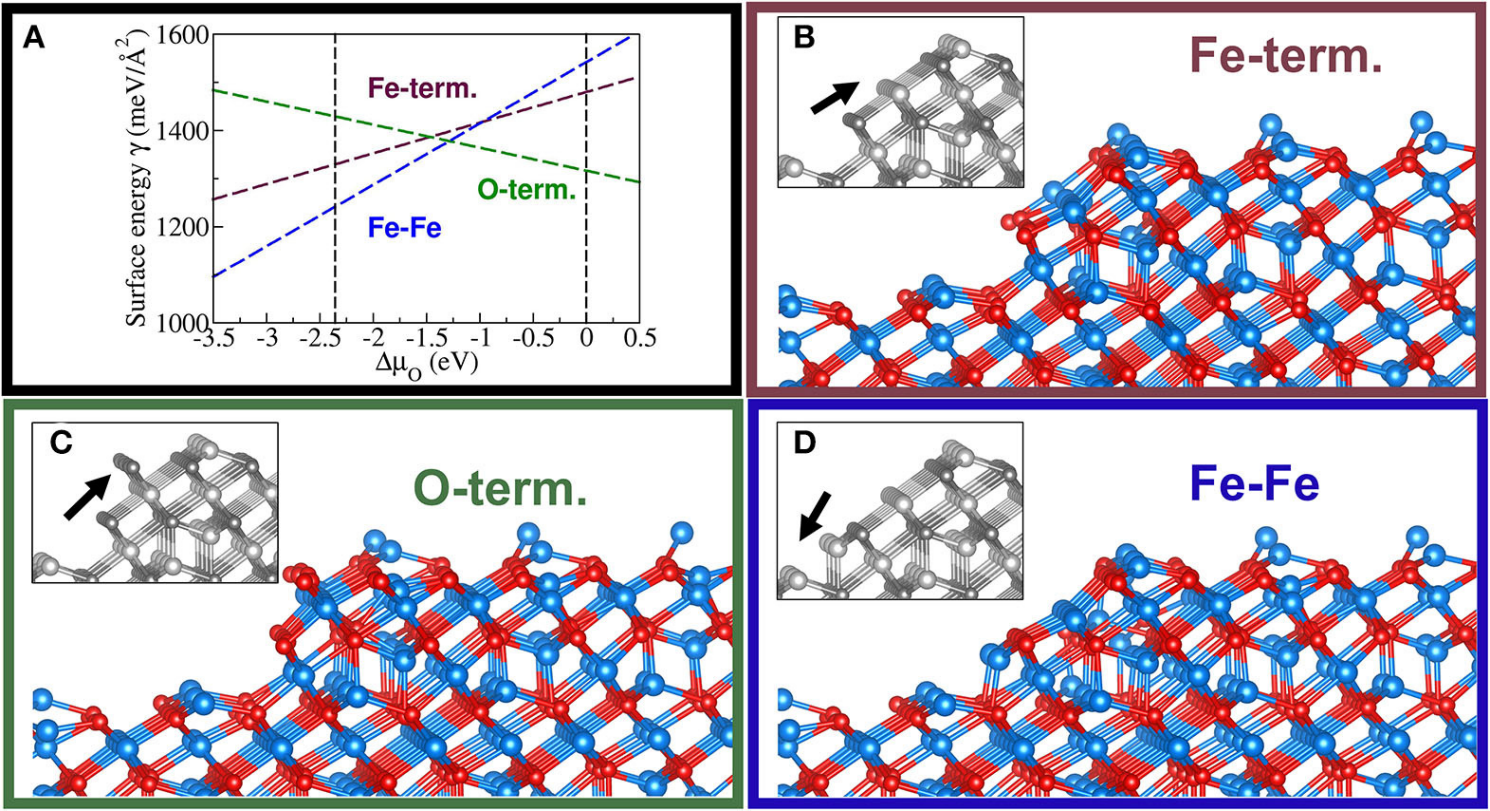
Stability diagram for three terminations of a step of one-repeat unit (i.e., six atomic layers) in height for the Fe_tet1_-terminated Fe_3_O_4_(111) surface **(A)**. Additionally, a Fe_oct_-terminated facet **(B)**, an O-term. facet **(C)**, and a supposedly unstable step having two consecutive low coordinated Fe-rows **(D)** are shown. The black arrow in the gray-colored inset, representing the unrelaxed structure, indicates these distinct features of the step models. Same color code as in Figure 3.

Again, based on calculated stabilities of the ideal bulk terminations of the (111) surface of magnetite, we conclude that FF_slab_ can be applied to larger FeO_x_ systems featuring similar local structure patterns. Figure 5 shows a stability plot, as well as corresponding step models on the Fe_3_O_4_(111) surface. We emphasize that calculations using DFT for these large systems are computationally unfeasible (currently). Note that the MLFF predicts a coexistence region centered at ca. −1.2 eV for the chemical potential of oxygen.

### Water on MgO(001)

The story about water overlayers on the ideal (001) surface of MgO is long. Therefore, we refer to a quite comprehensive overview in Wlodarczyk et al. (2011), a work that studied a low temperature *c*(4 × 2) structure containing 10 water molecules and a high temperature *p*(3 × 2) overlayer structure containing six water molecules. In both cases, some of the water molecules dissociate and hence hydroxyl groups are formed on the surface. Here, we are interested in the problem whether our MLFF let the water molecules (partly) dissociate on the MgO(001) surface, or conversely, whether the water molecules stay intact. The first case is associated with formation of metal M-O_W_H hydroxyl groups and (necessarily) formation of O_S_H hydroxyl groups in the oxide surface (subscripts W and S stand for water and surface, respectively). Regardless of theory, the latter case indicates a stronger water–water interaction involving water agglomeration (Thiel and Madey, 1987; Henderson, 2002). Dissociation of water on MgO(001) films has been claimed in the early 1990s of the past century (Wu et al., 1992) based on high-resolution electron energy loss spectroscopy. Early simulations using interatomic pair potentials found that the MgO(001) surface is generally not amenable to dissociative adsorption of water (de Leeuw et al., 1995), although this work clearly stated water dissociation as a function of coverage. The situation changes completely in presence of defects like undercoordinated Mg ions on the surface (Chizallet et al., 2006).

**Figure 6:**
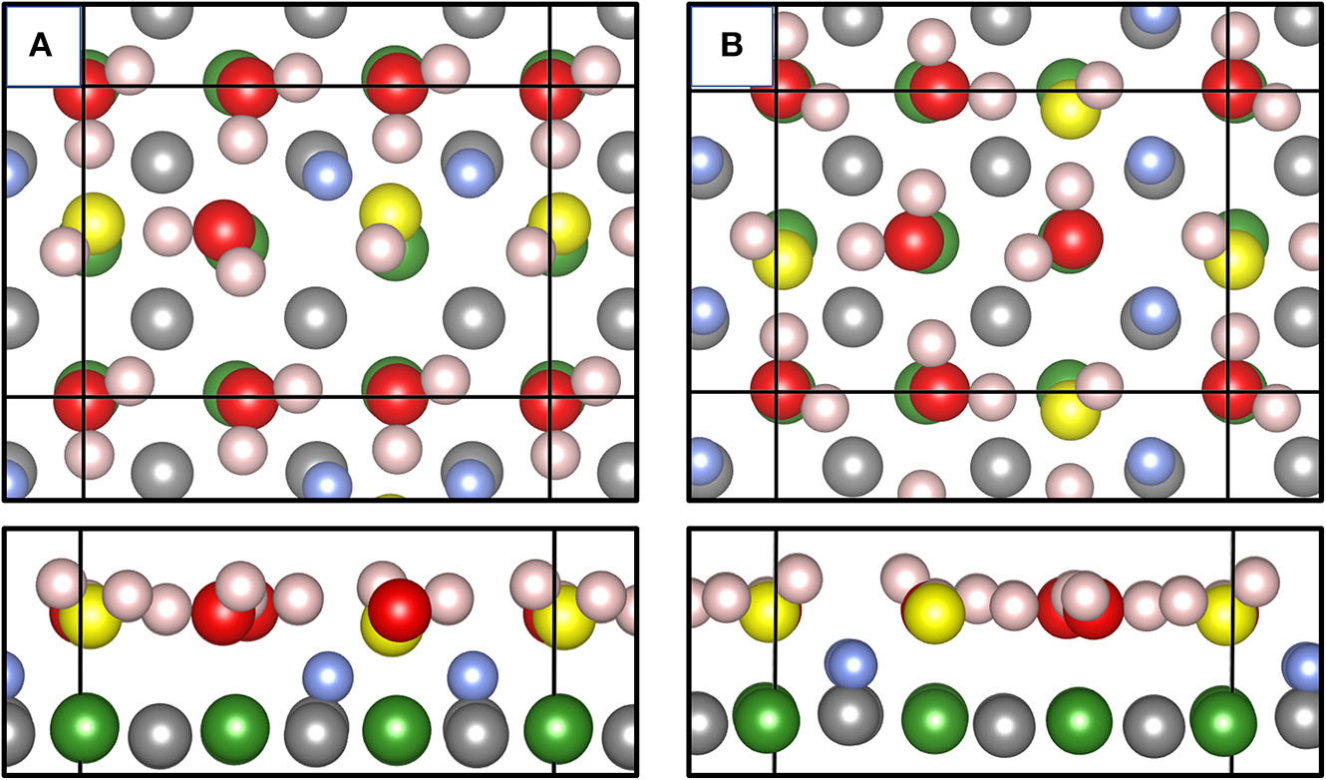
Top and side views of the adsorbate layer consisting of 6 H_2_O molecules on a *p*(3 × 2) MgO(001) surface generated via MD simulation at 400 K and optimized using PBE **(A)**. Top and side views of the global energy minimum structure published in literature (Wlodarczyk et al., 2011) **(B)**. Color code: Mg ions are green, H in surface OH is blue, O in intact H_2_O molecules is red, O in terminal, 1-coordinated OH atop Mg ions are yellow, and surface O ions are gray.

Figure 6 shows some important results of our MLFF-MD simulations thermostatized at 400 K. We used a *p*(3 × 2) MgO(001) surface unit cell and put six undissociated (intact) water molecules on the surface. Running the MD simulation for 2,000 time steps (2 ps), already after 0.2 ps two H_2_O molecules (per supercell) were dissociated and apart from thermal fluctuations, the water structure essentially remained during further 4,000 steps (6 ps in total). We optimized this “structure snapshot” using the PBE functional (Figure 6A). Figure 6B shows the global energy minimum structure published in Wlodarczyk et al. (2011). This structure has been obtained using a genetic algorithm employing PBE and Grimme's D2 correction for dispersion effects. Optimizing the structure found via MLFF-MD using the PBE functional, we found it only 0.234 eV higher in total energy compared with the PBE result for the global minimum, i.e., only 0.04 eV/H_2_O. However, our structure misses one important characteristic, namely, the observed glide plane. Figure 6B meets this criterion as realized by the relative configurations and orientations of surface OH groups (light blue) and the terminal OH groups (yellow) originating from dissociated H_2_O molecules. In Figure 6B, these groups together with intact water molecules fulfill the translation–reflection operations occurring in glide planes. On the quality of some characteristic geometric structure parameters such as Mg-O_W_H, O_w_H, or O_S_H distances obtained with MLFF, we found only moderate deviations from PBE results. If one were interested in MLFF-derived properties such as vibrational frequencies, corresponding wavenumbers would be severely affected (Badger, 1934), but this is beyond the scope of the present work.

Supplementary Figure 2 shows three snapshots taken after 2, 4, and 6 ps of an MLFF-MD simulation at 400 K. Within this timescale, exchange of the two surface OsH groups shown in light blue and stemming from dissociated H_2_O molecules did not occur. Instead, protons in the overlayer structure jumped following the well understood Grotthuß mechanism. This is indicated by changing positions of terminal surface O_W_H groups (yellow) binding to Mg^2+^ ions. The snapshot taken after 6 ps (Supplementary Figure 2C) shows a transient proton jump from one water species to another one.

Supplementary Figure 3 addresses so-called finite size effects in MD simulations. For this purpose, we carried out MLFF-MD simulations at 280 K for the *p*(3 × 2) and a *p*(6 × 4) supercell of 6 and 24 molecularly adsorbed H_2_O molecules, respectively. Note that the initial H_2_O overlayer structures (i.e., coverages and atomic positions) were identically generated multiplying periodic directions by a factor of 2. Results obtained using the same MLFF after 2,000 time steps (0.4 ps) are shown. In other words, we did not retrain the MLFF using the *p*(6 × 4) supercell. It is noteworthy that the *p*(6 × 4) supercell (Supplementary Figure 3B) contains nine surface O_S_H groups, as well as an additional one, which is just about to be created. This is indicated by a 20% longer bond distance to the surface O ion as compared to its equilibrium position. We mention that the MLFF is capable to describe distinct scenarios in terms of overlayer structures. While in the *p*(3 × 2) supercell (Supplementary Figure 3A) predominantly linear chains of H_2_O molecules are formed, the *p*(6 × 4) cell allows for the creation of circular motifs, such as pentamers and even larger structures. A more detailed analysis will be provided elsewhere.

### Water on Fe_3_O_4_(111)

The interface between water and iron oxides have enjoyed great attraction by many researchers over passed years (Joseph et al., 2000; Leist et al., 2003; Merte et al., 2012; Dementyev et al., 2015; Meier et al., 2018; Mirabella et al., 2018; Zaki et al., 2018; Schöttner et al., 2019; Li and Paier, 2020). Nonetheless, the situation is substantially more complicated compared to MgO. Many efforts are currently spent to develop approaches that are low on computational workload, like cost-efficient semiempirical methods (Liu et al., 2020a) or FF-type of approaches (Cygan et al., 2004). The big problem rests on thereby attained accuracies especially with respect to calculations or simulations of properties of the adsorbed water layers. These properties such as vibrational frequencies depend critically on the correct description of (the various) bonding interactions within the water/oxide system, and hence the conventionally applied method is the so-called DFT+U approach employing a Hubbard-type correction for correlated Fe 3d orbitals [for DFT+U applied to iron oxides, see Meng et al. (2016)]. Recent studies by Hermansson and Behler using so-called neural network potentials derived from DFT, i.e., RPBE+D3, examined anharmonic vibrational frequencies of water on ZnO (Quaranta et al., 2018) and could thus do simulations on a large system involving even bulk water on the surface.

Regarding iron oxides, complexity grows due to essentially two reasons: (i) atomic structures of iron oxide surfaces are extremely diverse because of the many competing phases, for instance, FeO (rock salt), Fe_3_O_4_ (inverse cubic spinel > *T*_Verwey_ ≈ 125 K, monoclinic < *T*_Verwey_), and Fe_2_O_3_ (hematite); these different phases are usually associated with surfaces of many possible surface terminations, which may involve defects (Novotny et al., 2013; Bliem et al., 2014); (ii) iron oxides feature complex electronic as well as magnetic structures, due to the multivalent Fe ions, usually Fe(II) and Fe(III), which may distinctly affect the water adsorption process or bonding interactions.

**Figure 7:**
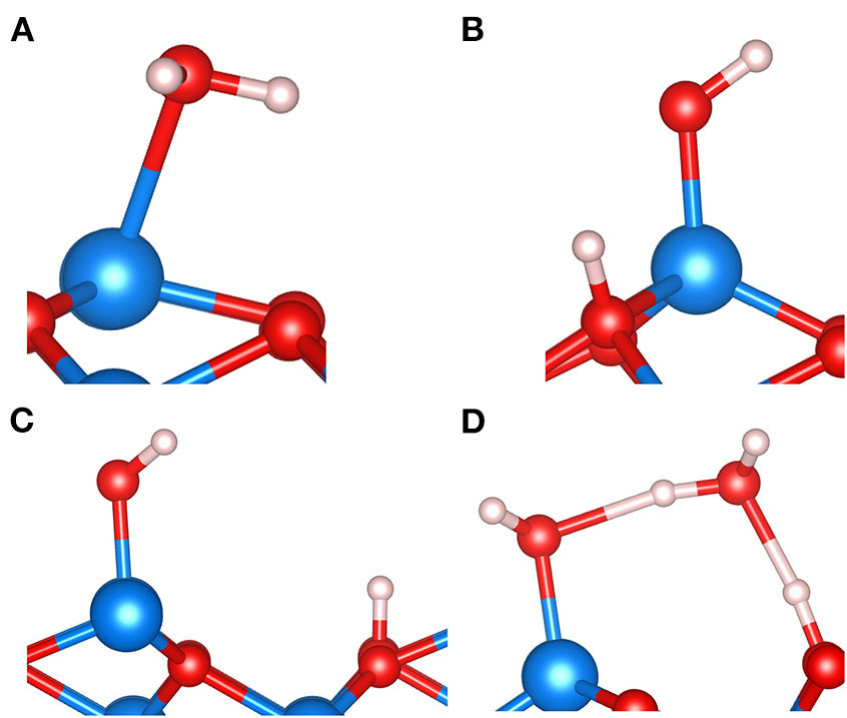
Structure motifs involving H_2_O molecules adsorbed on the *p*(1 × 1) – Fe_3_O_4_(111) surface. Molecular adsorption **(A)**, dissociative adsorption featuring a so-called O_a_H surface hydroxyl group **(B)**, dissociative adsorption exposing an O_b_H surface hydroxyl group **(C)**, and the H-bonded so-called half-dissociated H_2_O dimer **(D)**. Color code: Fe is blue, O is red, and H is white.

Figure 7 shows four basic motifs potentially formed when water adsorbs on the single-metal Fe_tet1_-terminated (111) surface of the inverse spinel magnetite, Fe_3_O_4_. Note that there exist two competing, i.e., similarly stable, surface structures that involve either one or two Fe ions per unit cell in the outermost atomic layers of Fe_3_O_4_ (111). As discussed in the previous section on FeO_x_ clusters and surfaces, the Fe ion of the single-metal termination corresponds to (in bulk phase) a fourfold or tetrahedrally coordinated Fe ion. This is commonly abbreviated as Fe_tet1_ because there are two possible Fe_tet_ layers to cut the surface normal to the [111] direction. The double-metal termination consists of an Fe_tet1_ ion and an Fe_oct2_ ion, where the latter one is sixfold or octahedrally coordinated in the bulk phase. We have chosen to show these motifs for the single-metal termination of magnetite (111), because there is ample evidence generated from observation, as well as from theory, that under certain preparation conditions of magnetite (111) films, the Fe_tet1_ termination represents the regular (majority) domains of the surface (Sala et al., 2012; Li et al., 2018; Liu et al., 2020b). Figure 7A shows the molecular adsorption of water. The PBE+U results suggest that molecular and dissociative adsorption modes as displayed in Figures 7B,C are comparably stable (Li and Paier, 2016), but they also show that there is a clear preference for dissociation. Note that the (111) surface of magnetite exposes two distinct O ions. Per primitive surface unit cell, three oxygen ions bind to the surface Fe ion in (local) C_3v_ symmetry, and the fourth one is not bound to the surface Fe. Notations such as O_a_ and O_b_ have been introduced to discriminate between these two oxygen ions (Grillo et al., 2008). We use O_a_ for Fe-bound O ions in the surface and O_b_ for the remaining oxygen ion, not bound to the surface Fe. The four surface O ions per primitive unit cell form together a close packed layer. PBE+U predicts that upon dissociation of one H_2_O molecule per unit cell, protonation of O_a_ is slightly more favorable than protonation of O_b_. We find an energy difference of 0.08 eV only, which is small but comparable in magnitude as obtained for the dissociative adsorption of methanol (CH_3_OH) in a *p*(2 × 1)–Fe_3_O_4_(111) surface unit cell (Li and Paier, 2019). As discussed in Li and Paier (2019), this energy difference is small and depends on the electronic structure method. For the dissociative adsorption of water on the *p*(1 × 1) – Fe_3_O_4_(111) surface employing the HSE hybrid functional, we find that protonation of the O_b_ oxygen ion is by ca. 0.1 eV more stable compared to protonation of O_a_.

Turning to the performances of MLFFs, we found that learning or training FFs on-the-fly using MD simulations for the H_2_O/Fe_3_O_4_(111) system is a nontrivial process. This is already realized by the significantly increased computational cost (i.e., one order of magnitude) compared to, e.g., H_2_O/MgO(100) calculations discussed in the previous section. Moreover, it is also more difficult to obtain sufficiently small Bayesian errors, which in turn requires long simulations. We tried to avoid desorption events, which happens easily especially for higher loadings of water, e.g., three or four molecules per primitive surface unit cell. Here, lower simulation temperatures of ca. 150 K and adding a correction for van der Waals–type dispersion interactions such as the Grimme D2 approach helped to keep the molecules on the surface.

**Figure 8:**
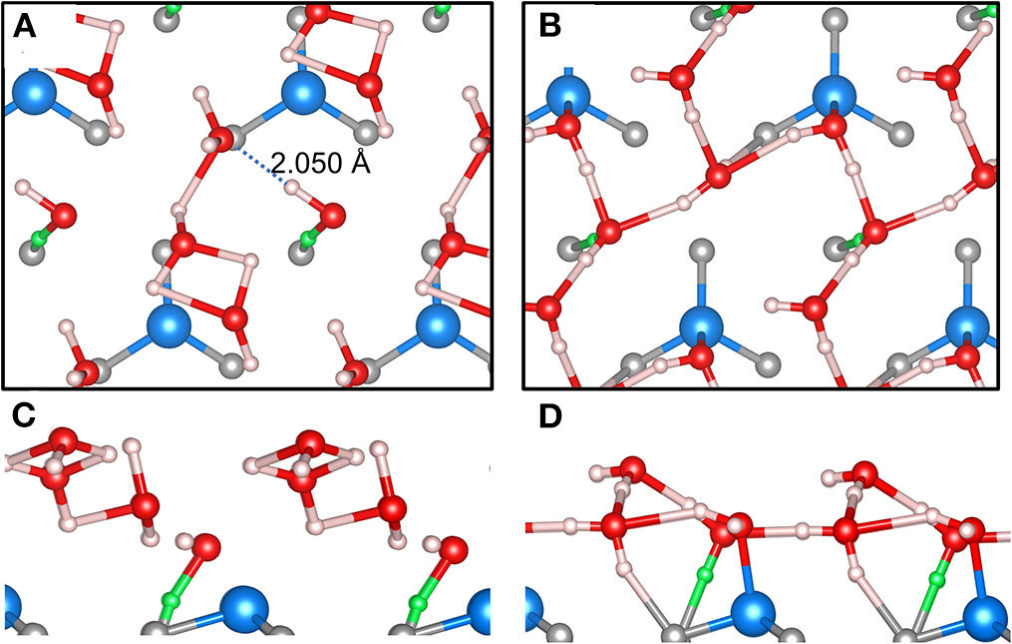
MLFF **(A)** and PBE+D2 **(B)** optimized snapshot after 400 fs of an NVT MD simulation at 150 K starting from 4 intact D_2_O molecules on the *p*(1 × 1) – Fe_3_O_4_(111) surface. Corresponding side views **(C,D)** are shown below. Color code: Fe is blue, O is red, H is white, and in surface OH groups, H is green. OH bond distances less than or equal to 2 Å are drawn as bicolor cylinders.

While the studies published in Dementyev et al. (2015), Mirabella et al. (2018), and Zaki et al. (2018) focused on the low-coverage regime of H_2_O adsorption on Fe_3_O_4_(111), we examine in this work the challenging case of high loading, i.e., 4 H_2_O molecules per primitive surface unit cell. This amount of water corresponds to a (nominal) coverage of 4 ML, because one assumes one dissociatively bound H_2_O molecule per surface Fe ion (Dementyev et al., 2015). The big difference to Figure 7 is that the global energy minimum for this case is unknown. Therefore, we focus on differences in performance of our MLFF and the PBE+D2 functional. Figure 8A shows an MLFF-optimized snapshot of an *NVT* ensemble MLFF-MD simulation at 150 K and stopped after 2,000 steps (= 0.4 ps). We reiterate, that this structure was subsequently optimized using MLFF (Figures 8A,C), as well as PBE+D2 (Figures 8B,D).

Although (partial) creation of one surface O_S_H per unit cell was found (green OH), this dissociative event does not involve the Fe site, contrary to chemical intuition and previous DFT calculations (Li and Paier, 2016; Ovcharenko et al., 2016). Instead, as shown by the dashed line in Figure 8A, an H-bond to a neighboring H_2_O molecule is formed, which likely triggers dissociation because it has been learned in the MLFF-MD run that dissociated dimers are competing stable motifs (see below), but ignored the causal relation to Fe ions. A second aspect or shortcoming of our MLFF is that water molecules agglomerate or dimerize too strongly compared to the PBE+D2 structure (Figure 8B). This is for instance seen on the formation of H-bridged OHOH motifs. Note that H-bonds with a length of ≤2.0 Å are drawn. These cyclic motifs involve a proton “donor-acceptor” O-H…O bond angle of ca. 90°, which is conspicuously small compared with the commonly observed angle in H-bonds of ca. 180°. The H-bond is considered as the most directional of noncovalent interactions, although there is some flexibility within ±20 to ±30° (Israelachvili, 2011). The MLFF clearly does not get the O-H…O angle right.

To analyze these shortcomings in MLFF-based MD simulations for (4 × H_2_O)/Fe_3_O_4_(111) – (1 × 1), we stepped back and carried out an MD run using one molecularly adsorbed H_2_O as a starting point employing the MLFF trained on the dissociatively adsorbed H_2_O monomer (Figure 7C). The Bayesian errors after training using 2,000 × 1 fs time steps were 0.042 eV/atom. However, after 0.4 ps MLFF-MD simulation time, we found the intact H_2_O molecule dissociated away from the Fe ion. Increasing the temperature to 400 K and restarting the MD run for 0.6 ps did not yield dissociatively adsorbed water. We consider this as an indication that sufficient attractive interactions are missing to bind the molecule to the Fe ion. We emphasize that all MLFF-MD training simulations based on PBE+D2 involved adsorption of (dissociated) water on the Fe ion as a regularly occurring event. Visual inspection of trajectories clearly showed dissociation of H_2_O at the Fe ion. For the MLFF training, we carried out 10,000 MD steps in total, as done for H_2_O/MgO(100). Each of these simulations successfully finished with Bayesian errors of 0.05 – 0.04 eV/atom. To examine the numerical accuracy required to discriminate H_2_O adsorption structures in the high-coverage limit, we reoptimized the PBE+D2 structure for (4 × H_2_O)/Fe_3_O_4_(111) – (1 × 1) shown in Figure 8B using the MLFF. The difference in the total energies is 0.218 eV or 0.054 eV/H_2_O molecule. Converted into the “Bayesian scale” of eV/atom it is exactly one order of magnitude smaller, i.e., 0.0054 eV/atom. Clearly, this is far below the prediction error and results in the fact that the MLFF cannot discriminate between the two structures shown in Figure 8.

In addition, we further examine the description of the potential energy surface using the MLFF and compare it to the underlying PBE as well as PBE+D2 results. Supplementary Table 1 shows corresponding adsorption energies for dissociated and molecularly adsorbed water structures. These results show that PBE does not prefer dissociation over molecular adsorption. The D2 correction leads to a strong bias toward molecular adsorption for the one-molecule case, and consequently, this bias has been inherited to MLFF. For two molecules on the surface, MLFF even favors molecular adsorption by 0.15 eV. This finding suggests that the H_2_O–surface interaction misses important contributions, which would lead to (the expected) H_2_O dissociation on Fe ions. Certainly, these shortcomings accumulate into results as the one shown in Figure 8A. Our analysis demonstrates that the electronic structure method underlying the training of the MLFF plays a crucial role, as expected.

We conclude that the required accuracy of (at least) 0.005 eV/atom is so small that any approximation in the construction of the MLFF will critically affect its performance. These approximations are (i) missing long-range interactions (see *Models and Methods*), (ii) many-body short-range interactions, (iii) quality of the underlying electronic structure method, and (iv) the sampling density because many, fairly shallow, local minima need to be accurately resolved by virtue of densely sampled potential energy surfaces. These approximations need to be successfully addressed prior to an efficient, yet accurate description of many water molecules adsorbed on the magnetite (111) surface.

## Conclusions

This work explores practical aspects and transferability or generalizability of MLFFs following the GAP ansatz, which was efficiently implemented in the Vienna *ab initio* simulation package, VASP, recently. The generalized-gradient approximation to Kohn–Sham DFT after PBE serves as an electronic structure reference method for assessing these MLFFs. The latter were trained employing *NVT* ensemble MD simulations using PBE energies and forces (or gradients) as input for Bayesian regression. We were able to generate FFs for relatively small cluster models and successfully employed MLFFs in calculations on extended surfaces including reconstructions. It appears plausible that sufficient similarity in (local) structure patterns, e.g., coordination numbers, will cause generalizability from clusters to surfaces, at least to within acceptable error bars. This was tested—and confirmed—for MgO as well as FeO_x_ systems. Remarkably, as shown for Fe_3_O_4_(111) surface terminations, our FeO_x_-MLFF predicts stabilities of ground state structures in fair agreement with DFT-GGA reference results within reasonable ranges of the chemical potential of oxygen. By virtue of these FFs, much larger systems such as steps involving different kinds of defects, for instance, grain boundaries, can be studied. Simulations on these large FeO_x_ systems are currently computationally out of reach using DFT. However, we find—as many workers before us—that generating FFs for water–oxide interfaces is a nontrivial task. Our MLFF-MD simulations reproduce DFT results that water partly dissociates in the H_2_O–MgO(001) interface. This means that our MLFF outperforms conventional interatomic potentials, where it was found difficult to correctly dissociate water on the pristine MgO(001) surface. Certainly, MLFFs open routes for rapid exploration of structural candidates over wide phase spaces combining them, for instance, with evolutionary or genetic algorithms. Contrary to the H_2_O–MgO interface, we encountered difficulties in generating reasonable FFs for the H_2_O-Fe_3_O_4_(111) interface. After careful convergence of the MLFF-training process, H_2_O molecules agglomerate strongly, i.e., water–water interactions are too attractive, whereas the H_2_O-Fe interaction is not correctly described, i.e., no Fe-OH groups are formed. The latter has been observed, and it is suggested by more sophisticated electronic structure methods. Our numerical analysis showed that a very tight error control within a meV/atom range is required to describe the potential energy surface spanned by many water molecules on a complex surface like Fe_3_O_4_(111). The approximations involved in the construction of the MLFF, i.e., missing long-range and incomplete many-body short-range interactions, as well as the electronic structure method underlying the training runs, will critically affect its accuracy. Our work is encouraging, but calls for further research when applying ML techniques to complex water–oxide interfaces.

## Data Availability Statement

The original contributions presented in the study are included in the article/Supplementary Materials, further inquiries can be directed to the corresponding author/s.

## Author Contributions

XL analyzed results and wrote some sections of the manuscript. WP wrote some sections of the manuscript. JP conceptualized the research, carried out the calculations, interpreted results, wrote some sections, and edited the entire manuscript. All authors contributed to the article and approved the submitted version.

## Conflict of Interest

The authors declare that the research was conducted in the absence of any commercial or financial relationships that could be construed as a potential conflict of interest.
