# Supplementary material for: Machine Learning in Computational Surface Science and Catalysis: Case Studies on Water and Metal–Oxide Interfaces
Source: Front Chem. 2020 Nov 30;8:601029. doi: 10.3389/fchem.2020.601029 (PMC7793815; doi:10.3389/fchem.2020.601029)
Supplement: Supplementary file 1 [file Data_Sheet_1.PDF]

Supplementary Material

Machine Learning in Computational Surface Science and Catalysis: Case Studies on Water and Metal Oxide Interfaces

Xiaoke Li[1], Wolfgang Paier[2] and Joachim Paier[1]*

[1]Institut für Chemie, Humboldt-Universität zu Berlin, Unter den Linden 6, 10099 Berlin, Germany
[2]Fraunhofer Institute for Telecommunications, Heinrich Hertz Institute HHI, Einsteinufer 37, 10587 Berlin, Germany

*** Correspondence:**

Dr. Joachim Paier

joachim.paier@chemie.hu-berlin.de

Table of Contents

1. Surface Slab Model to Generate the FeO_x MLFF S2
2. H_2O/MgO(100)-(3×2) MD Snapshots S3
3. H_2O/MgO(100)-(6×4) MD Snapshots S4
4. H_2O/Fe_3O_4(111)-(1×1) Adsorption Energies S5
5. (2×H_2O)/Fe_3O_4(111)-(1×1) Dimer Structures S6
6. Coordinates of the Slab Model to Generate the FeO_x MLFF S7
7. Coordinates of MgO Clusters – PBE optimized S8
8. Coordinates of MgO Clusters – $MLFF_{cluster}$ optimized S14
9. Coordinates of FeO_x Clusters – PBE optimized S20
10. Coordinates of FeO_x Clusters – MLFF optimized S25
11. Coordinates of MgO Surfaces – PBE optimized S30
12. Coordinates of MgO Surfaces – MLFF optimized S41
13. Coordinates of Fe_3O_4(111) Surfaces – MLFF optimized S63
14. Coordinates of 6 H_2O-MgO(001)-(3×2) Surface – MLFF MD snapshot and PBE optimized S72
15. Coordinates of 4 H_2O-Fe_3O_4(111)-(1×1) Surface – MLFF MD snapshot and MLFF optimized S74
16. Coordinates of 4 H_2O-Fe_3O_4(111)-(1×1) Surface – MLFF MD snapshot and PBE optimized S75

1. Surface Slab Model to Generate the FeO_x MLFF

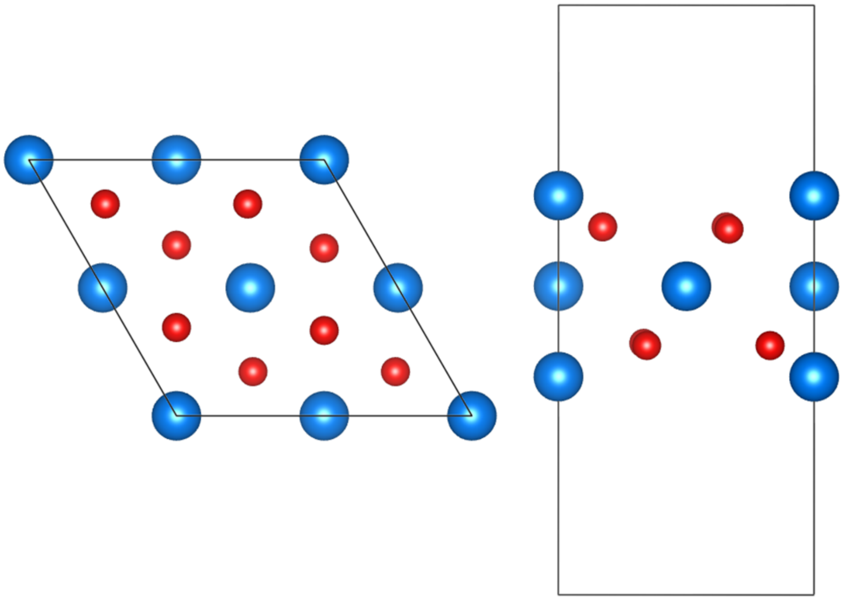

Figure S1: Symmetric structure model of the $Fe_3O_4(111)$ surface (Fe_{tet1} terminated). It consists of 5 atomic layers and was used to generate the FeO_x MLFF.

2. H_2O/MgO(100)-(3×2) MD Snapshots

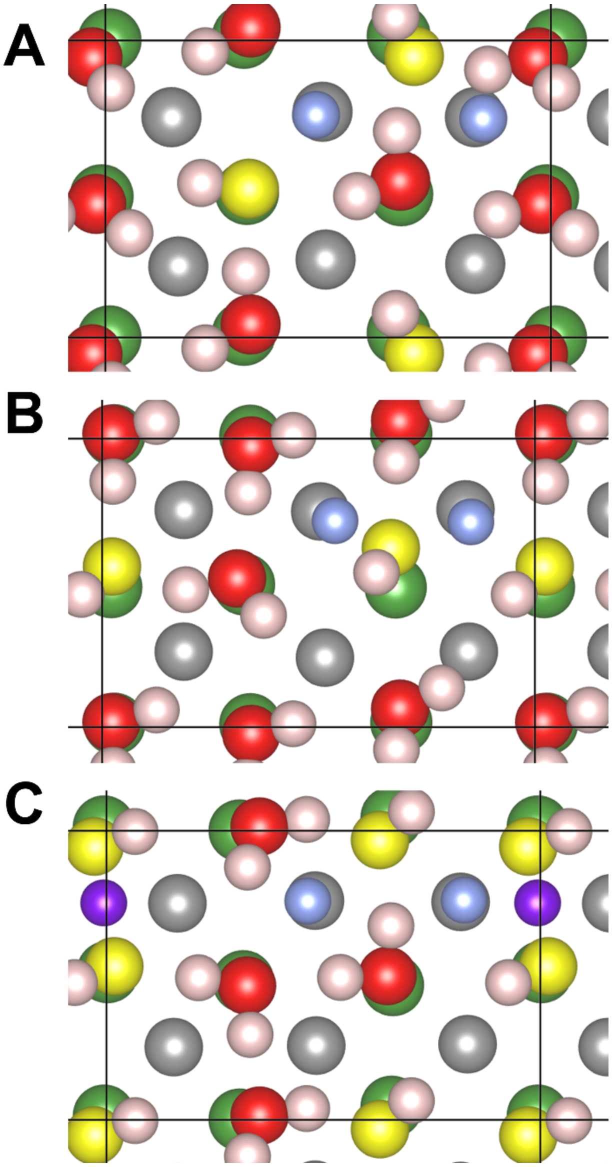

Figure S2: Molecular dynamics snapshots at 400 K (Nosé-Hoover thermostat) after 2000 (**A**), 4000 (**B**), and 6000 (**C**) time steps corresponding to 2, 4, and 6 ps using our MLFF for a *p*(3×2) supercell of H_2O/MgO(100). Note that surface OH groups (H is light blue) do not exchange, while protons jump as indicated by changing positions of the single-coordinated OH groups shown in yellow. Snapshot **C** at 6 ps shows a transient proton transfer (H^+ is violet).

3. H_2O/MgO(100)-(6×4) MD Snapshots

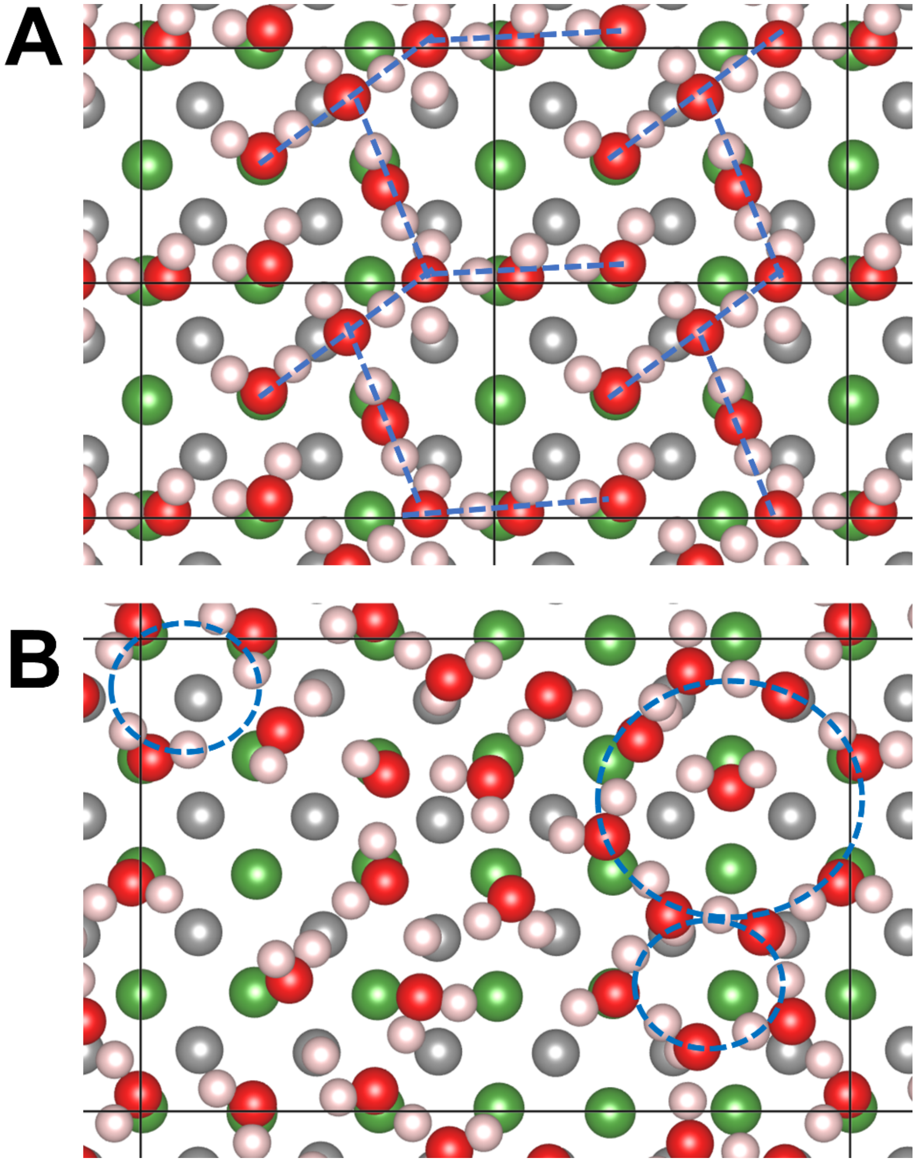

Figure S3: Molecular dynamics snapshots at 280 K (Nosé-Hoover thermostat) after 2000 time steps (0.4 ps) using our MLFF for a $p(3\times2)$ (**A**) and a $p(6\times4)$ (**B**) supercell of H_2O/MgO(100). For the discussion see main text. Overlayer structures are highlighted by dashed blue lines or cycles. Starting points, namely 6 and 24 intact H_2O molecules per supercell, have been identical, i.e. $p(6\times4)$ was obtained by multiplying periodic directions by a factor of 2.

4. H_2O/Fe_3O_4(111)-(1×1) Adsorption Energies

Table S1: Adsorption energies in eV/H_2O for the H_2O/Fe_3O_4(111)-(1×1) systems shown in Figure 7. In addition, results for the molecularly adsorbed dimer (2×H_2O-mol) are also shown. For the discussion, see main text.

	**MLFF**	**PBE**	**PBE+D2**[a]
H_2O-mol	-0.89	-0.36	-0.67
H_2O-dis (O_a)	-0.60	-0.11	-0.12
H_2O-dis (O_b)	-0.86	-0.35	-0.43
2×H_2O-mol	-1.21	-0.92	-1.06
2×H_2O-dis	-1.08	-0.91	-1.04

[a] Optimized structure using PBE+D2.

5. $(2\times H_2O)/Fe_3O_4(111)$-$(1\times1)$ Dimer Structures

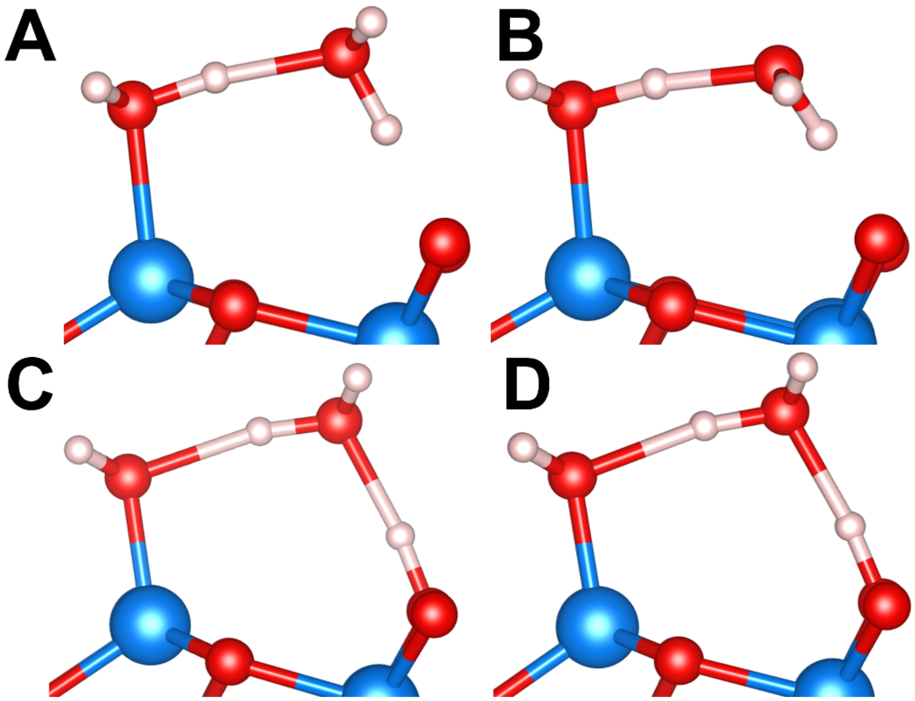

Figure S4: Molecularly (**A**, **B**) and dissociatively (**C**, **D**) adsorbed H_2O dimers on a $Fe_3O_4(111)$-(1×1) surface unit cell obtained using PBE (left) and PBE+D2 (right). Color code: Fe is light blue, O is red, and H is white.

6. Coordinates of the Slab Model to Generate the FeO_x MLFF

```
5L surface model – PBE+U(3.8) lattice parameters – VASP POSCAR format
1.0
 +6.0163998604  +0.0000000000  +0.0000000000
 -3.0081999302  +5.2103551184  +0.0000000000
 +0.0000000000  +0.0000000000 +12.0000000000
Fe O
 5 8
Cartesian
 +0.0000000000  +0.0000000000  +8.1233485480
 +0.0000000000  +0.0000000000  +4.4387830300
 +3.0081999300  +0.0000000000  +6.2807926090
 +1.5040999650  +2.6051775590  +6.2807926090
 -1.5040999650  +2.6051775590  +6.2807926090
 -0.0000301230  +3.4735875740  +5.1180784090
 -0.0000301230  +1.7981456510  +7.4871562560
 -1.4510052120  +4.3113082250  +7.4871562560
 +1.4509451450  +4.3113082250  +7.4871562560
 +3.0082298740  +3.4122094680  +5.0744289620
 +3.0082301430  +1.7367677000  +7.4440523560
 +4.4592050520  +0.8990467380  +5.0744289620
 +1.5572548750  +0.8990467380  +5.0744289620
```

7. Coordinates of MgO Clusters – PBE optimized

MgO_cluster A
1.00000000000000
10.0000000000000000 0.0000000000000000 0.0000000000000000
0.0000000000000000 10.0000000000000000 0.0000000000000000
0.0000000000000000 0.0000000000000000 20.0000000000000000
Mg O
8 8
Direct
0.5043385851859170 0.4097125563416529 0.3956423306521399
0.5653776193466890 0.6598986971679237 0.4078411371807670
0.6562812712463985 0.4856839000816177 0.5012510168796241
0.3753882316660077 0.5563319230206305 0.4897582733330146
0.4625686023858845 0.3661833481420553 0.5857237340664568
0.5314304625122125 0.6471331448507852 0.5992819061104707
0.3531921647397911 0.5250834016617417 0.6810566476789024
0.6033008332502076 0.4613482451717624 0.6914452132575886
0.6762384021905796 0.4999270390973964 0.4036364767587047
0.3951481245693867 0.5707595798769725 0.3920321333708293
0.4794169594983556 0.3696327476390130 0.4892322302482632
0.5519485301845202 0.6717602228259616 0.5038815738347278
0.6485299161303423 0.4687747121190758 0.5976485242239848
0.3458981505520386 0.5446673069369425 0.5852335507626176
0.4428484063483079 0.3522147959439710 0.6833335584946452
0.5121637481933614 0.6331683451224834 0.6969015531472562

MgO_cluster B
1.00000000000000
15.0000000000000000 0.0000000000000000 0.0000000000000000
0.0000000000000000 15.0000000000000000 0.0000000000000000
0.0000000000000000 0.0000000000000000 15.0000000000000000
Mg O
9 9
Direct
0.4475395684819275 0.3082095993489090 0.2540287539501296
0.5825951552864395 0.4090637644786158 0.2875728869105932
0.3328773433685551 0.2310522985668439 0.4602255453195134
0.4524012301205261 0.3970382172129611 0.4100474552603459
0.3454725277578675 0.3654532530544850 0.6104000761304027
0.5737346828406495 0.4633273835676489 0.5277936030528434
0.4459841433460722 0.5132321376010784 0.6474650847078086
0.3615473685306886 0.5232006191351253 0.8039982759258472
0.5730087513752805 0.6066625359062565 0.7320839205421614
0.5669727747596429 0.3079490631560652 0.2114243765404403
0.4533445373187139 0.4345394610269665 0.2845043228915792
0.4004156980921695 0.2758889680740729 0.3694695983898627
0.5896672858437061 0.4197913367987066 0.4143990505122455
0.2948293160226797 0.2600893208829583 0.5711929959123565
0.4406207424646184 0.4330901797639785 0.5414846131603371

0.5767841270645642 0.5383501005973289 0.6297459204163083
0.3467409224137423 0.4486450725414450 0.7076597813545149
0.4542141039121360 0.5958168922865333 0.7655038310226860

MgO_cluster C
1.00000000000000
15.0000000000000000 0.0000000000000000 0.0000000000000000
0.0000000000000000 15.0000000000000000 0.0000000000000000
0.0000000000000000 0.0000000000000000 15.0000000000000000
Mg O
8 8
Direct
0.3997447524798005 0.5130261140569968 0.3366817876019647
0.2497689171969028 0.3518148051546681 0.4353168343792966
0.4436241578922681 0.4026098691092556 0.4542301356967047
0.5507999427707522 0.5781880182134552 0.4102630380980676
0.3085603912838142 0.3638207577309149 0.6318744156300781
0.4331749797878197 0.5829848649646556 0.5399946825583740
0.5681841845808776 0.6404826017330514 0.6422703106500635
0.4095106463285489 0.4928025652860386 0.7001081006613702
0.5195235459104173 0.4625507052505664 0.3676358806152366
0.3379264202350854 0.4076247878105236 0.3709855970792540
0.4219715177420865 0.6128753268690788 0.4131920814882156
0.2411977499443065 0.3044217003965644 0.5454617945450997
0.4084464093000061 0.4518702526618948 0.5684902024461332
0.5815123407435129 0.6140388671555269 0.5254366504312600
0.3359927063914029 0.4059559010654137 0.7458298909342473
0.4532015154123830 0.6098230875413779 0.6729787361846170

MgO_cube D
1.00000000000000
10.0000000000000000 0.0000000000000000 0.0000000000000000
0.0000000000000000 11.0000000000000000 0.0000000000000000
0.0000000000000000 0.0000000000000000 12.0000000000000000
Mg O
4 4
Direct
0.4082538028589898 0.5416524900406519 0.5305878117983606
0.5936034756190632 0.3731278114071152 0.5305767780108553
0.4082398469110048 0.3731386945814118 0.6850268543831768
0.5936000934735139 0.5416608289468938 0.6850294361068335
0.3977344214197258 0.3635674873538924 0.5217892621768641
0.6041438268024253 0.5512188136042010 0.5217897606980432
0.3977338878058405 0.5512244411032069 0.6938169507073241
0.6041304931094267 0.3635694489626138 0.6938230181185242

```
MgO_cluster E
   1.00000000000000
    10.0000000000000000    0.0000000000000000    0.0000000000000000
     0.0000000000000000   11.0000000000000000    0.0000000000000000
     0.0000000000000000    0.0000000000000000   12.0000000000000000
   Mg   O
    18    18
Direct
  0.2959874158288423  0.2645106382890104  0.4134116768187027
  0.2972331911203911  0.4489726769670515  0.2488587429501073
  0.2959186808314357  0.6334457412974923  0.4134200109843912
  0.2882973057675872  0.4490457354419195  0.5835811049199888
  0.4989305485260425  0.2657041749174871  0.2489007588822746
  0.7017491640327241  0.2645757271042858  0.4134222216676766
  0.4988086933472610  0.2575363439943892  0.5836185997158274
  0.7004099490210128  0.4490348548135529  0.2488407619465835
  0.4987813232248385  0.6322633597429060  0.2488537663758037
  0.4988137175220473  0.4490588808685522  0.4165959097192011
  0.7017349983646537  0.6334672882385244  0.4133926630089443
  0.7094138354748839  0.4490458640522886  0.5835620583992270
  0.4988376991035101  0.6404174764249386  0.5835794466621222
  0.3064328935928980  0.2739533547973401  0.7448548206597559
  0.3064310608198497  0.6239637688137293  0.7448932758941146
  0.6913824277672619  0.2739795195999774  0.7448428170977751
  0.4988472972819903  0.4490177460609164  0.7611264432338903
  0.6914101194812377  0.6239358528325167  0.7448573800629448
  0.2993911574490689  0.2673772511624928  0.2503387203246407
  0.2881861531327853  0.2575897351271050  0.5862363593375814
  0.2992589656348493  0.6305073316527244  0.2503594029614759
  0.2841547850784858  0.4489851905914294  0.4211439005905770
  0.2881820146986552  0.6403655831629522  0.5862970759239642
  0.6984738763850771  0.2674255962055356  0.2503540669142035
  0.4988696103682354  0.2537110887899192  0.4212005945174147
  0.7096166605348928  0.2576359794868146  0.5862281105981357
  0.4988405898086558  0.4489890583508828  0.2434184850673640
  0.6983495293238030  0.6305723151605439  0.2503363157005854
  0.4988243507162149  0.6441094977148760  0.4212147954178089
  0.7134894469277889  0.4490439623924248  0.4211124427541937
  0.4988500700818514  0.4489909629894919  0.5899013301223338
  0.7095737804808089  0.6403727978004691  0.5862509328549992
  0.2897772876862178  0.4490276417715057  0.7613903791575024
  0.4988807401077252  0.2590852527294311  0.7612882479983027
  0.4989013966873119  0.6388756205864120  0.7613363735921150
  0.7080391317891085  0.4490460340680894  0.7613803221674109
```

```
MgO_sheet F
   1.00000000000000
    10.0000000000000000    0.0000000000000000    0.0000000000000000
     0.0000000000000000   11.0000000000000000    0.0000000000000000
     0.0000000000000000    0.0000000000000000   12.0000000000000000
   Mg   O
     8     8
Direct
  0.2122428951233519  0.4888808632004267  0.2355775269512616
  0.2011887644211326  0.4888995825197568  0.5522619137001504
  0.5994120009727038  0.4889027985610012  0.2237733917275332
  0.3983346053740744  0.4888799965780747  0.3864555028112145
  0.5941231300436280  0.4888900127357631  0.5479256359129430
  0.7913143132479618  0.4888918982277874  0.3820981342660234
  0.3931430103028833  0.4889283875707254  0.7105632741703871
  0.7801770349800232  0.4889194694430495  0.6987417556192352
  0.1917606519726378  0.4888534079627606  0.3893856911670981
  0.3990306698165340  0.4888639252495672  0.2165499925959427
  0.5929844287488036  0.4888854930705861  0.3867737722032379
  0.3994900933936249  0.4888763376778021  0.5475912258636036
  0.7912472226405702  0.4888276432008194  0.2236730605932723
  0.8006496342849005  0.4888593001831383  0.5449596855572647
  0.2013427615489372  0.4888525472836440  0.7106635850823771
  0.5934386191282209  0.4888684645351362  0.7178057237784607
```

```
MgO_sheet G
   1.00000000000000
    20.0000000000000000    0.0000000000000000    0.0000000000000000
     0.0000000000000000   20.0000000000000000    0.0000000000000000
     0.0000000000000000    0.0000000000000000   10.0000000000000000
   Mg   O
    12    12
Direct
  0.2979884765069120  0.6706504787119224  0.5142686014683810
  0.3897771507457637  0.5767118418685401  0.5135185600017419
  0.2897819728217525  0.4820295573329264  0.5158610477001339
  0.3873474237453228  0.3822190153117554  0.5151094722811771
  0.4866829339069325  0.6745040762748815  0.5112772974131535
  0.5843236230294760  0.5747478246305108  0.5105243845138161
  0.4899700782324652  0.4742894759701802  0.5126876814656472
  0.6012178869427363  0.3603707328429510  0.5118754426684933
  0.6736833683936381  0.6694910685829927  0.5085317345273436
  0.6829492721363870  0.4797940287117853  0.5097607463486369
  0.2905010916647172  0.2949980163699742  0.5172822450886401
  0.4798689952975219  0.2814241557737144  0.5144535237537546
  0.3908471467808802  0.6801153530292654  0.5127022117915361
  0.2864339620410613  0.5780088937667789  0.5152379836478235
```

0.3872496522456539 0.4798397085072898 0.5142603055079162
0.2805974113628091 0.3869851604250392 0.5167838196200742
0.5819389138179523 0.6814820429604364 0.5097606738479421
0.4866920277497031 0.5770419845643122 0.5119534129940656
0.5860267641974010 0.4780363058963000 0.5110920274767736
0.4840074660940040 0.3783582954456892 0.5136469248367868
0.6865300157162195 0.5783195895846498 0.5090703941920853
0.6939841319053962 0.3879324477841960 0.5103302134787159
0.3813193624800687 0.2801516660850558 0.5159932940265665
0.5714208091852292 0.2683083185688442 0.5130180173488014

MgO_trimer Cluster H
1.00000000000000
10.0000000000000000 0.0000000000000000 0.0000000000000000
0.0000000000000000 11.0000000000000000 0.0000000000000000
0.0000000000000000 0.0000000000000000 12.0000000000000000
Mg O
3 3
Direct
0.5355358481311138 0.3866423269080910 0.3354586992860368
0.5353038080383631 0.6151375678852745 0.4564731201697476
0.5357063010709784 0.3866175651125071 0.5783600605585972
0.5358217106273325 0.2841683989108562 0.4568874039361921
0.5351424896342168 0.5523378008279511 0.3139199127291889
0.5352498124979742 0.5524063823553232 0.5989607773202421

MgO_wire CLuster I
1.00000000000000
10.0000000000000000 0.0000000000000000 0.0000000000000000
0.0000000000000000 10.0000000000000000 0.0000000000000000
0.0000000000000000 0.0000000000000000 30.0000000000000000
Mg O
12 12
Direct
0.8116846123080911 0.3675594737911528 0.4224416604518191
0.4237547542167882 0.4673573428385360 0.4526777886138618
0.5684433149975280 0.2607997893653387 0.4904989621406318
0.7974554888232347 0.2774666751780261 0.5438951540975930
0.8496351238859551 0.4357506341226375 0.6182326361833506
0.6297254481665530 0.4629346959362290 0.6791511956959272
0.8878907796613661 0.7307356857035247 0.4254864699923599
0.7226836483171629 0.5103816081425873 0.4972424596730525
0.5211880100807175 0.3364641515189982 0.3840793199740182
0.6577329046051127 0.1945804724669587 0.6148184373564118
0.6583113611075063 0.5644235121318132 0.3866911407372076
0.5662060959078413 0.4267157904899895 0.5677356309581814
0.7043735934388313 0.3764681533372028 0.3707749165169791
0.7667486063618346 0.3037874877942655 0.4801919422192305
0.5265128989445905 0.4753746686847472 0.5062821214248431

0.5984887045256703	0.2287021607654232	0.5545198373757880
0.8412020981946117	0.2421245834153873	0.6059048674455451
0.8003382655928541	0.5197032058285757	0.6713302876432597
0.7916240801759732	0.5668301982394074	0.4380634856400880
0.7655081273047335	0.4762933399099735	0.5596931906478156
0.4517951891768029	0.2749866958707043	0.4399392722989819
0.5466573913028892	0.2960350256092961	0.6578731425355422
0.4725730031826333	0.5282828474349899	0.3947384045463511
0.5030165667207239	0.4223518854242375	0.6311777458311649

8. Coordinates of MgO Clusters – MLFF$_{cluster}$ optimized

```
MgO_cluster A
   1.00000000000000
    10.0000000000000000    0.0000000000000000    0.0000000000000000
     0.0000000000000000   10.0000000000000000    0.0000000000000000
     0.0000000000000000    0.0000000000000000   20.0000000000000000
   Mg   O
     8     8
Direct
  0.5045003784589797  0.4093627855964879  0.3947824555054379
  0.5654818119368108  0.6603147406731806  0.4070206176211901
  0.6546985820447875  0.4861958210418088  0.4999566081161724
  0.3780234400678379  0.5559327356204616  0.4886916769241088
  0.4627641327473893  0.3682657732192045  0.5869378096386580
  0.5304895869040258  0.6449156088694906  0.6003617410891748
  0.3526941682276711  0.5247489242147567  0.6820012092856631
  0.6034189866332367  0.4613614728821760  0.6922035745623975
  0.6769531055794286  0.5000528657254415  0.4024904025470815
  0.3947066781802681  0.5708534385452683  0.3909444512168230
  0.4793115372399970  0.3705519898126851  0.4894509615940002
  0.5523692781766237  0.6709950034410009  0.5040651713694981
  0.6472170111691450  0.4691153663893914  0.5973241393718740
  0.3469728835094250  0.5446923496066725  0.5851135198469894
  0.4426656694399196  0.3513368171050148  0.6844411186560765
  0.5118027576844622  0.6335842732569398  0.6981144026548334
```

```
MgO_cluster B
   1.00000000000000
    15.0000000000000000    0.0000000000000000    0.0000000000000000
     0.0000000000000000   15.0000000000000000    0.0000000000000000
     0.0000000000000000    0.0000000000000000   15.0000000000000000
   Mg   O
     9     9
Direct
  0.4514255474630716  0.3090918457902691  0.2526360805068179
  0.5834543685835674  0.4065311613957680  0.2879953331779986
  0.3392653484554520  0.2354945784315292  0.4627520256124585
  0.4480119026846405  0.4022292642559976  0.4066797093509610
  0.3448362280321446  0.3629722751866168  0.6185039112721097
  0.5714955910002774  0.4584086301042330  0.5294042923385962
  0.4377120123994511  0.5180545858077560  0.6361788897855740
  0.3664340673625758  0.5241772406208611  0.8046231106407191
  0.5715374544487333  0.5998840345516797  0.7308167618386532
  0.5716922192096517  0.3042603734332616  0.2147088627929881
  0.4555757204655327  0.4362872116698039  0.2815438656549296
  0.3961669392270718  0.2820495327093300  0.3651656949852741
  0.5870306765477068  0.4179607805481187  0.4141689128658678
  0.3029350187667291  0.2561683782767139  0.5754652532050080
```

0.4361264625963930 0.4289468588261445 0.5381184182442874
0.5698653093969857 0.5389712268512794 0.6247075705814296
0.3440755927418791 0.4547859207632009 0.7059391455970200
0.4611098196181231 0.5951263047774230 0.7795922535492859

MgO_cluster C
1.00000000000000
15.0000000000000000 0.0000000000000000 0.0000000000000000
0.0000000000000000 15.0000000000000000 0.0000000000000000
0.0000000000000000 0.0000000000000000 15.0000000000000000
Mg O
8 8
Direct
0.4063905632401418 0.5109058896751572 0.3429937328203406
0.2527087853558849 0.3559525420603531 0.4376433254246029
0.4539191276246591 0.3947574692699196 0.4540306863199428
0.5505743671788279 0.5909573149458125 0.4243564681594074
0.2949604268715908 0.3660860693810939 0.6337155550761978
0.3987137053212075 0.5632548730008350 0.5422602683712664
0.5629850029052547 0.6493059403647043 0.6170990530464571
0.4197564867854126 0.4783197841081788 0.7000740530773709
0.5264699044930247 0.4714761135104905 0.3814726776271435
0.3481135852986919 0.4015849116339347 0.3748854713975762
0.4172712123121358 0.6086901733224769 0.4247671138494375
0.2166234604057086 0.3239563021790394 0.5477937252023322
0.4042471571606009 0.4327586643324753 0.5685147937028013
0.6174361160420403 0.6510889741842282 0.5096490260296129
0.3333547545205867 0.4047457775938890 0.7457943615697826
0.4596155224842092 0.5910494254374087 0.6556998273257028

MgO_cube1   Cluster D
1.00000000000000
10.0000000000000000 0.0000000000000000 0.0000000000000000
0.0000000000000000 11.0000000000000000 0.0000000000000000
0.0000000000000000 0.0000000000000000 12.0000000000000000
Mg O
4 4
Direct
0.4093408145605323 0.5406646828525901 0.5314786010627283
0.5925266091620331 0.3741237490897989 0.5314831151197084
0.4093321527125528 0.3741265006666694 0.6841296981001062
0.5925272291083389 0.5406632218540558 0.6841285281940499
0.3962304897097794 0.3622108560944515 0.5205461076745445
0.6056336768748292 0.5525723649257669 0.5205491001588205
0.3962280664016440 0.5525828035902476 0.6950628858848937
0.6056208094702732 0.3622158369264140 0.6950618358051232

```
MgO_cube2          Cluster E
   1.00000000000000
    10.0000000000000000    0.0000000000000000    0.0000000000000000
     0.0000000000000000   11.0000000000000000    0.0000000000000000
     0.0000000000000000    0.0000000000000000   12.0000000000000000
   Mg   O
    18    18
Direct
  0.2955261149125240  0.2640958216144378  0.4180146692381270
  0.2978473809803169  0.4489444282898493  0.2560312410554924
  0.2954715460662072  0.6338451457758461  0.4180873737419842
  0.2875691557250922  0.4489784298716202  0.5827507623852565
  0.4988649936211200  0.2662652986575185  0.2560158672943430
  0.7022269959707854  0.2641332418235439  0.4179817335865825
  0.4988876978353045  0.2568826576973010  0.5827076721085349
  0.6998380843824251  0.4489883642457652  0.2560087556855210
  0.4988264584877363  0.6317087523655762  0.2560138187942231
  0.4988618948359754  0.4489805527829180  0.4194355773555012
  0.7022006784111869  0.6338828083559428  0.4180535828657008
  0.7101747517902628  0.4490135060566731  0.5827169380219231
  0.4988507614978894  0.6410964729990123  0.5827608854734881
  0.3053762354413507  0.2730764178971772  0.7413895633963790
  0.3053753264329396  0.6249047216102949  0.7414193111941867
  0.6924166488995880  0.2731001243458935  0.7413637750961508
  0.4988806044354976  0.4490161668268968  0.7536789625715814
  0.6923547164023330  0.6249384887391974  0.7413916111349230
  0.3017562776002995  0.2697993696410976  0.2560917596380028
  0.2904785685112204  0.2594968816919926  0.5826266035477641
  0.3017203287226496  0.6281560730224898  0.2560708515152957
  0.2750548585840237  0.4489786135430212  0.4219889567068484
  0.2904303444011261  0.6384512340045810  0.5825306361707406
  0.6959670806855976  0.2698390497030671  0.2560692319257368
  0.4988784703263116  0.2455411559039401  0.4219934082225517
  0.7072993290862686  0.2595298895766393  0.5826082834392328
  0.4988390698773736  0.4490042374118520  0.2404536682451018
  0.6959184081243007  0.6281963435991341  0.2560524889459414
  0.4988323296576005  0.6524447088264541  0.4219582794013969
  0.7226546326178479  0.4490182083875313  0.4219659081860757
  0.4988744853112381  0.4489825841335774  0.5814897523999693
  0.7072718436498820  0.6384853174030377  0.5825215926452852
  0.2911611771196444  0.4489311426358915  0.7550510709099582
  0.4989103355321266  0.2601369174126051  0.7550424725970083
  0.4988719849210532  0.6378388130776224  0.7550380507160309
  0.7066102971429109  0.4489579640699872  0.7550251987870935
```

MgO_sheet1 Cluster F
1.00000000000000
10.0000000000000000 0.0000000000000000 0.0000000000000000
0.0000000000000000 11.0000000000000000 0.0000000000000000
0.0000000000000000 0.0000000000000000 12.0000000000000000
Mg O
8 8
Direct
0.2128507068769842 0.4888723069409686 0.2344174053895894
0.1947451533100620 0.4888741043173747 0.5517655877049011
0.5967162370873357 0.4888725803328052 0.2225721223781668
0.3964203949878294 0.4888773093609627 0.3852225904117645
0.5960668580933304 0.4888767282300489 0.5491251244689851
0.7977469487855893 0.4888709896007356 0.3825888024237352
0.3957694312117798 0.4888910362936288 0.7117695846141473
0.7796252853732483 0.4888993949230031 0.6999361819170705
0.1860308395675154 0.4888666341653631 0.3874588656286572
0.3982062515108534 0.4888699870997460 0.2148951918536185
0.5946826164012871 0.4889175854757624 0.3872891160736094
0.3978053978448823 0.4888897636219808 0.5470589705641771
0.7884624918460437 0.4888503165832162 0.2235643711305464
0.8064530813358437 0.4888724580434143 0.5468963178825369
0.2040255198142812 0.4888822956198614 0.7107890525226142
0.5942726219531226 0.4888966373911597 0.7194505870358999

MgO_sheet2 Cluster G
1.00000000000000
20.0000000000000000 0.0000000000000000 0.0000000000000000
0.0000000000000000 20.0000000000000000 0.0000000000000000
0.0000000000000000 0.0000000000000000 10.0000000000000000
Mg O
12 12
Direct
0.3000651285962532 0.6685154606218036 0.5142700365003210
0.3899226106972261 0.5765799784583250 0.5135282430086858
0.2885119226259497 0.4822698868161924 0.5158846772213757
0.3872352316824805 0.3830944760253434 0.5151292437186320
0.4865325825625009 0.6758039488681259 0.5112526284080652
0.5834287951773033 0.5748239348063665 0.5105216597109431
0.4888820484639338 0.4753386284177733 0.5126752766095481
0.6029692595323870 0.3585692676797341 0.5118743889241040
0.6724577976620456 0.6690328373724889 0.5085667689767476
0.6834106766278534 0.4813407696117215 0.5097513650372328
0.2909902823968397 0.2962527216068742 0.5172787163453287
0.4783771798055653 0.2810030692551754 0.5144511356789297
0.3911112987228333 0.6806753324898412 0.5127007646461823
0.2858289156204020 0.5777710293494944 0.5152369706596767
0.3870720639028860 0.4800404319598925 0.5141780025057840
0.2815935857702705 0.3874248701538791 0.5167690052049345

0.5815226156961119 0.6805152565924284 0.5097804971925786
0.4865035664673628 0.5772192095963989 0.5120088489589086
0.5855892683823285 0.4769200132719007 0.5109915514162289
0.4850688830856205 0.3786940801878963 0.5137429745777796
0.6867719615850555 0.5784377939789664 0.5090672102061039
0.6961891378944751 0.3895150122897689 0.5103198506910339
0.3812337594890852 0.2798835463966904 0.5159774677271511
0.5698713645532345 0.2660884831929097 0.5130427320737226

MgO_trimer Cluster H
1.00000000000000
10.0000000000000000 0.0000000000000000 0.0000000000000000
0.0000000000000000 11.0000000000000000 0.0000000000000000
0.0000000000000000 0.0000000000000000 12.0000000000000000
Mg O
3 3
Direct
0.5355172722376492 0.3849354743134510 0.3331130849823245
0.5352182891372509 0.6183553520632907 0.4564685489323407
0.5356720053787309 0.3850476254346423 0.5805327359956239
0.5358315939539238 0.2870046885729654 0.4568822733701803
0.5351997944189048 0.5509030021114160 0.3165533295010164
0.5353210148735195 0.5510638995042381 0.5965100012185189

MgO_wire Cluster I
1.00000000000000
10.0000000000000000 0.0000000000000000 0.0000000000000000
0.0000000000000000 10.0000000000000000 0.0000000000000000
0.0000000000000000 0.0000000000000000 30.0000000000000000
Mg O
12 12
Direct
0.8055363154780667 0.3791184070777586 0.4221345784063997
0.4182664293901814 0.4626096603334807 0.4524976927313761
0.5763155246652474 0.2592917041487190 0.4888367994755448
0.7994459502335681 0.2905448378844822 0.5426415781762085
0.8298068361216812 0.4525037365761116 0.6171929343296176
0.6305679743008439 0.4572812054621073 0.6827458532595179
0.9464988720843297 0.6484704649078080 0.4359925020981212
0.7147426908177447 0.5205341471671706 0.4967960825739735
0.5185777445204504 0.3369645450318293 0.3822582635111677
0.6547519450566662 0.2067611198254276 0.6122896278818297
0.6442071793365381 0.5679820146397888 0.3882276902723466
0.5619882312512526 0.4280190284145650 0.5652299003068096
0.6998678087693165 0.3878266326809661 0.3701116989016924
0.7699649554580171 0.3148985182838621 0.4798068921718525
0.5220283969090577 0.4771405209137359 0.5051568914573280
0.6054816939002394 0.2309790301119049 0.5514089257440976
0.8353839051688702 0.2623949239924306 0.6046483384467568

0.7912903876567805	0.5289124733668668	0.6721689290755434
0.7685018845033834	0.5731340363866155	0.4374808625608403
0.7552623050755278	0.4875482709091167	0.5583423972437639
0.4594111085881607	0.2733611323771132	0.4388297486440873
0.5657890758680616	0.2800258876841936	0.6639674573045138
0.4600868146045089	0.5255236477735286	0.3940549311065023
0.5297760372415269	0.3942841380504038	0.6306194943201056

9. Coordinates of FeO_x Clusters – PBE optimized

```
FeOx_cluster A
   1.00000000000000
    15.0000000000000000    0.0000000000000000    0.0000000000000000
     0.0000000000000000   15.0000000000000000    0.0000000000000000
     0.0000000000000000    0.0000000000000000   15.0000000000000000
   Fe   O
    10    12
Direct
  0.7277029376735200  0.5786179876111959  0.6502625651935858
  0.6901193445062219  0.4292298643758912  0.7079071267250470
  0.3724886534299827  0.5861965162103218  0.6473977494259842
  0.3689142559907097  0.4307219970021450  0.5928691332650189
  0.3321789336013055  0.4482425405968513  0.7572551560631879
  0.4450547541388943  0.5679112827854098  0.8078240283582190
  0.5596018346987250  0.4276754724442711  0.7879711481846101
  0.5390858212380678  0.5445710702853788  0.6073900610908325
  0.2829178454158594  0.5967772059504171  0.7866842153842910
  0.6777873504503091  0.5435737103381797  0.8206094082403155
  0.6727367513102465  0.4164730875077964  0.8330982208932483
  0.7154152530928855  0.6372423459777750  0.7591160374389077
  0.7786148964914901  0.4640428834076076  0.6419190292304862
  0.5763034759112244  0.4287063046027768  0.6651601468070396
  0.6412518999725023  0.5915738757216289  0.5711758695254332
  0.2288566406292034  0.4972300302845269  0.7969721759902839
  0.4417291624864603  0.4357579529901372  0.8160711663369398
  0.2752705168232481  0.6524917078060071  0.6879078888823500
  0.3541883706041133  0.6351210427852081  0.8662689826092063
  0.4302761515050904  0.5230418296209436  0.5439086989317445
  0.3168222229731015  0.3622492533512016  0.6739964393646645
  0.4900029530568375  0.6119519953443486  0.7006748740585920
```

```
FeOx_cluster B
   1.00000000000000
    15.0000000000000000    0.0000000000000000    0.0000000000000000
     0.0000000000000000   15.0000000000000000    0.0000000000000000
     0.0000000000000000    0.0000000000000000   15.0000000000000000
   Fe   O
    6     7
Direct
  0.4897245312126870  0.3377709212188833  0.6629994730369120
  0.3757954506069723  0.4572217015339533  0.7028804026191651
  0.5020032234779563  0.3278293163886801  0.4816490252048382
  0.4112295178208001  0.4725687279556752  0.5455928527245675
  0.6215868060452294  0.4338377269290632  0.4336185607354039
  0.5760470846690993  0.4611861334805099  0.5906896599015354
  0.4888917774931585  0.2605877959500020  0.5782575411653781
  0.3787067829722801  0.5520827225492511  0.6314717609454519
```

0.4112453325709637 0.3501130217446615 0.7471176267217956
0.5930961799239114 0.3850603787281770 0.6836570284415160
0.5867834235181206 0.3247647973554706 0.3982864096177536
0.4013572074043950 0.3847835667232644 0.4626130358964957
0.6151926302844331 0.5320231674424036 0.5001566379892068

FeOx_cluster C
1.00000000000000
15.0000000000000000 0.0000000000000000 0.0000000000000000
0.0000000000000000 15.0000000000000000 0.0000000000000000
0.0000000000000000 0.0000000000000000 15.0000000000000000
Fe O
11 6
Direct
0.4026999785084939 0.5672904348697188 0.3832024355788164
0.5913100966837206 0.4305630579005211 0.5246779091751961
0.4340076221931923 0.4210615830255762 0.2980708904556195
0.5457741961541913 0.5011196312149622 0.3873226591485732
0.4285646016557720 0.4199498145731724 0.4679550716176735
0.6005135142616425 0.4318572085187142 0.2530269233612614
0.6891476996272345 0.4352004373115221 0.3921677832957968
0.5002904853991410 0.5721665093952168 0.2502372728403870
0.6574290104949014 0.5816458509711353 0.4762443815195212
0.4917407680897483 0.5703257741294294 0.5222607946893305
0.6631591560086036 0.5823675338950736 0.3064632044961400
0.4982895430900598 0.3581429733674426 0.5426214115543573
0.3450013902561295 0.4587644138765725 0.3804873587725695
0.5086379205327063 0.3602870581059534 0.2274028714603773
0.7465852197697416 0.5439297792026494 0.3941152901220946
0.5937220004377224 0.6439664392111624 0.2313638148232542
0.5834068198370019 0.6424115654311819 0.5476498540890233

FeOx_cluster D
1.00000000000000
15.0000000000000000 0.0000000000000000 0.0000000000000000
0.0000000000000000 15.0000000000000000 0.0000000000000000
0.0000000000000000 0.0000000000000000 15.0000000000000000
Fe O
8 12
Direct
0.3352130472688017 0.4067879377333341 0.4580018476490295
0.5753309443415020 0.3850643909385738 0.4740842987784788
0.4629009808339504 0.3781447813508620 0.3285050047812135
0.6306144148213590 0.3225189414261322 0.3386150077419714
0.4596701857165471 0.6114481535875669 0.3613905313810477
0.3200434866319313 0.5934203511080938 0.4995587688508749
0.5707335614545528 0.5674634248907537 0.4940975969269559
0.3315738559982719 0.5019423236266647 0.3279930371338295
0.5445386910738890 0.3196079917079970 0.2574634489541623
0.6803693788490293 0.3374021086687193 0.4470872379384829

0.5808727941560861 0.4727352690069395 0.5552413651459034
0.4554250519293106 0.3671295809795367 0.4609067649869445
0.6590690197371956 0.6187465047242853 0.4674801271517239
0.5029351190454534 0.6921352354830361 0.3098351907874957
0.2760414695165920 0.6721951454693667 0.5552994518861354
0.3188215834704877 0.4871803458413808 0.5450667511298022
0.3245339713525794 0.3789003899689689 0.3334052931873472
0.3335912251319755 0.6154994218423084 0.3792761287130304
0.4661794778486410 0.6174608210930330 0.4811475839544386
0.4637418008218646 0.5064268945524617 0.3073844929211305

FeOx_cluster E
1.00000000000000
15.0000000000000000 0.0000000000000000 0.0000000000000000
0.0000000000000000 15.0000000000000000 0.0000000000000000
0.0000000000000000 0.0000000000000000 15.0000000000000000
Fe O
6 10
Direct
0.5882150565643300 0.6369161843076583 0.4286418459253127
0.4636292473639116 0.4930179105288062 0.4275612278107630
0.6436205432866231 0.4901810266242634 0.5252569083006335
0.4746135119362904 0.3633965161260164 0.3109132813888493
0.4890099441411238 0.6122828790860311 0.2892105537037963
0.5582982466327309 0.3656481807771570 0.4626333742173188
0.4709680865111778 0.6158716485584321 0.4347759854234994
0.4237716458065819 0.6739597360192349 0.2315039125039107
0.5969384568505518 0.6548061653288784 0.3128918218032055
0.6687466152939763 0.6082224378037537 0.5074223287487740
0.5774034428278796 0.3252991140116706 0.3488827598059601
0.4143783280118072 0.3035780332070317 0.2482642973745257
0.4336016115692090 0.3716451006888875 0.4264241040742860
0.6663713214109421 0.3709309999869888 0.5103676585440624
0.4736805992610726 0.4919758392348115 0.3010260102898954
0.5181833875317849 0.4626282437103768 0.5361038110852263

FeOx_cluster F
1.00000000000000
15.0000000000000000 0.0000000000000000 0.0000000000000000
0.0000000000000000 15.0000000000000000 0.0000000000000000
0.0000000000000000 0.0000000000000000 15.0000000000000000
Fe O
12 8
Direct
0.4675159571421830 0.5952602386275530 0.4920830344301521
0.4398143618110311 0.3839877351523597 0.6576239880047723
0.4524840654846756 0.3284179185037246 0.4888574748315335
0.4487087465257247 0.5372662222742335 0.6589922385948199
0.5963113094184678 0.4521575945890248 0.6678830674600889
0.3420723106349399 0.4681017800370526 0.5482681154003544

0.5793032340403741 0.3188363542451143 0.5824668699912792
0.5970246152971725 0.5339747827553083 0.4183782890348482
0.6869653305003922 0.4478866748376618 0.5337981705404857
0.5891835239135901 0.3784805222367567 0.4187822493759583
0.5941855907636935 0.5884597221747043 0.5854450153145550
0.4382019727318394 0.4638567060067231 0.3983258192888357
0.5013585350751981 0.4562697939013702 0.7455058915754051
0.3401536888325865 0.4665149636415578 0.6726162585690645
0.3720630131093543 0.5913035824444393 0.5689765476143691
0.3580560311270489 0.3417636622520490 0.5658509835404146
0.3230228942299718 0.4710764579783202 0.4284009815003884
0.6734180640539691 0.3289922898118007 0.5021881181533274
0.5449087845735292 0.4590028720082984 0.3326722113894647
0.6854380197342564 0.5680901115219470 0.5037046973898782

FeOx_cluster G
1.00000000000000
15.0000000000000000 0.0000000000000000 0.0000000000000000
0.0000000000000000 15.0000000000000000 0.0000000000000000
0.0000000000000000 0.0000000000000000 15.0000000000000000
Fe O
6 7
Direct
0.4991579169198843 0.3832050658835655 0.3135030085785786
0.5035117880706892 0.5888401873896996 0.3431364654241236
0.7052147368897863 0.4188520762474468 0.2842431203486129
0.7060099266386501 0.5768008924118675 0.2992191359029590
0.2975514639341483 0.4269622994073288 0.2739377340506053
0.3032839514454722 0.5851513365502470 0.2870890649546993
0.6098798855465049 0.3481676909617022 0.2910855601491562
0.3880565806530427 0.3512529099031951 0.2885036821919513
0.4999910312660703 0.4752935889062186 0.3815001072580415
0.6095701220597718 0.6379022748571757 0.3213710046415557
0.7950546530403173 0.4969544552297052 0.2901424861822690
0.4008838322328785 0.6419351977182401 0.3148597451119670
0.2115741473027839 0.5094419805336159 0.2714389032054783

eOx_cluster H
1.00000000000000
15.0000000000000000 0.0000000000000000 0.0000000000000000
0.0000000000000000 15.0000000000000000 0.0000000000000000
0.0000000000000000 0.0000000000000000 15.0000000000000000
Fe O
2 5
Direct
0.4542450273700354 0.3262383722848554 0.3346866933022099
0.4520327975482772 0.5143832347006096 0.3227038769803414
0.3601329044882533 0.5515243349698622 0.2892215489442833
0.5456881203110768 0.5553131649120147 0.3004436561308168
0.5487495715822917 0.2842010160384305 0.3191989407905496

0.3633186588502397 0.2829530032941108 0.3064999767610033
0.4494428928498166 0.4245068688001155 0.3944353460907948

FeOx_cluster I
1.00000000000000
15.0000000000000000 0.0000000000000000 0.0000000000000000
0.0000000000000000 15.0000000000000000 0.0000000000000000
0.0000000000000000 0.0000000000000000 15.0000000000000000
Fe O
5 6
Direct
0.5441335776614977 0.5308043647126794 0.2611616282580442
0.5553934498496744 0.6514239845042269 0.3580735720927137
0.4921751050198608 0.4999006839175593 0.4521927434029465
0.6469025545090474 0.5326927775717749 0.4057698446231157
0.5662410996435696 0.6042787276428569 0.5342648195700477
0.5126405655855990 0.5055962027247460 0.5742420588359352
0.4778437255863750 0.6334416495315836 0.2669781865305296
0.6481222386422019 0.6514974361248207 0.4543677464606191
0.4736958183146172 0.6364754957235590 0.4510086285316888
0.6512912922923420 0.5975623424386569 0.2936246718163034
0.5567306548952331 0.4455763801075321 0.3562160748780769

10. Coordinates of FeO_x Clusters – MLFF optimized

```
FeOx_cluster A
   1.00000000000000
    15.0000000000000000    0.0000000000000000    0.0000000000000000
     0.0000000000000000   15.0000000000000000    0.0000000000000000
     0.0000000000000000    0.0000000000000000   15.0000000000000000
   Fe   O
    10    12
Direct
  0.7179812594803713  0.5725651012312274  0.6588146398518908
  0.6699282668704958  0.4301371706354277  0.6913740403244774
  0.3883420056893780  0.6013829311769899  0.6452221978893664
  0.4270946013132177  0.4312426588524412  0.6290677656185039
  0.3393094173787081  0.4336401408866313  0.7766587220280309
  0.4446079338839053  0.5692179077366335  0.7939662941317616
  0.5254012916049419  0.4280335000174099  0.7692971013725953
  0.5537608942918257  0.5486184300518558  0.6363624431355482
  0.2822312517752588  0.5909430019612769  0.7735901859173244
  0.6571158900207693  0.5352161471037934  0.8012300249316976
  0.6445329278834823  0.4135015562168787  0.8146396844510538
  0.7206755646382574  0.6261291686829327  0.7627743564061368
  0.7670586016555031  0.4697149179556662  0.6387249595714979
  0.5525438258287937  0.4102978291514044  0.6407752204486916
  0.6425915221685844  0.6048498957633520  0.5790217911880968
  0.2367109072844640  0.4883235561870315  0.8027258298308798
  0.4374779978562628  0.4521295419175977  0.8511911193223840
  0.2766445184221581  0.6371197937826274  0.6674237644766140
  0.3587106499449002  0.6355055191263091  0.8460912243286434
  0.4317538522133938  0.5291331320703252  0.5572783283149972
  0.3507400466706400  0.3604020095248907  0.6839541991746916
  0.4921067991246890  0.6412960469673183  0.7022562292851007
```

```
FeOx_cluster B
   1.00000000000000
    15.0000000000000000    0.0000000000000000    0.0000000000000000
     0.0000000000000000   15.0000000000000000    0.0000000000000000
     0.0000000000000000    0.0000000000000000   15.0000000000000000
   Fe   O
     6     7
Direct
  0.5084210583361718  0.3222718164268460  0.6603412829975328
  0.4415243187190900  0.4714909040492227  0.6583816431950127
  0.4784375659429186  0.3153166972764935  0.4889490774661732
  0.4146962498437315  0.4658006254980629  0.4996641314108814
  0.5547991339379973  0.4596371571152728  0.4771867038111073
  0.5819569257123463  0.4662782899743053  0.6364338283793908
  0.4911988440291919  0.2383730204773586  0.5783326873101907
  0.3927367638880416  0.5481667531812073  0.5820908521652320
  0.4199946076118218  0.3722527034512031  0.7194226639641846
```

```
 0.6141449379040678  0.3650085880655705  0.6888181494332526
 0.5689068705566271  0.3542118046750758  0.4249501066781807
 0.3753061770506825  0.3623247021420369  0.4576476901578367
 0.6095364944673191  0.5386969156673397  0.5467711980310426


FeOx_cluster C
   1.00000000000000
    15.0000000000000000    0.0000000000000000    0.0000000000000000
     0.0000000000000000   15.0000000000000000    0.0000000000000000
     0.0000000000000000    0.0000000000000000   15.0000000000000000
   Fe   O
    11     6
Direct
 0.3978843624631642  0.5478409863419095  0.3838634583289340
 0.5848772823670692  0.4391420629546717  0.5000849382734758
 0.4408390595641681  0.4359722357030392  0.3024167716690641
 0.5453882028476653  0.5011489168426219  0.3874125915525591
 0.4359263305822514  0.4348664523757588  0.4640368660920598
 0.5922372870205288  0.4400631794754640  0.2765400961286402
 0.6940946642431918  0.4544600476748293  0.3930862494242266
 0.5069188749295429  0.5639918519828413  0.2743111207396751
 0.6506465452630432  0.5666210790812990  0.4719204298136819
 0.4995448733528780  0.5624218431374577  0.4980257102546765
 0.6562580693502638  0.5673617128210697  0.3101257463026307
 0.4993348469050913  0.3915198314476062  0.5485200274866865
 0.3437919417294445  0.4449520880623172  0.3805732481155776
 0.5097517592337704  0.3934163726431343  0.2217352282871332
 0.7480444348986510  0.5576609099091754  0.3945606862128213
 0.5928831417591289  0.6107663541711812  0.2253198261521058
 0.5818583464901506  0.6088441403756271  0.5527369321660442


FeOx_cluster D
   1.00000000000000
    15.0000000000000000    0.0000000000000000    0.0000000000000000
     0.0000000000000000   15.0000000000000000    0.0000000000000000
     0.0000000000000000    0.0000000000000000   15.0000000000000000
   Fe   O
     8    12
Direct
 0.3410165380595815  0.4034871073926387  0.4593313052690163
 0.5733399561662498  0.3726842486314756  0.4685441968581458
 0.4990791542843254  0.3834723857409003  0.3362490924763510
 0.6399248160313584  0.3207212131042346  0.3372681065049086
 0.4485135412098336  0.6120048790702890  0.3515836342574481
 0.3385233268245780  0.6006393545945616  0.5006572801212036
 0.5768624829457348  0.5881662702459582  0.5104499166177220
 0.3327124204786522  0.4925955650274437  0.3244323514631992
 0.5594153804455960  0.3245203330168946  0.2538523690870551
 0.6564291496839497  0.2917826974142765  0.4480712783136136
```

```
  0.5999054158046954  0.4772393592426549  0.5160318685321404
  0.4504579589472530  0.3521070380550020  0.4498747129082872
  0.6463768185663917  0.6635132860808608  0.5325846624583663
  0.4897224633636252  0.6893076501605918  0.2925258505025521
  0.2878672266203464  0.6767006435687530  0.5540995832041546
  0.3258719155367160  0.4896191402719706  0.5352318735726298
  0.2768979531477302  0.3955992719873395  0.3604827005235467
  0.3245135601227033  0.6048911733697671  0.3743807851804272
  0.4681593708157224  0.6155168090268495  0.4747815103976108
  0.4566106109449792  0.4976415879975548  0.3014068517516203
```

```
FeOx_cluster E
   1.00000000000000
    15.0000000000000000    0.0000000000000000    0.0000000000000000
     0.0000000000000000   15.0000000000000000    0.0000000000000000
     0.0000000000000000    0.0000000000000000   15.0000000000000000
   Fe   O
     6    10
Direct
  0.5873503713183214  0.5989883323646374  0.4245663124982730
  0.4780202426746568  0.4952952173209823  0.4852593848079091
  0.6225338844348897  0.4905083136469156  0.5455409429111702
  0.4710416302265150  0.3817641915690803  0.2956852345917224
  0.4798004056310866  0.5965459595297022  0.2888923935965345
  0.5777915368568002  0.3784124740886615  0.4315645432141766
  0.4622247390327489  0.5973874373696185  0.4188755012572287
  0.4329208782639845  0.6752140318542071  0.2365214719463339
  0.5973966211712840  0.6008323324467183  0.3073324365825657
  0.6687566445316152  0.5912010548683223  0.5088963263948874
  0.5878740191114550  0.3693705626715391  0.3145148712533136
  0.4175295017681073  0.3041068845846792  0.2483931983699079
  0.4532790354726802  0.3910217386229622  0.4256019701701179
  0.6593239698111968  0.3836311778480385  0.5158812304949048
  0.4476614896467157  0.4892436688854407  0.2593813523402460
  0.5179250750479354  0.4968366383284945  0.5949727105707262
```

```
FeOx_cluster F
   1.00000000000000
    15.0000000000000000    0.0000000000000000    0.0000000000000000
     0.0000000000000000   15.0000000000000000    0.0000000000000000
     0.0000000000000000    0.0000000000000000   15.0000000000000000
   Fe   O
    12     8
Direct
  0.4610458274740801  0.5643454579361469  0.5076108641574197
  0.4250211853856933  0.3890870019343039  0.6565702966600269
  0.4525478395202485  0.3641784640333680  0.5061355276271533
  0.4325104188891230  0.5346775691964715  0.6585260162049981
```

0.5688463496484260 0.4532871242800164 0.6575913901546341
0.3517323596042246 0.4676802601789948 0.5291501039094156
0.5667128733427446 0.3362676633744904 0.5756131503028967
0.5811505547791304 0.5270529215171693 0.4290220652951802
0.6579702663195639 0.4492932084190561 0.5333151232097585
0.5751115348673822 0.3834003008166716 0.4261404883789494
0.5785974385717709 0.5719748351069809 0.5783757468455121
0.4313001294292316 0.4647692983115116 0.3798963078229734
0.4904749434531137 0.4579243131337724 0.7443951513339316
0.3248795346349422 0.4673263024043190 0.6492350937113296
0.4742353903630053 0.6365394373386076 0.6012665481533993
0.4548241858121565 0.2857530018345315 0.5954958053631617
0.3220945172804485 0.4694829957994952 0.4141915097414015
0.6604521904660701 0.3323190527674825 0.4972928454538998
0.5479927295628608 0.4581335062130800 0.3304878985656718
0.6726897795957805 0.5662072704035308 0.5005080891082820

FeOx_cluster G
1.00000000000000
15.0000000000000000 0.0000000000000000 0.0000000000000000
0.0000000000000000 15.0000000000000000 0.0000000000000000
0.0000000000000000 0.0000000000000000 15.0000000000000000
Fe O
6 7
Direct
0.4995516604675342 0.4015371207011213 0.3217069509499213
0.5032493998281655 0.5815431270354786 0.3429651228961534
0.6930126613417893 0.4110143181273065 0.2810308037268153
0.6974000172970487 0.5781778384723887 0.2997843091294342
0.3092778531722704 0.4186073396663099 0.2711708854062648
0.3120484934479495 0.5860481855336578 0.2881192022707793
0.5964879693653323 0.3480317849620366 0.2788746830392301
0.4023433483179309 0.3507750176528996 0.2763551179923224
0.4994279606333529 0.4827156346204401 0.4093201493992911
0.6021530550935731 0.6408799969026386 0.3152113706941394
0.7771465467937553 0.4924293119967618 0.2917367391552903
0.4087660146658244 0.6446810523327390 0.3093948535507009
0.2288750555754728 0.5043192279962290 0.2743598297896558

FeOx_cluster H
1.00000000000000
15.0000000000000000 0.0000000000000000 0.0000000000000000
0.0000000000000000 15.0000000000000000 0.0000000000000000
0.0000000000000000 0.0000000000000000 15.0000000000000000
Fe O
2 5
Direct
0.4541516431865161 0.3273553192052924 0.3361393491494700
0.4519725098173963 0.5132072129450840 0.3238361614925012

0.3614266853326867 0.5524553648562956 0.2879820571727590
0.5445028174268111 0.5561816913693953 0.2990616692231232
0.5476175021380524 0.2832032625629727 0.3177839055629352
0.3646456811464785 0.2820213372002091 0.3052620206229860
0.4492931339520498 0.4246958068607500 0.3971248757762245

FeOx_cluster I
1.00000000000000
15.0000000000000000 0.0000000000000000 0.0000000000000000
0.0000000000000000 15.0000000000000000 0.0000000000000000
0.0000000000000000 0.0000000000000000 15.0000000000000000
Fe O
5 6
Direct
0.5614958827053314 0.5331627397303060 0.2894363093822629
0.4658291368006861 0.6200758751555150 0.3831273039587283
0.4987221349164762 0.4887300780715566 0.4296282874082341
0.6805425451992312 0.5992858475244668 0.3916250974806249
0.5603223420489156 0.6122949594667741 0.5116091874930259
0.5265944358996767 0.5044265636074835 0.5440603537052221
0.4800901667626134 0.6146191253028032 0.2652167842824792
0.6759928965009692 0.6432484444990680 0.5011868246576354
0.4743871008962235 0.6865420327005678 0.4810479912673172
0.6790104558966256 0.5571845866666446 0.2814086426110677
0.5221829843732699 0.4296797922748112 0.3295531927534234

11. Coordinates of MgO Surfaces – PBE optimized

CONTCAR.100.1x1.vasp

```
Mg O
  1.00857736950000
    2.9777680768999999   0.0000000000000000   0.0000000000000000
    0.0000000000000000   2.9777680768999999   0.0000000000000000
    0.0000000000000000   0.0000000000000000  64.7455000000000069
  Mg   O
   20   20
Selective dynamics
Direct
  0.5000000000000000  0.5000000000000000  0.2601652515949056   T   T   T
  0.0000000000000000  0.0000000000000000  0.4227753280000002   F   F   F
  0.5000000000000000  0.5000000000000000  0.3902541488999987   F   F   F
  0.5000000000000000  0.5000000000000000  0.5203330747339479   T   T   T
  0.0000000000000000  0.0000000000000000  0.6825603172283508   T   T   T
  0.0000000000000000  0.0000000000000000  0.3577329698999989   F   F   F
  0.5000000000000000  0.5000000000000000  0.3252117907999974   F   F   F
  0.0000000000000000  0.0000000000000000  0.5528637761575439   T   T   T
  0.0000000000000000  0.0000000000000000  0.2926962721807911   T   T   T
  0.5000000000000000  0.5000000000000000  0.4552908466192136   T   T   T
  0.5000000000000000  0.5000000000000000  0.5853833217730795   T   T   T
  0.0000000000000000  0.0000000000000000  0.4878218672050991   T   T   T
  0.5000000000000000  0.5000000000000000  0.1951233426424537   T   T   T
  0.0000000000000000  0.0000000000000000  0.6178960057569043   T   T   T
  0.0000000000000000  0.0000000000000000  0.1626037970269181   T   T   T
  0.5000000000000000  0.5000000000000000  0.1300911130430933   T   T   T
  0.5000000000000000  0.5000000000000000  0.6504772854527729   T   T   T
  0.0000000000000000  0.0000000000000000  0.0975098333472317   T   T   T
  0.5000000000000000  0.5000000000000000  0.0654268015716539   T   T   T
  0.0000000000000000  0.0000000000000000  0.2276540439660550   T   T   T
  0.0000000000000000  0.0000000000000000  0.5853816510438890   T   T   T
  0.5000000000000000  0.5000000000000000  0.5528615479927979   T   T   T
  0.5000000000000000  0.5000000000000000  0.6179072920584048   T   T   T
  0.0000000000000000  0.0000000000000000  0.6503577177057025   T   T   T
  0.0000000000000000  0.0000000000000000  0.5203372212573285   T   T   T
  0.5000000000000000  0.5000000000000000  0.4878168318829097   T   T   T
  0.0000000000000000  0.0000000000000000  0.0646529214301736   T   T   T
  0.5000000000000000  0.5000000000000000  0.3577329698999989   F   F   F
  0.5000000000000000  0.5000000000000000  0.4227753280000002   F   F   F
  0.0000000000000000  0.0000000000000000  0.3902541488999987   F   F   F
  0.0000000000000000  0.0000000000000000  0.3252117907999974   F   F   F
  0.5000000000000000  0.5000000000000000  0.2926936921106815   T   T   T
  0.0000000000000000  0.0000000000000000  0.2601702869170950   T   T   T
  0.5000000000000000  0.5000000000000000  0.2276498974426744   T   T   T
  0.0000000000000000  0.0000000000000000  0.1951255708071997   T   T   T
  0.5000000000000000  0.5000000000000000  0.1626054677561086   T   T   T
  0.0000000000000000  0.0000000000000000  0.1300798267415928   T   T   T
  0.5000000000000000  0.5000000000000000  0.0976294010943022   T   T   T
  0.0000000000000000  0.0000000000000000  0.4552934266893232   T   T   T
```

```
  0.5000000000000000  0.5000000000000000  0.6833341973698310   T   T   T


CONTCAR.110.3x2.fac.vasp

Mg O
   1.00857736950000
     8.9333042307999992    0.0000000000000000    0.0000000000000000
     0.0000000000000000    8.4223999999999997    0.0000000000000000
     0.0000000000000000    0.0000000000000000   39.0000000000000000
   Mg   O
    74   74
Selective dynamics
Direct
  0.0169645690350961  0.0000012526496818  0.0794834189867331   T   T   T
  0.5004230765394908  0.7500000000000000  0.4198635696875996   T   T   T
  0.8333333332999970  0.7500000000000000  0.4201245353435823   T   T   T
  0.9985627802877488  0.0000002296271120  0.4574592752220283   T   T   T
  0.8333333332999970  0.2500000000000000  0.2672360059000027   F   F   F
  0.5000000000000000  0.2500000000000000  0.2672360059000027   F   F   F
  0.1666666667000030  0.2500000000000000  0.2672360059000027   F   F   F
  0.3333333332999970  0.0000001787777251  0.4604651706093250   T   T   T
  0.6681038864122542  0.0000002296271120  0.4574592752220283   T   T   T
  0.9985627802877488  0.4999997703728880  0.4574592752220283   T   T   T
  0.6662935683409685  0.5000034547915035  0.2291600342852078   T   T   T
  0.3333333332999970  0.5000001677139920  0.2286443667518085   T   T   T
  0.0003730983590344  0.5000034547915035  0.2291600342852078   T   T   T
  0.3333333332999970  0.4999998212222749  0.4604651706093250   T   T   T
  0.6681038864122542  0.4999997703728880  0.4574592752220283   T   T   T
  0.3333333332999970  0.5000000284464079  0.3821771231237108   T   T   T
  0.6662935683409685  0.9999965452084965  0.2291600342852078   T   T   T
  0.3333333332999970  0.9999998322860080  0.2286443667518085   T   T   T
  0.0003730983590344  0.9999965452084965  0.2291600342852078   T   T   T
  0.8333333332999970  0.7500000000000000  0.1906773568444038   T   T   T
  0.1666666667000030  0.7500000000000000  0.2672360059000027   F   F   F
  0.5003814462656067  0.7500000000000000  0.1909555144571797   T   T   T
  0.5000000000000000  0.7500000000000000  0.2672360059000027   F   F   F
  0.0000000000000000  0.0000000000000000  0.3054125196999991   F   F   F
  0.6662903971486784  0.4999999382280222  0.3816668157456675   T   T   T
  0.6662903971486784  0.0000000617719778  0.3816668157456675   T   T   T
  0.3333333332999970  0.9999999715535921  0.3821771231237108   T   T   T
  0.0003762695513245  0.0000000617719778  0.3816668157456675   T   T   T
  0.8333333332999970  0.7500000000000000  0.3435890335000025   F   F   F
  0.5000000000000000  0.7500000000000000  0.3435890335000025   F   F   F
  0.1666666667000030  0.7500000000000000  0.3435890335000025   F   F   F
  0.8333333332999970  0.2500000000000000  0.3435890335000025   F   F   F
  0.5000000000000000  0.2500000000000000  0.3435890335000025   F   F   F
  0.1666666667000030  0.2500000000000000  0.3435890335000025   F   F   F
  0.1662433756470989  0.2500000000000000  0.4198634466962403   T   T   T
  0.5004232910529041  0.2500000000000000  0.4198634466962403   T   T   T
  0.8333333332999970  0.2500000000000000  0.4201243404158177   T   T   T
  0.6666666667000030  0.5000000000000000  0.3054125196999991   F   F   F
```

0.3333333332999970	0.5000000000000000	0.3054125196999991	F	F	F
0.0000000000000000	0.5000000000000000	0.3054125196999991	F	F	F
0.1662435901605122	0.7500000000000000	0.4198635696875996	T	T	T
0.6666666667000030	0.0000000000000000	0.3054125196999991	F	F	F
0.3333333332999970	0.0000000000000000	0.3054125196999991	F	F	F
0.8333333332999970	0.7500000000000000	0.2672419258192491	T	T	T
0.1662852204343963	0.7500000000000000	0.1909555144571797	T	T	T
0.0003762695513245	0.4999999382280222	0.3816668157456675	T	T	T
0.0168161225236219	0.0000000271342913	0.5313206515546014	T	T	T
0.3333333332999970	0.0000015327727780	0.1503596609221418	T	T	T
0.9985584786467498	0.0000025768056346	0.1533452649864415	T	T	T
0.8333333332999970	0.7500000000000000	0.1095966502875427	T	T	T
0.5100884320624246	0.7500000000000000	0.1165198562047323	T	T	T
0.1565782346375784	0.7500000000000000	0.1165198562047323	T	T	T
0.5101811380320527	0.7500000000000000	0.4942841056087346	T	T	T
0.6681081880532531	0.0000025768056346	0.1533452649864415	T	T	T
0.8333333332999970	0.7500000000000000	0.5011853332276885	T	T	T
0.6497020976649068	0.4999987473503182	0.0794834189867331	T	T	T
0.5100883306859814	0.2500000000000000	0.1165205373732263	T	T	T
0.1565783360140216	0.2500000000000000	0.1165205373732263	T	T	T
0.0168161225236219	0.4999999728657087	0.5313206515546014	T	T	T
0.6498505441763811	0.0000000271342913	0.5313206515546014	T	T	T
0.8333333332999970	0.2500000000000000	0.1095980982794487	T	T	T
0.1564855286679503	0.7500000000000000	0.4942841056087346	T	T	T
0.3333333332999970	0.4999984672272220	0.1503596609221418	T	T	T
0.0169645690350961	0.4999987473503182	0.0794834189867331	T	T	T
0.6498505441763811	0.4999999728657087	0.5313206515546014	T	T	T
0.6681081880532531	0.4999974231943654	0.1533452649864415	T	T	T
0.8333333332999970	0.2500000000000000	0.1906795700741597	T	T	T
0.8333333332999970	0.2500000000000000	0.5011853108465800	T	T	T
0.5101811675530001	0.2500000000000000	0.4942840439696212	T	T	T
0.9985584786467498	0.4999974231943654	0.1533452649864415	T	T	T
0.1564854991470028	0.2500000000000000	0.4942840439696212	T	T	T
0.5003823133165071	0.2500000000000000	0.1909567672638488	T	T	T
0.6497020976649068	0.0000012526496818	0.0794834189867331	T	T	T
0.1662843533834959	0.2500000000000000	0.1909567672638488	T	T	T
0.3333333332999970	0.7500000000000000	0.3821702896897605	T	T	T
0.6666781494874883	0.7500000000000000	0.3816652143136849	T	T	T
0.1670522106105850	0.9999999460631415	0.4198819104282805	T	T	T
0.0160861597661111	0.7500000000000000	0.5339529904609748	T	T	T
0.6505804720768964	0.2500000000000000	0.5339529474768838	T	T	T
0.9999885172125147	0.7500000000000000	0.3816652143136849	T	T	T
0.4996144560894180	0.9999999460631415	0.4198819104282805	T	T	T
0.6700470283939595	0.7500000000000000	0.4575169483103281	T	T	T
0.1625852039970468	0.9999999927154377	0.4944566217188111	T	T	T
0.5040814627029562	0.9999999927154377	0.4944566217188111	T	T	T
0.8333333332999970	0.0000000435103615	0.4998064871197130	T	T	T
0.1625852039970468	0.5000000072845623	0.4944566217188111	T	T	T
0.6700470442751509	0.2500000000000000	0.4575168895217701	T	T	T
0.3333333332999970	0.2500000000000000	0.4602810590808986	T	T	T
0.8333333332999970	0.9999999229397645	0.4200744871951656	T	T	T
0.9966196224248520	0.2500000000000000	0.4575168895217701	T	T	T

0.8333333332999970	0.4999999564896385	0.4998064871197130	T	T	T
0.3333333332999970	0.7500000000000000	0.4602810782762177	T	T	T
0.8333333332999970	0.5000000770602355	0.4200744871951656	T	T	T
0.4996144560894180	0.5000000539368585	0.4198819104282805	T	T	T
0.1670522106105850	0.5000000539368585	0.4198819104282805	T	T	T
0.0160861946231066	0.2500000000000000	0.5339529474768838	T	T	T
0.5040814627029562	0.5000000072845623	0.4944566217188111	T	T	T
0.9966196383060435	0.7500000000000000	0.4575169483103281	T	T	T
0.6666666667000030	0.2500000000000000	0.3054125196999991	F	F	F
0.3333333332999970	0.2500000000000000	0.3821702929419288	T	T	T
0.1670614803983455	0.4999995521467966	0.1909529273751360	T	T	T
0.8333333332999970	0.9999997445505358	0.1907474877074975	T	T	T
0.4996051863016575	0.0000004478532034	0.1909529273751360	T	T	T
0.1670614803983455	0.0000004478532034	0.1909529273751360	T	T	T
0.6700227550249807	0.7500000000000000	0.1532770262141270	T	T	T
0.3333333332999970	0.7500000000000000	0.1505342930980547	T	T	T
0.9966439116750223	0.7500000000000000	0.1532770262141270	T	T	T
0.6700234972470724	0.2500000000000000	0.1532782685885508	T	T	T
0.3333333332999970	0.2500000000000000	0.1505344221385698	T	T	T
0.9966431694529305	0.2500000000000000	0.1532782685885508	T	T	T
0.4996051863016575	0.4999995521467966	0.1909529273751360	T	T	T
0.8333333332999970	0.4999992729654963	0.1109967631672575	T	T	T
0.1626630541610723	0.4999993651490087	0.1163716391554530	T	T	T
0.8333333332999970	0.0000007270345037	0.1109967631672575	T	T	T
0.5040036125389307	0.0000006348509913	0.1163716391554530	T	T	T
0.1626630541610723	0.0000006348509913	0.1163716391554530	T	T	T
0.6504414191939034	0.7500000000000000	0.0768386221142805	T	T	T
0.0162252475060995	0.7500000000000000	0.0768386221142805	T	T	T
0.6504422864511241	0.2500000000000000	0.0768394794353284	T	T	T
0.0162243802488788	0.2500000000000000	0.0768394794353284	T	T	T
0.5040036125389307	0.4999993651490087	0.1163716391554530	T	T	T
0.8333333332999970	0.5000002554494642	0.1907474877074975	T	T	T
0.9999848739359578	0.2500000000000000	0.2291536851880167	T	T	T
0.3333333332999970	0.2500000000000000	0.2286512925870667	T	T	T
0.9999885457531121	0.2500000000000000	0.3816653751278452	T	T	T
0.8333333332999970	0.5000000000000000	0.3435890335000025	F	F	F
0.5000000000000000	0.5000000000000000	0.3435890335000025	F	F	F
0.1666666667000030	0.5000000000000000	0.3435890335000025	F	F	F
0.8333333332999970	0.0000000000000000	0.3435890335000025	F	F	F
0.5000000000000000	0.0000000000000000	0.3435890335000025	F	F	F
0.1666666667000030	0.0000000000000000	0.3435890335000025	F	F	F
0.6666666667000030	0.7500000000000000	0.3054125196999991	F	F	F
0.3333333332999970	0.7500000000000000	0.3054125196999991	F	F	F
0.0000000000000000	0.7500000000000000	0.3054125196999991	F	F	F
0.3333333332999970	0.2500000000000000	0.3054125196999991	F	F	F
0.0000000000000000	0.2500000000000000	0.3054125196999991	F	F	F
0.8333333332999970	0.5000000000000000	0.2672360059000027	F	F	F
0.5000000000000000	0.5000000000000000	0.2672360059000027	F	F	F
0.1666666667000030	0.5000000000000000	0.2672360059000027	F	F	F
0.8333333332999970	0.0000000000000000	0.2672360059000027	F	F	F
0.5000000000000000	0.0000000000000000	0.2672360059000027	F	F	F
0.1666666667000030	0.0000000000000000	0.2672360059000027	F	F	F

```
  0.6666827971422151  0.7500000000000000  0.2291537873293521   T   T   T
  0.3333333332999970  0.7500000000000000  0.2286509241282815   T   T   T
  0.9999838695577878  0.7500000000000000  0.2291537873293521   T   T   T
  0.6666817927640452  0.2500000000000000  0.2291536851880167   T   T   T
  0.6666781209468908  0.2500000000000000  0.3816653751278452   T   T   T
  0.6505805069338919  0.7500000000000000  0.5339529904609748   T   T   T
```

CONTCAR.110.3x2.stoich.vasp

```
Mg O
   1.00857736950000
     8.9333042307999992    0.0000000000000000    0.0000000000000000
     0.0000000000000000    8.4223999999999997    0.0000000000000000
     0.0000000000000000    0.0000000000000000   39.0000000000000000
   Mg   O
    78   78
Selective dynamics
Direct
  0.0000008701499468  0.0000030217764291  0.0795073356758280   T   T   T
  0.5000011833186448  0.7500000000000000  0.4203706852960067   T   T   T
  0.8333333332999970  0.7500000000000000  0.4203708729682845   T   T   T
  0.9999999851890706  0.0000000864454961  0.4576279164704928   T   T   T
  0.8333333332999970  0.2500000000000000  0.2672360059000027   F   F   F
  0.5000000000000000  0.2500000000000000  0.2672360059000027   F   F   F
  0.1666666667000030  0.2500000000000000  0.2672360059000027   F   F   F
  0.3333333332999970  0.0000000974264296  0.4576279936341408   T   T   T
  0.6666666815109394  0.0000000864454961  0.4576279164704928   T   T   T
  0.9999999851890706  0.4999999135545110  0.4576279164704928   T   T   T
  0.6666486590782981  0.4999577832718032  0.2290910482773754   T   T   T
  0.3333333332999970  0.5000009182182197  0.2290955174860869   T   T   T
  0.0000180076217049  0.4999577832718032  0.2290910482773754   T   T   T
  0.3333333332999970  0.4999999025735633  0.4576279936341408   T   T   T
  0.6666666815109394  0.4999999135545110  0.4576279164704928   T   T   T
  0.3333333332999970  0.5000002433649371  0.3817513970093316   T   T   T
  0.6666486590782981  0.0000422167281968  0.2290910482773754   T   T   T
  0.3333333332999970  0.9999990817817803  0.2290955174860869   T   T   T
  0.0000180076217049  0.0000422167281968  0.2290910482773754   T   T   T
  0.8333333332999970  0.7500000000000000  0.1904029725650531   T   T   T
  0.1666666667000030  0.7500000000000000  0.2672360059000027   F   F   F
  0.4999917805504381  0.7500000000000000  0.1904240511044080   T   T   T
  0.5000000000000000  0.7500000000000000  0.2672360059000027   F   F   F
  0.0000000000000000  0.0000000000000000  0.3054125196999991   F   F   F
  0.6666693133450394  0.5000026475542612  0.3817505244271402   T   T   T
  0.6666693133450394  0.9999973524457388  0.3817505244271402   T   T   T
  0.3333333332999970  0.9999997566350700  0.3817513970093316   T   T   T
  0.9999973533549564  0.9999973524457388  0.3817505244271402   T   T   T
  0.8333333332999970  0.7500000000000000  0.3435890335000025   F   F   F
  0.5000000000000000  0.7500000000000000  0.3435890335000025   F   F   F
  0.1666666667000030  0.7500000000000000  0.3435890335000025   F   F   F
  0.8333333332999970  0.2500000000000000  0.3435890335000025   F   F   F
  0.5000000000000000  0.2500000000000000  0.3435890335000025   F   F   F
  0.1666666667000030  0.2500000000000000  0.3435890335000025   F   F   F
```

0.1666658474213136	0.2500000000000000	0.4203705316753101	T	T	T
0.5000008192786893	0.2500000000000000	0.4203705316753101	T	T	T
0.8333333332999970	0.2500000000000000	0.4203704814474776	T	T	T
0.6666666667000030	0.5000000000000000	0.3054125196999991	F	F	F
0.3333333332999970	0.5000000000000000	0.3054125196999991	F	F	F
0.0000000000000000	0.5000000000000000	0.3054125196999991	F	F	F
0.1666654833813581	0.7500000000000000	0.4203706852960067	T	T	T
0.6666666667000030	0.0000000000000000	0.3054125196999991	F	F	F
0.3333333332999970	0.0000000000000000	0.3054125196999991	F	F	F
0.8333333332999970	0.7500000000000000	0.2671300105638181	T	T	T
0.1666748861495648	0.7500000000000000	0.1904240511044080	T	T	T
0.9999973533549564	0.5000026475542612	0.3817505244271402	T	T	T
0.0000037432998425	0.0000037243448290	0.5312725782924090	T	T	T
0.3333333332999970	0.0000057245726239	0.1531822509750569	T	T	T
0.0000041992966331	0.0000074560824501	0.1531817382656868	T	T	T
0.8333333332999970	0.7500000000000000	0.1122190264910756	T	T	T
0.4999994099304601	0.7500000000000000	0.1122219655474694	T	T	T
0.1666672567695429	0.7500000000000000	0.1122219655474694	T	T	T
0.5000002345747916	0.7500000000000000	0.4985571811779721	T	T	T
0.6666624674033699	0.0000074560824501	0.1531817382656868	T	T	T
0.8333333332999970	0.7500000000000000	0.4985571918652170	T	T	T
0.6666657965500491	0.4999969782235709	0.0795073356758280	T	T	T
0.5000006843267357	0.2500000000000000	0.1122232720583938	T	T	T
0.1666659823732672	0.2500000000000000	0.1122232720583938	T	T	T
0.3333333332999970	0.9999999854234076	0.5312725134744838	T	T	T
0.0000037432998425	0.4999962756551710	0.5312725782924090	T	T	T
0.6666629234001533	0.0000037243448290	0.5312725782924090	T	T	T
0.8333333332999970	0.2500000000000000	0.1122231433524092	T	T	T
0.3333333332999970	0.4999956883675907	0.0795076670857284	T	T	T
0.1666664321252114	0.7500000000000000	0.4985571811779721	T	T	T
0.3333333332999970	0.0000043116324022	0.0795076670857284	T	T	T
0.3333333332999970	0.4999942754273761	0.1531822509750569	T	T	T
0.0000008701499468	0.4999969782235709	0.0795073356758280	T	T	T
0.3333333332999970	0.5000000145765924	0.5312725134744838	T	T	T
0.6666629234001533	0.4999962756551710	0.5312725782924090	T	T	T
0.6666624674033699	0.4999925439175499	0.1531817382656868	T	T	T
0.8333333332999970	0.2500000000000000	0.1904240964315633	T	T	T
0.8333333332999970	0.2500000000000000	0.4985571484622966	T	T	T
0.5000000267839013	0.2500000000000000	0.4985571576874506	T	T	T
0.0000041992966331	0.4999925439175499	0.1531817382656868	T	T	T
0.1666666399161016	0.2500000000000000	0.4985571576874506	T	T	T
0.4999896689273839	0.2500000000000000	0.1904251912582922	T	T	T
0.6666657965500491	0.0000030217764291	0.0795073356758280	T	T	T
0.1666769977726190	0.2500000000000000	0.1904251912582922	T	T	T
0.3333333332999970	0.7500000000000000	0.3818153280554597	T	T	T
0.6666657417475719	0.7500000000000000	0.3818173117170289	T	T	T
0.1666669182794180	0.0000006745084420	0.4201505328278117	T	T	T
0.9999999925772727	0.7500000000000000	0.5335748873453241	T	T	T
0.6666666309655582	0.2500000000000000	0.5335747914731925	T	T	T
0.0000009249524311	0.7500000000000000	0.3818173117170289	T	T	T
0.4999997484205849	0.0000006745084420	0.4201505328278117	T	T	T
0.6666666747137526	0.7500000000000000	0.4581336374032148	T	T	T

0.3333333332999970	0.2500000000000000	0.5335747724640001	T	T	T
0.1666667451848198	0.0000001143584285	0.4970226594611091	T	T	T
0.4999999215151831	0.0000001143584285	0.4970226594611091	T	T	T
0.8333333332999970	0.0000002496619445	0.4970227212729839	T	T	T
0.1666667451848198	0.4999998856415786	0.4970226594611091	T	T	T
0.6666665771400062	0.2500000000000000	0.4581331718763693	T	T	T
0.3333333332999970	0.2500000000000000	0.4581330824613090	T	T	T
0.8333333332999970	0.0000011009772010	0.4201507965886293	T	T	T
0.0000000895600039	0.2500000000000000	0.4581331718763693	T	T	T
0.8333333332999970	0.4999997503380484	0.4970227212729839	T	T	T
0.3333333332999970	0.7500000000000000	0.4581331053182680	T	T	T
0.8333333332999970	0.4999988990227990	0.4201507965886293	T	T	T
0.4999997484205849	0.4999993254915580	0.4201505328278117	T	T	T
0.1666669182794180	0.4999993254915580	0.4201505328278117	T	T	T
0.0000000357344447	0.2500000000000000	0.5335747914731925	T	T	T
0.4999999215151831	0.4999998856415786	0.4970226594611091	T	T	T
0.9999999919862503	0.7500000000000000	0.4581336374032148	T	T	T
0.6666666667000030	0.2500000000000000	0.3054125196999991	F	F	F
0.3333333332999970	0.2500000000000000	0.3818150136488256	T	T	T
0.1666695835878116	0.4999974681994459	0.1906849132977229	T	T	T
0.8333333332999970	0.0000019687429358	0.1906840240869911	T	T	T
0.4999970831121985	0.0000025318005541	0.1906849132977229	T	T	T
0.1666695835878116	0.0000025318005541	0.1906849132977229	T	T	T
0.6666635101956828	0.7500000000000000	0.1526593064614872	T	T	T
0.3333333332999970	0.7500000000000000	0.1526621067261118	T	T	T
0.0000031565043201	0.7500000000000000	0.1526593064614872	T	T	T
0.6666659342350343	0.2500000000000000	0.1526650173500528	T	T	T
0.3333333332999970	0.2500000000000000	0.1526647024372636	T	T	T
0.0000007324649758	0.2500000000000000	0.1526650173500528	T	T	T
0.4999970831121985	0.4999974681994459	0.1906849132977229	T	T	T
0.8333333332999970	0.4999985760331285	0.1137577321604084	T	T	T
0.1666672411741388	0.4999947546689754	0.1137586246411288	T	T	T
0.8333333332999970	0.0000014239668786	0.1137577321604084	T	T	T
0.4999994255258642	0.0000052453310246	0.1137586246411288	T	T	T
0.1666672411741388	0.0000052453310246	0.1137586246411288	T	T	T
0.6666642291012437	0.7500000000000000	0.0772136206367549	T	T	T
0.3333333332999970	0.7500000000000000	0.0772140965166201	T	T	T
0.0000024375987664	0.7500000000000000	0.0772136206367549	T	T	T
0.6666668710054751	0.2500000000000000	0.0772150740319333	T	T	T
0.3333333332999970	0.2500000000000000	0.0772152105288697	T	T	T
0.9999997956945279	0.2500000000000000	0.0772150740319333	T	T	T
0.4999994255258642	0.4999947546689754	0.1137586246411288	T	T	T
0.8333333332999970	0.4999980312570571	0.1906840240869911	T	T	T
0.0000040307333506	0.2500000000000000	0.2289976023258902	T	T	T
0.3333333332999970	0.2500000000000000	0.2289988223665773	T	T	T
0.0000009650872030	0.2500000000000000	0.3818151299965677	T	T	T
0.8333333332999970	0.5000000000000000	0.3435890335000025	F	F	F
0.5000000000000000	0.5000000000000000	0.3435890335000025	F	F	F
0.1666666667000030	0.5000000000000000	0.3435890335000025	F	F	F
0.8333333332999970	0.0000000000000000	0.3435890335000025	F	F	F
0.5000000000000000	0.0000000000000000	0.3435890335000025	F	F	F
0.1666666667000030	0.0000000000000000	0.3435890335000025	F	F	F

```
  0.6666666667000030  0.7500000000000000  0.3054125196999991   F   F   F
  0.3333333332999970  0.7500000000000000  0.3054125196999991   F   F   F
  0.0000000000000000  0.7500000000000000  0.3054125196999991   F   F   F
  0.3333333332999970  0.7500000000000000  0.5335747606299250   T   T   T
  0.3333333332999970  0.2500000000000000  0.3054125196999991   F   F   F
  0.0000000000000000  0.2500000000000000  0.3054125196999991   F   F   F
  0.8333333332999970  0.5000000000000000  0.2672360059000027   F   F   F
  0.5000000000000000  0.5000000000000000  0.2672360059000027   F   F   F
  0.1666666667000030  0.5000000000000000  0.2672360059000027   F   F   F
  0.8333333332999970  0.0000000000000000  0.2672360059000027   F   F   F
  0.5000000000000000  0.0000000000000000  0.2672360059000027   F   F   F
  0.1666666667000030  0.0000000000000000  0.2672360059000027   F   F   F
  0.6666591387765806  0.7500000000000000  0.2289836970413646   T   T   T
  0.3333333332999970  0.7500000000000000  0.2289978594760598   T   T   T
  0.0000075279234224  0.7500000000000000  0.2289836970413646   T   T   T
  0.6666626359666523  0.2500000000000000  0.2289976023258902   T   T   T
  0.6666657016127999  0.2500000000000000  0.3818151299965677   T   T   T
  0.6666666741227303  0.7500000000000000  0.5335748873453241   T   T   T
```

CONTCAR.111.2x2.octo.vasp

```
Mg O
   1.00857736950000
     5.9555361538999998    0.0000000000000000    0.0000000000000000
    -2.9777680768999999    5.1576456023999997    0.0000000000000000
     0.0000000000000000    0.0000000000000000   39.1760745361000033
   Mg   O
    34    34
Selective dynamics
Direct
  0.8333333332999970  0.6666666667000030  0.3723698057999982   F   F   F
 -0.0013057591600608  0.4993471204199689  0.3103410689147625   T   T   T
  0.5006528795800270  0.4993471204199689  0.3103410689147625   T   T   T
  0.3333333332999970  0.1666666667000030  0.3723698057999982   F   F   F
  0.8333333332999970  0.1666666667000030  0.3723698057999982   F   F   F
  0.3333333332999970  0.6666666667000030  0.3723698057999982   F   F   F
  0.1666666667000030  0.3333333332999970  0.4344315995000017   F   F   F
  0.6666666667000030  0.3333333332999970  0.4344315995000017   F   F   F
  0.1666666667000030  0.8333333332999970  0.4344315995000017   F   F   F
  0.6666666667000030  0.8333333332999970  0.4344315995000017   F   F   F
 -0.0000000000000000 -0.0000000000000000  0.4967931282924082   T   T   T
  0.5006528795800270  0.0013057591600608  0.3103410689147625   T   T   T
  0.4993471204199689 -0.0013057591600608  0.4964603363852373   T   T   T
  0.4993471204199689  0.5006528795800270  0.4964603363852373   T   T   T
  0.3379727456574911  0.1689863728787500  0.5587225343158587   T   T   T
  0.8310136271212545  0.1689863728787500  0.5587225343158587   T   T   T
  0.3333333332999970  0.6666666667000030  0.5586378057937947   T   T   T
  0.8310136271212545  0.6620272543425080  0.5587225343158587   T   T   T
  0.1573044819613761  0.3146089638227432  0.6183226233854572   T   T   T
  0.6666666667000030  0.3333333332999970  0.6316274540693535   T   T   T
  0.1573044819613761  0.8426955180386313  0.6183226233854572   T   T   T
```

0.6853910361772615	0.8426955180386313	0.6183226233854572	T	T	T
-0.0000000000000000	-0.0000000000000000	0.6736835080141940	T	T	T
0.0013057591600608	0.5006528795800270	0.4964603363852373	T	T	T
-0.0000000000000000	-0.0000000000000000	0.3100082770075917	T	T	T
0.8426955180386313	0.1573044819613761	0.1884787819145445	T	T	T
0.3146089638227432	0.1573044819613761	0.1884787819145445	T	T	T
0.3333333332999970	0.6666666667000030	0.1751739512306378	T	T	T
0.8426955180386313	0.6853910361772615	0.1884787819145445	T	T	T
-0.0000000000000000	-0.0000000000000000	0.1331178972858017	T	T	T
0.1689863728787500	0.3379727456574911	0.2480788709841328	T	T	T
0.6666666667000030	0.3333333332999970	0.2481635995062091	T	T	T
0.6620272543425080	0.8310136271212545	0.2480788709841328	T	T	T
0.1689863728787500	0.8310136271212545	0.2480788709841328	T	T	T
0.3147402418283218	0.1573701209641654	0.6495866701307247	T	T	T
0.6654057631929775	0.8327028815464819	0.5274266484162030	T	T	T
0.1573701209641654	0.8426298790358345	0.1572147351692773	T	T	T
0.1573701209641654	0.3147402418283218	0.1572147351692773	T	T	T
0.8426298790358345	0.6852597581716751	0.6495866701307247	T	T	T
0.4954094762116611	-0.0091810475766768	0.5906210980976604	T	T	T
0.0091810475766768	0.5045905237883405	0.5906210980976604	T	T	T
0.4954094762116611	0.5045905237883405	0.5906210980976604	T	T	T
0.1672971184535153	0.8327028815464819	0.5274266484162030	T	T	T
0.8426298790358345	0.1573701209641654	0.6495866701307247	T	T	T
-0.0000000000000000	-0.0000000000000000	0.5868600521838991	T	T	T
0.6666666667000030	0.3333333332999970	0.5281261854803808	T	T	T
0.3333333332999970	0.6666666667000030	0.2786752198196215	T	T	T
0.6852597581716751	0.8426298790358345	0.1572147351692773	T	T	T
0.8327028815464819	0.1672971184535154	0.2793747568837959	T	T	T
0.3345942368070218	0.1672971184535154	0.2793747568837959	T	T	T
0.1668560355889877	0.3337120710779665	0.3412542164676685	T	T	T
0.6666666667000030	0.3333333332999970	0.3415122577344423	T	T	T
0.1668560355889877	0.8331439644110141	0.3412542164676685	T	T	T
0.6662879289220356	0.8331439644110141	0.3412542164676685	T	T	T
0.5045905237883405	0.4954094762116611	0.2161803072023367	T	T	T
0.0000000000000000	0.0000000000000000	0.4034007026000026	F	F	F
0.5000000000000000	0.0000000000000000	0.4034007026000026	F	F	F
0.0000000000000000	0.5000000000000000	0.4034007026000026	F	F	F
0.5000000000000000	0.5000000000000000	0.4034007026000026	F	F	F
-0.0091810475766768	0.4954094762116611	0.2161803072023367	T	T	T
0.5045905237883405	0.0091810475766768	0.2161803072023367	T	T	T
-0.0000000000000000	-0.0000000000000000	0.2199413531160991	T	T	T
0.3337120710779665	0.1668560355889877	0.4655471888323314	T	T	T
0.8331439644110141	0.1668560355889877	0.4655471888323314	T	T	T
0.3333333332999970	0.6666666667000030	0.4652891475655576	T	T	T
0.8331439644110141	0.6662879289220356	0.4655471888323314	T	T	T
0.8327028815464819	0.6654057631929775	0.2793747568837959	T	T	T
0.1672971184535153	0.3345942368070218	0.5274266484162030	T	T	T

```
CONTCAR.111.stoich.vasp

Mg O
  1.00857736950000
    5.9555361538999998    0.0000000000000000    0.0000000000000000
   -2.9777680768999999    5.1576456023999997    0.0000000000000000
    0.0000000000000000    0.0000000000000000   39.1760745361000033
  Mg   O
   40    40
Selective dynamics
Direct
  0.8333333333193380  0.6666666666666643  0.3723698058251728   F   F   F
  0.9999999999916014  0.5000000000000000  0.3103080121822259   F   F   F
  0.5000000000000000  0.5000000000000000  0.3103080121822259   F   F   F
  0.3333333333361352  0.1666666666666643  0.3723698058251728   F   F   F
  0.8333333333277366  0.1666666666666643  0.3723698058251728   F   F   F
  0.3333333333277366  0.6666666666666643  0.3723698058251728   F   F   F
  0.9999999999916014  0.5000000000000000  0.6832897916141808   T   T   T
  0.1666666666638648  0.3333333333333357  0.4344315994706704   F   F   F
  0.6666666666554733  0.3333333333333357  0.4344315994706704   F   F   F
  0.1666666666554733  0.8333333333333357  0.4344315994706704   F   F   F
  0.6666666666638719  0.8333333333333357  0.4344315994706704   F   F   F
 -0.0000000000000000  0.0000000000000000  0.4958583013902639   T   T   T
  0.4999999999916014  0.0000000000000000  0.3103080121822259   F   F   F
  0.4999999999916014  0.0000000000000000  0.4958583013902639   T   T   T
  0.5000000000000000  0.5000000000000000  0.4958583013902639   T   T   T
  0.3333333333361352  0.1666666666666643  0.5579557483128058   T   T   T
  0.8333333333277366  0.1666666666666643  0.5579557483128058   T   T   T
  0.3333333333277366  0.6666666666666643  0.5579557483128058   T   T   T
  0.8333333333193380  0.6666666666666643  0.5579557483128058   T   T   T
  0.1666666666638648  0.3333333333333357  0.6200552393049236   T   T   T
  0.6666666666554733  0.3333333333333357  0.6200552393049236   T   T   T
  0.1666666666554733  0.8333333333333357  0.6200552393049236   T   T   T
  0.6666666666638719  0.8333333333333357  0.6200552393049236   T   T   T
  0.0000000000000000 -0.0000000000000000  0.6832897916141808   T   T   T
  0.4999999999916014 -0.0000000000000000  0.6832897916141808   T   T   T
  0.9999999999916014  0.5000000000000000  0.4958583013902639   T   T   T
  0.0000000000000000  0.0000000000000000  0.3103080121822259   F   F   F
  0.5000000000000000  0.5000000000000000  0.6832897916141808   T   T   T
  0.8333333333277366  0.1666666666666643  0.1855992098616994   T   T   T
  0.3333333333361352  0.1666666666666643  0.1855992098616994   T   T   T
  0.3333333333277366  0.6666666666666643  0.1855992098616994   T   T   T
  0.5000000000000000  0.5000000000000000  0.1219160472007249   T   T   T
  0.8333333333193380  0.6666666666666643  0.1855992098616994   T   T   T
  0.4999999999916014  0.0000000000000000  0.1219160472007249   T   T   T
  0.0000000000000000 -0.0000000000000000  0.1219160472007249   T   T   T
  0.1666666666638648  0.3333333333333357  0.2480350945996805   T   T   T
  0.6666666666554733  0.3333333333333357  0.2480350945996805   T   T   T
  0.9999999999916014  0.5000000000000000  0.1219160472007249   T   T   T
  0.6666666666638719  0.8333333333333357  0.2480350945996805   T   T   T
  0.1666666666554733  0.8333333333333357  0.2480350945996805   T   T   T
  0.3333333333361352  0.1666666666666643  0.6518448778546981   T   T   T
```

0.8333333333277366	0.1666666666666643	0.0985351659027428	T	T	T
0.6666666666638719	0.8333333333333357	0.5277222154935479	T	T	T
0.3333333333277366	0.6666666666666643	0.0985351659027428	T	T	T
0.1666666666554733	0.8333333333333357	0.1565484957717604	T	T	T
0.6666666666554733	0.3333333333333357	0.1565484957717604	T	T	T
0.1666666666638648	0.3333333333333357	0.1565484957717604	T	T	T
0.8333333333193380	0.6666666666666643	0.6518448778546981	T	T	T
0.4999999999916014	-0.0000000000000000	0.5898325335902870	T	T	T
0.9999999999916014	0.5000000000000000	0.5898325335902870	T	T	T
0.5000000000000000	0.5000000000000000	0.5898325335902870	T	T	T
0.1666666666554733	0.8333333333333357	0.5277222154935479	T	T	T
0.3333333333277366	0.6666666666666643	0.6518448778546981	T	T	T
0.8333333333277366	0.1666666666666643	0.6518448778546981	T	T	T
0.8333333333193380	0.6666666666666643	0.0985351659027428	T	T	T
-0.0000000000000000	-0.0000000000000000	0.5898325335902870	T	T	T
0.6666666666554733	0.3333333333333357	0.5277222154935479	T	T	T
0.3333333333277366	0.6666666666666643	0.2800039246669807	T	T	T
0.6666666666638719	0.8333333333333357	0.1565484957717604	T	T	T
0.8333333333277366	0.1666666666666643	0.2800039246669807	T	T	T
0.3333333333361352	0.1666666666666643	0.2800039246669808	T	T	T
0.1666666666638648	0.3333333333333357	0.3413389090062537	F	F	F
0.6666666666554733	0.3333333333333357	0.3413389090062537	F	F	F
0.1666666666554733	0.8333333333333357	0.3413389090062537	F	F	F
0.6666666666638719	0.8333333333333357	0.3413389090062537	F	F	F
0.5000000000000000	0.5000000000000000	0.2179240054424862	T	T	T
0.0000000000000000	0.0000000000000000	0.4034007026491935	F	F	F
0.4999999999916014	0.0000000000000000	0.4034007026491935	F	F	F
0.9999999999916014	0.5000000000000000	0.4034007026491935	F	F	F
0.5000000000000000	0.5000000000000000	0.4034007026491935	F	F	F
0.9999999999916014	0.5000000000000000	0.2179240054424861	T	T	T
0.4999999999916014	-0.0000000000000000	0.2179240054424861	T	T	T
-0.0000000000000000	-0.0000000000000000	0.2179240054424861	T	T	T
0.3333333333361352	0.1666666666666643	0.4654624962921403	F	F	F
0.8333333333277366	0.1666666666666643	0.4654624962921403	F	F	F
0.3333333333277366	0.6666666666666643	0.4654624962921403	F	F	F
0.8333333333193380	0.6666666666666643	0.4654624962921403	F	F	F
0.8333333333193380	0.6666666666666643	0.2800039246669807	T	T	T
0.1666666666638648	0.3333333333333357	0.5277222154935479	T	T	T
0.3333333333361352	0.1666666666666643	0.0985351659027428	T	T	T

12. Coordinates of MgO Surfaces – MLFF optimized

FFcluster:

```
100_1x1/CONTCAR
Mg O
   1.00857736950000
     2.9777680768999999    0.0000000000000000    0.0000000000000000
     0.0000000000000000    2.9777680768999999    0.0000000000000000
     0.0000000000000000    0.0000000000000000   64.7455000000000069
   Mg   O
    20    20
Selective dynamics
Direct
  0.5000000000001008  0.4999999999997818  0.2646604764673944   T   T   T
  0.0000000000000000  0.0000000000000000  0.4227753280000002   F   F   F
  0.5000000000000000  0.5000000000000000  0.3902541488999987   F   F   F
  0.4999999999999812  0.4999999999999186  0.5136294786231624   T   T   T
 -0.0000000000000019 -0.0000000000000507  0.6651019799194606   T   T   T
  0.0000000000000000  0.0000000000000000  0.3577329698999989   F   F   F
  0.5000000000000000  0.5000000000000000  0.3252117907999974   F   F   F
  0.0000000000000041  0.0000000000002412  0.5438905818059044   T   T   T
 -0.0000000000001822  0.0000000000001376  0.2950202340060238   T   T   T
  0.5000000000000301  0.5000000000000266  0.4529668847915971   T   T   T
  0.4999999999999404  0.4999999999997836  0.5741896036173001   T   T   T
  0.0000000000000111 -0.0000000000000311  0.4833266423289419   T   T   T
  0.4999999999993536  0.4999999999999458  0.2040965369903706   T   T   T
  0.0000000000001153 -0.0000000000000171  0.6042730077271817   T   T   T
  0.0000000000006755  0.0000000000000548  0.1737975151791945   T   T   T
  0.4999999999994253  0.4999999999997864  0.1437141110689672   T   T   T
  0.4999999999999089  0.5000000000001386  0.6345998463999791   T   T   T
  0.0000000000002622  0.0000000000001910  0.1133872723955188   T   T   T
  0.5000000000000087  0.4999999999999927  0.0828851388751713   T   T   T
  0.0000000000003281  0.0000000000001660  0.2343576401728647   T   T   T
 -0.0000000000000734 -0.0000000000002569  0.5741335967508008   T   T   T
  0.5000000000000054  0.5000000000002864  0.5439362218201610   T   T   T
  0.5000000000001368  0.4999999999999775  0.6045891481834278   T   T   T
 -0.0000000000001078  0.0000000000001507  0.6344642398646514   T   T   T
 -0.0000000000000249 -0.0000000000001030  0.5136049551829762   T   T   T
  0.5000000000000139  0.4999999999999695  0.4833346016600443   T   T   T
  0.0000000000000017 -0.0000000000000202  0.0815325425191459   T   T   T
  0.5000000000000000  0.5000000000000000  0.3577329698999989   F   F   F
  0.5000000000000000  0.5000000000000000  0.4227753280000002   F   F   F
  0.0000000000000000  0.0000000000000000  0.3902541488999987   F   F   F
  0.0000000000000000  0.0000000000000000  0.3252117907999974   F   F   F
  0.4999999999997890  0.5000000000001708  0.2950827843222180   T   T   T
  0.0000000000001074 -0.0000000000002680  0.2646525171363937   T   T   T
  0.5000000000003958  0.5000000000002031  0.2343821636130917   T   T   T
 -0.0000000000007894 -0.0000000000000694  0.2040508969761251   T   T   T
  0.5000000000008308  0.5000000000000773  0.1738535220456200   T   T   T
 -0.0000000000006895 -0.0000000000002576  0.1433979706126770   T   T   T
  0.5000000000003231  0.5000000000002156  0.1135228789306760   T   T   T
```

```
  0.0000000000000315  0.0000000000000292  0.4529043344754393   T   T   T
  0.5000000000000020  0.4999999999999236  0.6664545762752460   T   T   T

110_fac/CONTCAR
Mg O
   1.00857736950000
     8.9333042307999992    0.0000000000000000    0.0000000000000000
     0.0000000000000000    8.4223999999999997    0.0000000000000000
     0.0000000000000000    0.0000000000000000   39.0000000000000000
   Mg   O
    74    74
Selective dynamics
Direct
  0.0317191175090188  0.0000944328873250  0.0829406647520538   T   T   T
  0.5086360444575095  0.7500000000000018  0.4114940225917635   T   T   T
  0.8333333337251877  0.7500000000000027  0.4235150376314832   T   T   T
  1.0141855267089290  0.0000042997013254  0.4491509457684525   T   T   T
  0.8333333332999970  0.2500000000000000  0.2672360059000027   F   F   F
  0.5000000000000000  0.2500000000000000  0.2672360059000027   F   F   F
  0.1666666667000030  0.2500000000000000  0.2672360059000027   F   F   F
  0.3333333321724871 -0.0000520209428219  0.4629616536698486   T   T   T
  0.6524811398061844  0.0000042997042352  0.4491509455639740   T   T   T
  1.0141855267089279  0.4999957002986827  0.4491509457684520   T   T   T
  0.6659118099055439  0.5006492226380893  0.2342957762378349   T   T   T
  0.3333333333958498  0.4999430786990539  0.2297220876775734   T   T   T
  0.0007548572966938  0.5006492225756971  0.2342957760917103   T   T   T
  0.3333333321724869  0.5000520209428287  0.4629616536698494   T   T   T
  0.6524811398061846  0.4999957002957705  0.4491509455639741   T   T   T
  0.3333333333928682  0.5000174960706620  0.3810836020075810   T   T   T
  0.6659118099055429  0.9993507773619065  0.2342957762378355   T   T   T
  0.3333333333958500  1.0000569213009416  0.2297220876775732   T   T   T
  0.0007548572966952  0.9993507774242993  0.2342957760917104   T   T   T
  0.8333333336972386  0.7499999999999972  0.1878941027430582   T   T   T
  0.1666666667000030  0.7500000000000000  0.2672360059000027   F   F   F
  0.5091941490588341  0.7499999999999961  0.1995212033685336   T   T   T
  0.5000000000000000  0.7500000000000000  0.2672360059000027   F   F   F
  0.0000000000000000  0.0000000000000000  0.3054125196999991   F   F   F
  0.6652482033856764  0.4999443132148532  0.3765112558479414   T   T   T
  0.6652482033856748  0.0000556867851497  0.3765112558479413   T   T   T
  0.3333333333928680  0.9999825039293412  0.3810836020075813   T   T   T
  0.0014184638875137  0.0000556867838877  0.3765112560118514   T   T   T
  0.8333333332999970  0.7500000000000000  0.3435890335000025   F   F   F
  0.5000000000000000  0.7500000000000000  0.3435890335000025   F   F   F
  0.1666666667000030  0.7500000000000000  0.3435890335000025   F   F   F
  0.8333333332999970  0.2500000000000000  0.3435890335000025   F   F   F
  0.5000000000000000  0.2500000000000000  0.3435890335000025   F   F   F
  0.1666666667000030  0.2500000000000000  0.3435890335000025   F   F   F
  0.1581321636836910  0.2500000000000022  0.4115229918604532   T   T   T
  0.5085345040474469  0.2500000000000039  0.4115229915219757   T   T   T
  0.8333333337331013  0.2500000000000023  0.4234984188806683   T   T   T
  0.6666666667000030  0.5000000000000000  0.3054125196999991   F   F   F
  0.3333333332999970  0.5000000000000000  0.3054125196999991   F   F   F
```

0.0000000000000000	0.5000000000000000	0.3054125196999991	F	F	F
0.1580306232636400	0.7500000000000032	0.4114940229267538	T	T	T
0.6666666667000030	0.0000000000000000	0.3054125196999991	F	F	F
0.3333333332999970	0.0000000000000000	0.3054125196999991	F	F	F
0.8333333336118096	0.7499999999999990	0.2684891666103787	T	T	T
0.1574725186199167	0.7499999999999963	0.1995212030690011	T	T	T
0.0014184638875137	0.4999443132161145	0.3765112560118510	T	T	T
0.0320641752422095	0.0000099772181239	0.5279558795979111	T	T	T
0.3333333323515060	-0.0002766270161645	0.1476742887785251	T	T	T
1.0137253885084496	-0.0000615229201408	0.1618606623009100	T	T	T
0.8333333327312181	0.7499999999999931	0.0984645004844437	T	T	T
0.5174256246181821	0.7499999999999943	0.1230243368149866	T	T	T
0.1492410410094839	0.7499999999999930	0.1230243364869175	T	T	T
0.5166940703562667	0.7500000000000031	0.4878846689047373	T	T	T
0.6529412780533878	-0.0000615229210509	0.1618606624747068	T	T	T
0.8333333326334168	0.7500000000000053	0.5125993771164412	T	T	T
0.6349475476206472	0.4999055671171411	0.0829406649891738	T	T	T
0.5172522879524607	0.2499999999999944	0.1230936434294930	T	T	T
0.1494143776823135	0.2499999999999939	0.1230936431057074	T	T	T
0.0320641752422077	0.4999900227818871	0.5279558795979108	T	T	T
0.6346024896396153	0.0000099772191306	0.5279558793195396	T	T	T
0.8333333327322430	0.2499999999999941	0.0985089186147940	T	T	T
0.1499725950935556	0.7500000000000050	0.4878846692843529	T	T	T
0.3333333323515056	0.5002766270161529	0.1476742887785252	T	T	T
0.0317191175090186	0.4999055671126605	0.0829406647520537	T	T	T
0.6346024896396164	0.4999900227808767	0.5279558793195400	T	T	T
0.6529412780533876	0.5000615229210411	0.1618606624747070	T	T	T
0.8333333336770060	0.2499999999999947	0.1879312053469993	T	T	T
0.8333333326342719	0.2500000000000042	0.5125930305915755	T	T	T
0.5166642196497215	0.2500000000000038	0.4878775551173856	T	T	T
1.0137253885084478	0.5000615229201306	0.1618606623009105	T	T	T
0.1500024457989907	0.2500000000000046	0.4878775554978478	T	T	T
0.5088130151075603	0.2499999999999954	0.1993879851561120	T	T	T
0.6349475476206483	0.0000944328828460	0.0829406649891741	T	T	T
0.1578536524768763	0.2499999999999959	0.1993879848689716	T	T	T
0.3333333333055054	0.7500000000000013	0.3807266383675516	T	T	T
0.6661453261768835	0.7500000000000017	0.3765349720658385	T	T	T
0.1609265388120397	0.9999766044701898	0.4119005839032691	T	T	T
0.0336002384665591	0.7500000000000052	0.5306514408947802	T	T	T
0.6330615875528525	0.2500000000000035	0.5306465499841067	T	T	T
1.0005213410943439	0.7500000000000019	0.3765349722467575	T	T	T
0.5057401289971324	0.9999766044736511	0.4119005835221158	T	T	T
0.6544719475134221	0.7500000000000029	0.4488920873396752	T	T	T
0.1554514583048860	1.0000126733757324	0.4884698637190864	T	T	T
0.5112152072483831	1.0000126733779107	0.4884698633108403	T	T	T
0.8333333326559869	-0.0000006714435067	0.5120267760928716	T	T	T
0.1554514583048842	0.4999873266242791	0.4884698637190863	T	T	T
0.6544844835328146	0.2500000000000028	0.4488931916160079	T	T	T
0.3333333322306054	0.2500000000000044	0.4646078500250629	T	T	T
0.8333333336895230	0.9999968733898397	0.4238790131627184	T	T	T
1.0121821830978277	0.2500000000000039	0.4488931918020438	T	T	T
0.8333333326559852	0.5000006714435165	0.5120267760928712	T	T	T

0.3333333322344579	0.7500000000000027	0.4646449785869378	T	T	T
0.8333333336895217	0.5000031266101651	0.4238790131627180	T	T	T
0.5057401289971333	0.5000233955263548	0.4119005835221157	T	T	T
0.1609265388120374	0.5000233955298162	0.4119005839032686	T	T	T
0.0336050772743425	0.2500000000000055	0.5306465502624178	T	T	T
0.5112152072483837	0.4999873266220945	0.4884698633108407	T	T	T
1.0121947191129392	0.7500000000000037	0.4488920875242765	T	T	T
0.6666666667000030	0.2500000000000000	0.3054125196999991	F	F	F
0.3333333333052770	0.2500000000000013	0.3807351499070238	T	T	T
0.1606886544135221	0.5002752399922318	0.1990158502099278	T	T	T
0.8333333336603582	0.9995791540299555	0.1874931330684299	T	T	T
0.5059780132588506	-0.0002752400146982	0.1990158505374158	T	T	T
0.1606886544135233	-0.0002752399922395	0.1990158502099276	T	T	T
0.6549997214759027	0.7499999999999943	0.1621292006421090	T	T	T
0.3333333323977152	0.7499999999999938	0.1458053865913883	T	T	T
1.0116669451934335	0.7499999999999940	0.1621292004838283	T	T	T
0.6550941080380894	0.2499999999999945	0.1621346543217199	T	T	T
0.3333333324081646	0.2499999999999946	0.1460492023282573	T	T	T
1.0115725586046360	0.2499999999999954	0.1621346541654759	T	T	T
0.5059780132588506	0.5002752400146899	0.1990158505374159	T	T	T
0.8333333327502980	0.4999908706719021	0.0990547039565767	T	T	T
0.1547255324915313	0.4999157717930919	0.1224841859131427	T	T	T
0.8333333327502987	0.0000091293280853	0.0990547039565770	T	T	T
0.5119411332308818	0.0000842282010937	0.1224841862640647	T	T	T
0.1547255324915327	0.0000842282068955	0.1224841859131424	T	T	T
0.6334415099098841	0.7499999999999933	0.0802120322227159	T	T	T
0.0332251551695348	0.7499999999999936	0.0802120319849901	T	T	T
0.6333935001273122	0.2499999999999935	0.0802567338322192	T	T	T
0.0332731649577943	0.2499999999999932	0.0802567335966961	T	T	T
0.5119411332308814	0.4999157717988941	0.1224841862640646	T	T	T
0.8333333336603563	0.5004208459700370	0.1874931330684306	T	T	T
1.0000032278674871	0.2499999999999975	0.2341838889509213	T	T	T
0.3333333333183205	0.2499999999999981	0.2300722233946043	T	T	T
1.0005508251602109	0.2500000000000005	0.3765682606213260	T	T	T
0.8333333332999970	0.5000000000000000	0.3435890335000025	F	F	F
0.5000000000000000	0.5000000000000000	0.3435890335000025	F	F	F
0.1666666667000030	0.5000000000000000	0.3435890335000025	F	F	F
0.8333333332999970	0.0000000000000000	0.3435890335000025	F	F	F
0.5000000000000000	0.0000000000000000	0.3435890335000025	F	F	F
0.1666666667000030	0.0000000000000000	0.3435890335000025	F	F	F
0.6666666667000030	0.7500000000000000	0.3054125196999991	F	F	F
0.3333333332999970	0.7500000000000000	0.3054125196999991	F	F	F
0.0000000000000000	0.7500000000000000	0.3054125196999991	F	F	F
0.3333333332999970	0.2500000000000000	0.3054125196999991	F	F	F
0.0000000000000000	0.2500000000000000	0.3054125196999991	F	F	F
0.8333333332999970	0.5000000000000000	0.2672360059000027	F	F	F
0.5000000000000000	0.5000000000000000	0.2672360059000027	F	F	F
0.1666666667000030	0.5000000000000000	0.2672360059000027	F	F	F
0.8333333332999970	0.0000000000000000	0.2672360059000027	F	F	F
0.5000000000000000	0.0000000000000000	0.2672360059000027	F	F	F
0.1666666667000030	0.0000000000000000	0.2672360059000027	F	F	F
0.6667852254251921	0.7499999999999987	0.2345302139543776	T	T	T

```
 0.3333333333444125  0.7499999999999976  0.2300725472294216   T   T   T
 0.9998814419509138  0.7499999999999987  0.2345302137796589   T   T   T
 0.6666634393198798  0.2499999999999982  0.2341838891069266   T   T   T
 0.6661158421086144  0.2500000000000017  0.3765682604349987   T   T   T
 0.6330664263600504  0.7500000000000041  0.5306514406169611   T   T   T

110_stoich/CONTCAR
Mg O
   1.00857736950000
     8.9333042307999992    0.0000000000000000    0.0000000000000000
     0.0000000000000000    8.4223999999999997    0.0000000000000000
     0.0000000000000000    0.0000000000000000   39.0000000000000000
   Mg   O
    78    78
Selective dynamics
Direct
-0.0003645695215041  0.0001566605348547  0.0933454324200900   T   T   T
 0.5001197895453393  0.7500000000000013  0.4140860779026534   T   T   T
 0.8333333333418564  0.7499999999999983  0.4141442134602440   T   T   T
 1.0000439730962758 -0.0000290367532396  0.4474264582157146   T   T   T
 0.8333333332999970  0.2500000000000000  0.2672360059000027   F   F   F
 0.5000000000000000  0.2500000000000000  0.2672360059000027   F   F   F
 0.1666666667000030  0.2500000000000000  0.2672360059000027   F   F   F
 0.3333333333363142 -0.0002419776419571  0.4473816471066181   T   T   T
 0.6666226935920342 -0.0000290367537274  0.4474264582208962   T   T   T
 1.0000439730962771  0.5000290367532405  0.4474264582157143   T   T   T
 0.6667917122872632  0.5019865489177743  0.2332154141602802   T   T   T
 0.3333333333457102  0.4994079761575544  0.2330013954382371   T   T   T
-0.0001250456357938  0.5019865489073433  0.2332154141641443   T   T   T
 0.3333333333363139  0.5002419776419548  0.4473816471066180   T   T   T
 0.6666226935920317  0.5000290367537306  0.4474264582208962   T   T   T
 0.3333333333491263  0.5000666683051729  0.3777697511984302   T   T   T
 0.6667917122872624 -0.0019865489177728  0.2332154141602800   T   T   T
 0.3333333333457101  1.0005920238424462  0.2330013954382368   T   T   T
-0.0001250456357925 -0.0019865489073413  0.2332154141641442   T   T   T
 0.8333333333366586  0.7500000000000019  0.1975957191172766   T   T   T
 0.1666666667000030  0.7500000000000000  0.2672360059000027   F   F   F
 0.4992802138696915  0.7499999999999989  0.1968130832433984   T   T   T
 0.5000000000000000  0.7500000000000000  0.2672360059000027   F   F   F
 0.0000000000000000  0.0000000000000000  0.3054125196999991   F   F   F
 0.6666755506805798  0.4998124594829383  0.3778128412554506   T   T   T
 0.6666755506805803  1.0001875405170615  0.3778128412554506   T   T   T
 0.3333333333491262  0.9999333316948303  0.3777697511984303   T   T   T
 0.9999911159756334  1.0001875405171139  0.3778128412505832   T   T   T
 0.8333333332999970  0.7500000000000000  0.3435890335000025   F   F   F
 0.5000000000000000  0.7500000000000000  0.3435890335000025   F   F   F
 0.1666666667000030  0.7500000000000000  0.3435890335000025   F   F   F
 0.8333333332999970  0.2500000000000000  0.3435890335000025   F   F   F
 0.5000000000000000  0.2500000000000000  0.3435890335000025   F   F   F
 0.1666666667000030  0.2500000000000000  0.3435890335000025   F   F   F
 0.1667366539671207  0.2499999999999999  0.4141310879733288   T   T   T
 0.4999300127134121  0.2500000000000002  0.4141310879778312   T   T   T
```

0.8333333333416268	0.2500000000000012	0.4141936497722772	T	T	T
0.6666666667000030	0.5000000000000000	0.3054125196999991	F	F	F
0.3333333332999970	0.5000000000000000	0.3054125196999991	F	F	F
0.0000000000000000	0.5000000000000000	0.3054125196999991	F	F	F
0.1665468771347330	0.7500000000000007	0.4140860778980774	T	T	T
0.6666666667000030	0.0000000000000000	0.3054125196999991	F	F	F
0.3333333332999970	0.0000000000000000	0.3054125196999991	F	F	F
0.8333333333388350	0.7500000000000020	0.2697645684497140	T	T	T
0.1673864527947485	0.7499999999999991	0.1968130832462567	T	T	T
0.9999911159756343	0.4998124594828862	0.3778128412505830	T	T	T
0.0000407597061390	-0.0000069984847556	0.5178629812383325	T	T	T
0.3333333333275848	0.0007211624762884	0.1633906970854529	T	T	T
-0.0003568988591837	-0.0018922634785564	0.1637267793054361	T	T	T
0.8333333333342521	0.7500000000000000	0.1238819495197387	T	T	T
0.4992265153109715	0.7499999999999982	0.1235061565349841	T	T	T
0.1674401513627376	0.7500000000000001	0.1235061565389031	T	T	T
0.5000445751785015	0.7500000000000012	0.4873337970659868	T	T	T
0.6670235655273705	-0.0018922634919708	0.1637267793012061	T	T	T
0.8333333333190540	0.7499999999999988	0.4873619611012240	T	T	T
0.6670312361958080	0.4998433394691084	0.0933454324159539	T	T	T
0.5000302486924461	0.2500000000000008	0.1236729295851634	T	T	T
0.1666364179911081	0.2499999999999982	0.1236729295878055	T	T	T
0.3333333333240900	1.0000143669422743	0.5178209585411345	T	T	T
0.0000407597061408	0.5000069984847553	0.5178629812383320	T	T	T
0.6666259069459822	-0.0000069984847822	0.5178629812435822	T	T	T
0.8333333333289670	0.2499999999999992	0.1242896338001883	T	T	T
0.3333333333355020	0.4995682568796786	0.0929620255131203	T	T	T
0.1666220914789917	0.7500000000000016	0.4873337970617845	T	T	T
0.3333333333355032	0.0004317431203179	0.0929620255131202	T	T	T
0.3333333333275835	0.4992788375237119	0.1633906970854529	T	T	T
-0.0003645695215039	0.4998433394651446	0.0933454324200901	T	T	T
0.3333333333240904	0.4999856330577266	0.5178209585411336	T	T	T
0.6666259069459821	0.5000069984847807	0.5178629812435822	T	T	T
0.6670235655273725	0.5018922634919688	0.1637267793012064	T	T	T
0.8333333333259437	0.2499999999999994	0.1970947633956228	T	T	T
0.8333333333188818	0.2500000000000008	0.4873829737876947	T	T	T
0.5000238334164058	0.2499999999999987	0.4873056330084010	T	T	T
-0.0003568988591850	0.5018922634785569	0.1637267793054360	T	T	T
0.1666428332412107	0.2499999999999993	0.4873056330043073	T	T	T
0.5008694483143252	0.2500000000000009	0.1967974720378655	T	T	T
0.6670312361958068	0.0001566605308914	0.0933454324159538	T	T	T
0.1657972183459869	0.2500000000000022	0.1967974720422105	T	T	T
0.3333333333489433	0.7500000000000006	0.3778437672608087	T	T	T
0.6668019656183614	0.7500000000000006	0.3778518193618455	T	T	T
0.1666427354015242	-0.0000733601760784	0.4141126565147830	T	T	T
1.0000221493482964	0.7499999999999983	0.5193371989743790	T	T	T
0.6665973852007302	0.2499999999999997	0.5193363046263574	T	T	T
-0.0001352989611656	0.7500000000000010	0.3778518193573331	T	T	T
0.5000239312773653	-0.0000733601763985	0.4141126565200221	T	T	T
0.6666900583350903	0.7499999999999984	0.4477274692625883	T	T	T
0.3333333333241826	0.2500000000000005	0.5192838149169268	T	T	T
0.1666301066957436	-0.0000911107710878	0.4868643251447923	T	T	T

0.5000365599623858	-0.0000911107712943	0.4868643251486740	T	T	T
0.8333333333188082	-0.0000066955667207	0.4869124393590413	T	T	T
0.1666301066957448	0.5000911107710869	0.4868643251447913	T	T	T
0.6665509216700081	0.2500000000000005	0.4477636999710178	T	T	T
0.3333333333364036	0.2500000000000000	0.4477194980638146	T	T	T
0.8333333333409750	0.0001624419261294	0.4141558822694694	T	T	T
0.0001157450174199	0.2499999999999997	0.4477636999664059	T	T	T
0.8333333333188080	0.5000066955667195	0.4869124393590415	T	T	T
0.3333333333360349	0.7500000000000024	0.4476821219229609	T	T	T
0.8333333333409746	0.4998375580738718	0.4141558822694694	T	T	T
0.5000239312773641	0.5000733601764001	0.4141126565200219	T	T	T
0.1666427354015251	0.5000733601760794	0.4141126565147829	T	T	T
0.0000692814529294	0.2499999999999997	0.5193363046209585	T	T	T
0.5000365599623865	0.5000911107712952	0.4868643251486733	T	T	T
0.9999766083526265	0.7499999999999989	0.4477274692579850	T	T	T
0.6666666667000030	0.2500000000000000	0.3054125196999991	F	F	F
0.3333333333492269	0.2499999999999993	0.3778802494590792	T	T	T
0.1667715997331632	0.5004868872349499	0.1967505082268340	T	T	T
0.8333333333332611	-0.0024496571803737	0.1970874289370694	T	T	T
0.4998950669285827	-0.0004868872395949	0.1967505082222405	T	T	T
0.1667715997331651	-0.0004868872349491	0.1967505082268341	T	T	T
0.6665877283225952	0.7499999999999996	0.1635194623050242	T	T	T
0.3333333333291146	0.7499999999999986	0.1629965302832793	T	T	T
0.0000789383513024	0.7500000000000009	0.1635194623074402	T	T	T
0.6675617345914168	0.2500000000000008	0.1634114148451077	T	T	T
0.3333333333280177	0.2500000000000012	0.1632235329972880	T	T	T
-0.0008950679305314	0.2499999999999985	0.1634114148502198	T	T	T
0.4998950669285834	0.5004868872395964	0.1967505082222407	T	T	T
0.8333333333312708	0.5009406985006626	0.1244707869473369	T	T	T
0.1670172142094755	0.4998938459047489	0.1240348811119952	T	T	T
0.8333333333312691	-0.0009406985006629	0.1244707869473367	T	T	T
0.4996494524689572	0.0001061540878708	0.1240348811089175	T	T	T
0.1670172142094766	0.0001061540952494	0.1240348811119950	T	T	T
0.6668141597900490	0.7499999999999990	0.0918304434766956	T	T	T
0.3333333333322807	0.7499999999999981	0.0914002664622536	T	T	T
-0.0001474931145826	0.7500000000000001	0.0918304434807067	T	T	T
0.6673208466778687	0.2500000000000000	0.0919764163428114	T	T	T
0.3333333333375151	0.2499999999999985	0.0915802598368964	T	T	T
0.9993458199959018	0.2499999999999986	0.0919764163473950	T	T	T
0.4996494524689563	0.4998938459121270	0.1240348811089172	T	T	T
0.8333333333332614	0.5024496571803734	0.1970874289370697	T	T	T
-0.0001251966916711	0.2500000000000000	0.2329685424623276	T	T	T
0.3333333333445171	0.2500000000000014	0.2329375087435451	T	T	T
-0.0000069797562878	0.2500000000000011	0.3779030339838140	T	T	T
0.8333333332999970	0.5000000000000000	0.3435890335000025	F	F	F
0.5000000000000000	0.5000000000000000	0.3435890335000025	F	F	F
0.1666666667000030	0.5000000000000000	0.3435890335000025	F	F	F
0.8333333332999970	0.0000000000000000	0.3435890335000025	F	F	F
0.5000000000000000	0.0000000000000000	0.3435890335000025	F	F	F
0.1666666667000030	0.0000000000000000	0.3435890335000025	F	F	F
0.6666666667000030	0.7500000000000000	0.3054125196999991	F	F	F
0.3333333332999970	0.7500000000000000	0.3054125196999991	F	F	F

```
 0.0000000000000000  0.7500000000000000  0.3054125196999991   F   F   F
 0.3333333333240645  0.7500000000000001  0.5193033815465280   T   T   T
 0.3333333332999970  0.2500000000000000  0.3054125196999991   F   F   F
 0.0000000000000000  0.2500000000000000  0.3054125196999991   F   F   F
 0.8333333332999970  0.5000000000000000  0.2672360059000027   F   F   F
 0.5000000000000000  0.5000000000000000  0.2672360059000027   F   F   F
 0.1666666667000030  0.5000000000000000  0.2672360059000027   F   F   F
 0.8333333332999970  0.0000000000000000  0.2672360059000027   F   F   F
 0.5000000000000000  0.0000000000000000  0.2672360059000027   F   F   F
 0.1666666667000030  0.0000000000000000  0.2672360059000027   F   F   F
 0.6664788231967095  0.7500000000000016  0.2336454338356298   T   T   T
 0.3333333333458431  0.7499999999999991  0.2328831571106472   T   T   T
 0.0001878434698371  0.7500000000000013  0.2336454338387827   T   T   T
 0.6667918633391658  0.2499999999999996  0.2329685424581233   T   T   T
 0.6666736464133304  0.2500000000000004  0.3779030339880624   T   T   T
 0.6666445173055219  0.7499999999999999  0.5193371989797899   T   T   T

111_2x2_octo/CONTCAR
Mg O
   1.00857736950000
     5.9555361538999998    0.0000000000000000    0.0000000000000000
    -2.9777680768999999    5.1576456023999997    0.0000000000000000
     0.0000000000000000    0.0000000000000000   39.1760745361000033
   Mg   O
    34    34
Selective dynamics
Direct
 0.8333333332999970  0.6666666667000030  0.3723698057999982   F   F   F
 0.9983139693728155  0.4991569847144978  0.3155554319205349   T   T   T
 0.5008430152743787  0.4991569847204735  0.3155554319203608   T   T   T
 0.3333333332999970  0.1666666667000030  0.3723698057999982   F   F   F
 0.8333333332999970  0.1666666667000030  0.3723698057999982   F   F   F
 0.3333333332999970  0.6666666667000030  0.3723698057999982   F   F   F
 0.1666666667000030  0.3333333332999970  0.4344315995000017   F   F   F
 0.6666666667000030  0.3333333332999970  0.4344315995000017   F   F   F
 0.1666666667000030  0.8333333332999970  0.4344315995000017   F   F   F
 0.6666666667000030  0.8333333332999970  0.4344315995000017   F   F   F
 0.0000000000203495 -0.0000000000126116  0.4920427040124866   T   T   T
 0.5008430152787372  0.0016860306188039  0.3155554319199498   T   T   T
 0.4991569847231942  0.9983139693850631  0.4912459733671319   T   T   T
 0.4991569847275540  0.5008430152775972  0.4912459733667210   T   T   T
 0.3426700415105651  0.1713350207828748  0.5489944182615225   T   T   T
 0.8286649792361674  0.1713350207768400  0.5489944182634525   T   T   T
 0.3333333333272888  0.6666666666839159  0.5474168919900513   T   T   T
 0.8286649792248793  0.6573299584919348  0.5489944182604684   T   T   T
 0.1603698695455216  0.3207397391106261  0.6035787591291382   T   T   T
 0.6666666666498758  0.3333333332866695  0.6192971780422196   T   T   T
 0.1603698695418927  0.8396301304326887  0.6035787591266594   T   T   T
 0.6792602608698776  0.8396301304320196  0.6035787591285500   T   T   T
-0.0000000000108129 -0.0000000000364493  0.6576973713795914   T   T   T
 0.0016860306233178  0.5008430152835704  0.4912459733665469   T   T   T
-0.0000000000203487  0.0000000000126133  0.3147587012768822   T   T   T
```

```
  0.8396301304590379  0.1603698695663770  0.2032226461508222   T   T   T
  0.3207397391282614  0.1603698695670449  0.2032226461489319   T   T   T
  0.3333333333501211  0.6666666667133228  0.1875042272383484   T   T   T
  0.8396301304554061  0.6792602608912304  0.2032226461483438   T   T   T
  0.0000000000108107  0.0000000000364416  0.1491040338977437   T   T   T
  0.1713350207703547  0.3426700414985298  0.2578069870207226   T   T   T
  0.6666666666727104  0.3333333333160818  0.2593845132885887   T   T   T
  0.6573299584989659  0.8286649792218891  0.2578069870196679   T   T   T
  0.1713350207590655  0.8286649792279217  0.2578069870177380   T   T   T
  0.3147766525387290  0.1573883262558006  0.6346220890769737   T   T   T
  0.6665438070223410  0.8332719035041217  0.5192389019299996   T   T   T
  0.1573883262838391  0.8426116737464212  0.1721793161975332   T   T   T
  0.1573883262946202  0.3147766525860945  0.1721793162017559   T   T   T
  0.8426116737064487  0.6852233474160362  0.6346220890750859   T   T   T
  0.4886389639382379  0.9772779278967932  0.5784092700801475   T   T   T
  0.0227220721223287  0.5113610360617868  0.5784092700764658   T   T   T
  0.4886389638993925  0.5113610360815334  0.5784092700754169   T   T   T
  0.1667280965036655  0.8332719035057609  0.5192389019292234   T   T   T
  0.8426116737172263  0.1573883262525027  0.6346220890793088   T   T   T
 -0.0000000000130940  0.0000000000011910  0.5731242661649061   T   T   T
  0.6666666666659078  0.3333333333353057  0.5216003031466285   T   T   T
  0.3333333333340918  0.6666666666646933  0.2852011021265412   T   T   T
  0.6852233474591315  0.8426116737431265  0.1721793161998681   T   T   T
  0.8332719034974753  0.1667280964930970  0.2875625033450744   T   T   T
  0.3334561929753732  0.1667280964947371  0.2875625033442977   T   T   T
  0.1674396201018478  0.3348792402997923  0.3440663374912380   T   T   T
  0.6666666666338360  0.3333333333628206  0.3444766657896501   T   T   T
  0.1674396201009789  0.8325603798955399  0.3440663374906060   T   T   T
  0.6651207596968004  0.8325603798947081  0.3440663374913699   T   T   T
  0.5113610361011013  0.4886389639179665  0.2283921351998552   T   T   T
  0.0000000000000000  0.0000000000000000  0.4034007026000026   F   F   F
  0.5000000000000000  0.0000000000000000  0.4034007026000026   F   F   F
  0.0000000000000000  0.5000000000000000  0.4034007026000026   F   F   F
  0.5000000000000000  0.5000000000000000  0.4034007026000026   F   F   F
  0.9772779278766787  0.4886389639377133  0.2283921351988059   T   T   T
  0.5113610360622601  0.0227220721041966  0.2283921351951247   T   T   T
  0.0000000000130925 -0.0000000000011942  0.2336771391118358   T   T   T
  0.3348792403046169  0.1674396201060004  0.4627350677745065   T   T   T
  0.8325603798983116  0.1674396201051705  0.4627350677752701   T   T   T
  0.3333333333661630  0.6666666666371820  0.4623247394766185   T   T   T
  0.8325603798974425  0.6651207596987915  0.4627350677746386   T   T   T
  0.8332719034953736  0.6665438070142724  0.2875625033449917   T   T   T
  0.1667280965057701  0.3334561929880132  0.5192389019293056   T   T   T

111_stoich/CONTCAR
Mg O
   1.00857736950000
     5.9555361538999998    0.0000000000000000    0.0000000000000000
    -2.9777680768999999    5.1576456023999997    0.0000000000000000
     0.0000000000000000    0.0000000000000000   39.1760745361000033
   Mg   O
    40    40
```

Selective dynamics
Direct

0.8333333333193380	0.6666666666666643	0.3723698058251728	F	F	F
0.9999999999916014	0.5000000000000000	0.3103080121822259	F	F	F
0.5000000000000000	0.5000000000000000	0.3103080121822259	F	F	F
0.3333333333361352	0.1666666666666643	0.3723698058251728	F	F	F
0.8333333333277366	0.1666666666666643	0.3723698058251728	F	F	F
0.3333333333277366	0.6666666666666643	0.3723698058251728	F	F	F
0.9999999998354294	0.4999999998968918	0.6637019468611846	T	T	T
0.1666666666638648	0.3333333333333357	0.4344315994706704	F	F	F
0.6666666666554733	0.3333333333333357	0.4344315994706704	F	F	F
0.1666666666554733	0.8333333333333357	0.4344315994706704	F	F	F
0.6666666666638719	0.8333333333333357	0.4344315994706704	F	F	F
0.0000000000026918	0.0000000000053456	0.4930769565870575	T	T	T
0.4999999999916014	0.0000000000000000	0.3103080121822259	F	F	F
0.5000000000039364	0.0000000000038370	0.4930769565870495	T	T	T
0.5000000000002610	0.5000000000041411	0.4930769565872583	T	T	T
0.3333333333701148	0.1666666666905585	0.5497852089638960	T	T	T
0.8333333333759583	0.1666666666972043	0.5497852089637314	T	T	T
0.3333333333711668	0.6666666666948521	0.5497852089641098	T	T	T
0.8333333333685530	0.6666666666929547	0.5497852089635256	T	T	T
0.1666666667752699	0.3333333334039509	0.6053160412563285	T	T	T
0.6666666667590984	0.3333333333908890	0.6053160412552652	T	T	T
0.1666666667499488	0.8333333333863329	0.6053160412560384	T	T	T
0.6666666667722265	0.8333333334085720	0.6053160412555539	T	T	T
-0.0000000001515623	-0.0000000000872147	0.6637019468614584	T	T	T
0.4999999998338867	-0.0000000001000001	0.6637019468606351	T	T	T
1.0000000000011451	0.5000000000050990	0.4930769565868498	T	T	T
0.0000000000000000	0.0000000000000000	0.3103080121822259	F	F	F
0.4999999998546462	0.4999999999158855	0.6637019468609074	T	T	T
0.8333333333288988	0.1666666666658723	0.1952503004614064	T	T	T
0.3333333333290448	0.1666666666663506	0.1952503004614692	T	T	T
0.3333333333281812	0.6666666666655118	0.1952503004614049	T	T	T
0.5000000000303617	0.5000000000197763	0.1348022665426316	T	T	T
0.8333333333281213	0.6666666666667107	0.1952503004614710	T	T	T
0.5000000000302569	0.0000000000195592	0.1348022665427281	T	T	T
0.0000000000312603	0.0000000000202300	0.1348022665426528	T	T	T
0.1666666666522639	0.3333333333289495	0.2530536810789117	T	T	T
0.6666666666526602	0.3333333333279718	0.2530536810785753	T	T	T
1.0000000000293947	0.5000000000200118	0.1348022665427495	T	T	T
0.6666666666521226	0.8333333333290794	0.2530536810789215	T	T	T
0.1666666666524046	0.8333333333278422	0.2530536810785655	T	T	T
0.3333333331735806	0.1666666665784318	0.6451759161657851	T	T	T
0.8333333333619624	0.1666666666855290	0.1151230637111265	T	T	T
0.6666666666902062	0.8333333333520508	0.5229748493510812	T	T	T
0.3333333333611101	0.6666666666850990	0.1151230637111113	T	T	T
0.1666666666643749	0.8333333333346058	0.1701370408362378	T	T	T
0.6666666666654379	0.3333333333351406	0.1701370408362193	T	T	T
0.1666666666651518	0.3333333333335184	0.1701370408362196	T	T	T
0.8333333331815574	0.6666666665778024	0.6451759161651269	T	T	T
0.5000000000740855	0.0000000000498081	0.5816189840735166	T	T	T
1.0000000000812064	0.5000000000490291	0.5816189840732635	T	T	T

0.5000000000893616 0.5000000000615492 0.5816189840733738 T T T
0.1666666666883856 0.8333333333504065 0.5229748493507076 T T T
0.3333333331599238 0.6666666665586165 0.6451759161660707 T T T
0.8333333331586394 0.1666666665579621 0.6451759161654130 T T T
0.8333333333617943 0.6666666666859984 0.1151230637108673 T T T
0.0000000000877169 0.0000000000606982 0.5816189840731230 T T T
0.6666666666907378 0.3333333333515488 0.5229748493510493 T T T
0.3333333333231355 0.6666666666644163 0.2825428206504203 T T T
0.6666666666641397 0.8333333333340525 0.1701370408362011 T T T
0.8333333333224645 0.1666666666640826 0.2825428206504542 T T T
0.3333333333247699 0.1666666666640647 0.2825428206501187 T T T
0.1666666666638648 0.3333333333333357 0.3413389090062537 F F F
0.6666666666554733 0.3333333333333357 0.3413389090062537 F F F
0.1666666666554733 0.8333333333333357 0.3413389090062537 F F F
0.6666666666638719 0.8333333333333357 0.3413389090062537 F F F
0.4999999999859973 0.4999999999954136 0.2256477431981825 T T T
0.0000000000000000 0.0000000000000000 0.4034007026491935 F F F
0.4999999999916014 0.0000000000000000 0.4034007026491935 F F F
0.9999999999916014 0.5000000000000000 0.4034007026491935 F F F
0.5000000000000000 0.5000000000000000 0.4034007026491935 F F F
0.9999999999863629 0.4999999999949069 0.2256477431979594 T T T
0.4999999999863743 -0.0000000000050658 0.2256477431979383 T T T
-0.0000000000140558 -0.0000000000046141 0.2256477431982033 T T T
0.3333333333361352 0.1666666666666643 0.4654624962921403 F F F
0.8333333333277366 0.1666666666666643 0.4654624962921403 F F F
0.3333333333277366 0.6666666666666643 0.4654624962921403 F F F
0.8333333333193380 0.6666666666666643 0.4654624962921403 F F F
0.8333333333247988 0.6666666666637289 0.2825428206501527 T T T
0.1666666666898666 0.3333333333508749 0.5229748493507479 T T T
0.3333333333625301 0.1666666666855688 0.1151230637108520 T T T

FFbulk:

100_1x1/CONTCAR

Mg O
1.00857736950000
2.9777680768999999 0.0000000000000000 0.0000000000000000
0.0000000000000000 2.9777680768999999 0.0000000000000000
0.0000000000000000 0.0000000000000000 64.7455000000000069
Mg O
20 20
Selective dynamics
Direct
0.4999999999999910 0.5000000000000043 0.2614263895494847 T T T
0.0000000000000000 0.0000000000000000 0.4227753280000002 F F F
0.5000000000000000 0.5000000000000000 0.3902541488999987 F F F
0.5000000000000191 0.5000000000000151 0.5184348564813578 T T T
-0.0000000000000055 -0.0000000000000013 0.6776433676871836 T T T
0.0000000000000000 0.0000000000000000 0.3577329698999989 F F F
0.5000000000000000 0.5000000000000000 0.3252117907999974 F F F
0.0000000000000358 0.0000000000000102 0.5503097345148251 T T T

```
-0.0000000000000063  0.0000000000000133  0.2933136879542897   T   T   T
 0.4999999999999806  0.5000000000000075  0.4546734308464080   T   T   T
 0.5000000000000048  0.4999999999999876  0.5822028817182564   T   T   T
-0.0000000000000103 -0.0000000000000026  0.4865607292521791   T   T   T
 0.4999999999999941  0.5000000000000054  0.1976773842884055   T   T   T
 0.0000000000000224  0.0000000000000002  0.6141156939595941   T   T   T
-0.0000000000000168 -0.0000000000000086  0.1657842370846329   T   T   T
 0.5000000000000027  0.4999999999999706  0.1338714248422423   T   T   T
 0.4999999999999849  0.4999999999999895  0.6459163350415666   T   T   T
-0.0000000000000144 -0.0000000000000238  0.1020707837591838   T   T   T
 0.5000000000000100  0.5000000000000041  0.0703437511128874   T   T   T
 0.0000000000000093  0.0000000000000138  0.2295522623213343   T   T   T
 0.0000000000000105 -0.0000000000000048  0.5822229191949282   T   T   T
 0.5000000000000332  0.5000000000000120  0.5503189824199342   T   T   T
 0.5000000000000219  0.4999999999999987  0.6141674541192185   T   T   T
-0.0000000000000137 -0.0000000000000122  0.6460033978311128   T   T   T
 0.0000000000000104  0.0000000000000196  0.5184383619831171   T   T   T
 0.4999999999999926  0.5000000000000004  0.4865616230866217   T   T   T
 0.0000000000000090  0.0000000000000028  0.0699564032545642   T   T   T
 0.5000000000000000  0.5000000000000000  0.3577329698999989   F   F   F
 0.5000000000000000  0.5000000000000000  0.4227753280000002   F   F   F
 0.0000000000000000  0.0000000000000000  0.3902541488999987   F   F   F
 0.0000000000000000  0.0000000000000000  0.3252117907999974   F   F   F
 0.4999999999999934  0.5000000000000085  0.2933146400693849   T   T   T
-0.0000000000000084  0.0000000000000036  0.2614254957150399   T   T   T
 0.5000000000000062  0.5000000000000100  0.2295487568195628   T   T   T
-0.0000000000000047  0.0000000000000075  0.1976681363832748   T   T   T
 0.4999999999999856  0.4999999999999890  0.1657641996079316   T   T   T
-0.0000000000000003 -0.0000000000000257  0.1338196646825950   T   T   T
 0.4999999999999896  0.4999999999999747  0.1019837209695934   T   T   T
-0.0000000000000151  0.0000000000000078  0.4546724787313171   T   T   T
 0.4999999999999961  0.4999999999999981  0.6780307155454999   T   T   T
```

110_fac/CONTCAR

```
Mg O
  1.00857736950000
    8.9333042307999992    0.0000000000000000    0.0000000000000000
    0.0000000000000000    8.4223999999999997    0.0000000000000000
    0.0000000000000000    0.0000000000000000   39.0000000000000000
  Mg   O
   74   74
Selective dynamics
Direct
 0.0100028564772613 -0.0000530374814904  0.0844678620662170   T   T   T
 0.4999434898060368  0.7500000000000016  0.4186410722771244   T   T   T
 0.8333333333353737  0.7500000000000007  0.4184670792651597   T   T   T
 0.9982788404197203 -0.0000012361362713  0.4550928133134768   T   T   T
 0.8333333332999970  0.2500000000000000  0.2672360059000027   F   F   F
 0.5000000000000000  0.2500000000000000  0.2672360059000027   F   F   F
 0.1666666667000030  0.2500000000000000  0.2672360059000027   F   F   F
 0.3333333333340263 -0.0000011409399800  0.4586328547168579   T   T   T
```

0.6683878262510918	-0.0000012361363128	0.4550928133147945	T	T	T
0.9982788404197175	0.5000012361362730	0.4550928133134773	T	T	T
0.6660468605468977	0.5001529488586818	0.2299971285661754	T	T	T
0.3333333333440669	0.4999913323107565	0.2294406253836192	T	T	T
0.0006198061166438	0.5001529488512567	0.2299971285685216	T	T	T
0.3333333333340253	0.5000011409399835	0.4586328547168580	T	T	T
0.6683878262510896	0.5000012361363154	0.4550928133147948	T	T	T
0.3333333333420466	0.4999972944418109	0.3814032897738870	T	T	T
0.6660468605468993	0.9998470511413168	0.2299971285661751	T	T	T
0.3333333333440678	1.0000086676892457	0.2294406253836193	T	T	T
0.0006198061166431	0.9998470511487446	0.2299971285685216	T	T	T
0.8333333333373456	0.7499999999999982	0.1925019893408584	T	T	T
0.1666666667000030	0.7500000000000000	0.2672360059000027	F	F	F
0.5000002319398529	0.7499999999999997	0.1922367339573948	T	T	T
0.5000000000000000	0.7500000000000000	0.2672360059000027	F	F	F
0.0000000000000000	0.0000000000000000	0.3054125196999991	F	F	F
0.6659635626842958	0.4999895390625374	0.3808679999076424	T	T	T
0.6659635626842950	0.0000104609374646	0.3808679999076420	T	T	T
0.3333333333420469	1.0000027055581906	0.3814032897738872	T	T	T
0.0007031039738099	0.0000104609378385	0.3808679999043320	T	T	T
0.8333333332999970	0.7500000000000000	0.3435890335000025	F	F	F
0.5000000000000000	0.7500000000000000	0.3435890335000025	F	F	F
0.1666666667000030	0.7500000000000000	0.3435890335000025	F	F	F
0.8333333332999970	0.2500000000000000	0.3435890335000025	F	F	F
0.5000000000000000	0.2500000000000000	0.3435890335000025	F	F	F
0.1666666667000030	0.2500000000000000	0.3435890335000025	F	F	F
0.1667239768949261	0.2500000000000012	0.4186432543098482	T	T	T
0.4999426897709573	0.2500000000000007	0.4186432543119338	T	T	T
0.8333333333352259	0.2499999999999985	0.4184715039941565	T	T	T
0.6666666667000030	0.5000000000000000	0.3054125196999991	F	F	F
0.3333333332999970	0.5000000000000000	0.3054125196999991	F	F	F
0.0000000000000000	0.5000000000000000	0.3054125196999991	F	F	F
0.1667231768596515	0.7500000000000007	0.4186410722749656	T	T	T
0.6666666667000030	0.0000000000000000	0.3054125196999991	F	F	F
0.3333333332999970	0.0000000000000000	0.3054125196999991	F	F	F
0.8333333333384275	0.7500000000000010	0.2677456354351283	T	T	T
0.1666664347343054	0.7499999999999998	0.1922367339589002	T	T	T
0.0007031039738098	0.4999895390621621	0.3808679999043321	T	T	T
0.0100190259887642	-0.0000043306433556	0.5264162729917619	T	T	T
0.3333333333368368	-0.0000087744833133	0.1522413055174762	T	T	T
0.9982657481049334	-0.0000244888298395	0.1557843020598345	T	T	T
0.8333333333371290	0.7499999999999987	0.1145954427412762	T	T	T
0.5099673270399032	0.7500000000000012	0.1189338343675133	T	T	T
0.1566993396338161	0.7500000000000009	0.1189338343682804	T	T	T
0.5099648348272872	0.7500000000000000	0.4919544274162211	T	T	T
0.6684009185708042	-0.0000244888297800	0.1557843020587280	T	T	T
0.8333333333343075	0.7500000000000004	0.4963146371267860	T	T	T
0.6566638101975603	0.5000530374815049	0.0844678620657346	T	T	T
0.5099680639815017	0.2500000000000007	0.1189177232618269	T	T	T
0.1566986026921166	0.2499999999999978	0.1189177232625881	T	T	T
0.0100190259887627	0.5000043306433568	0.5264162729917619	T	T	T
0.6566476406806377	-0.0000043306432939	0.5264162729923063	T	T	T

0.8333333333369642	0.2499999999999986	0.1145591447748570	T	T	T
0.1567018318411497	0.7500000000000012	0.4919544274153881	T	T	T
0.3333333333368353	0.5000087744833115	0.1522413055174764	T	T	T
0.0100028564772641	0.5000530374814881	0.0844678620662162	T	T	T
0.6566476406806387	0.5000043306432904	0.5264162729923062	T	T	T
0.6684009185708069	0.5000244888297799	0.1557843020587286	T	T	T
0.8333333333363917	0.2499999999999988	0.1923956372490692	T	T	T
0.8333333333343625	0.2500000000000003	0.4963170383957806	T	T	T
0.5099652517528662	0.2500000000000013	0.4919559520140577	T	T	T
0.9982657481049356	0.5000244888298380	0.1557843020598340	T	T	T
0.1567014149155706	0.2499999999999993	0.4919559520132584	T	T	T
0.4999875911929603	0.2500000000000001	0.1922211933321089	T	T	T
0.6566638101975580	-0.0000530374815050	0.0844678620657337	T	T	T
0.1666790754787452	0.2500000000000002	0.1922211933336807	T	T	T
0.3333333333400973	0.7499999999999998	0.3815354723134786	T	T	T
0.6663399371744015	0.7500000000000009	0.3809896168091810	T	T	T
0.1677491734065516	0.9999981848944335	0.4188267060377518	T	T	T
0.0099854853726552	0.7500000000000017	0.5292816351129663	T	T	T
0.6566784275456826	0.2499999999999999	0.5292843097608723	T	T	T
1.0003267294857059	0.7499999999999994	0.3809896168057714	T	T	T
0.4989174932612084	0.9999981848944749	0.4188267060397237	T	T	T
0.6704019186191479	0.7500000000000011	0.4556693226584313	T	T	T
0.1613796180431583	0.9999985438796219	0.4917168902418918	T	T	T
0.5052870486247026	0.9999985438796444	0.4917168902426899	T	T	T
0.8333333333344217	-0.0000013202188265	0.4954126723246920	T	T	T
0.1613796180431572	0.5000014561203820	0.4917168902418920	T	T	T
0.6704024991528832	0.2500000000000003	0.4556720942360001	T	T	T
0.3333333333343914	0.2500000000000012	0.4590498931126586	T	T	T
0.8333333333340240	0.9999960088249703	0.4185679314077780	T	T	T
0.9962641675171229	0.2500000000000017	0.4556720942350940	T	T	T
0.8333333333344228	0.5000013202188271	0.4954126723246924	T	T	T
0.3333333333344204	0.7500000000000013	0.4590489469434717	T	T	T
0.8333333333340238	0.5000039911750308	0.4185679314077784	T	T	T
0.4989174932612097	0.5000018151055265	0.4188267060397242	T	T	T
0.1677491734065509	0.5000018151055671	0.4188267060377519	T	T	T
0.0099882391241412	0.2500000000000001	0.5292843097603157	T	T	T
0.5052870486247032	0.5000014561203573	0.4917168902426896	T	T	T
0.9962647480509502	0.7500000000000010	0.4556693226574557	T	T	T
0.6666666667000030	0.2500000000000000	0.3054125196999991	F	F	F
0.3333333333402032	0.2500000000000007	0.3815377613770973	T	T	T
0.1677425823242911	0.5000121293203219	0.1920345332174281	T	T	T
0.8333333333370314	0.9999993151585622	0.1922967608347688	T	T	T
0.4989240843485743	-0.0000121293209089	0.1920345332155970	T	T	T
0.1677425823242898	-0.0000121293203229	0.1920345332174280	T	T	T
0.6704214748082286	0.7499999999999996	0.1552233274240998	T	T	T
0.3333333333371091	0.7499999999999997	0.1518265624853549	T	T	T
0.9962451918673033	0.7499999999999993	0.1552233274251342	T	T	T
0.6704066195331950	0.2499999999999999	0.1551949479181706	T	T	T
0.3333333333370340	0.2500000000000008	0.1518172644229048	T	T	T
0.9962600471414255	0.2499999999999983	0.1551949479190270	T	T	T
0.4989240843485737	0.5000121293209087	0.1920345332155968	T	T	T
0.8333333333371286	0.5000078292892091	0.1154688361546258	T	T	T

0.1613806751033102 0.5000184760491037 0.1191588013628444 T T T
0.8333333333371259 -0.0000078292892119 0.1154688361546259 T T T
0.5052859915699819 -0.0000184760491507 0.1191588013620953 T T T
0.1613806751033096 -0.0000184760491058 0.1191588013628444 T T T
0.6567173340325451 0.7499999999999987 0.0816185108911350 T T T
0.0099493326426941 0.7500000000000018 0.0816185108916953 T T T
0.6566754146296894 0.2499999999999994 0.0815875709874126 T T T
0.0099912520454448 0.2499999999999950 0.0815875709879486 T T T
0.5052859915699850 0.5000184760491501 0.1191588013620961 T T T
0.8333333333370320 0.5000006848414364 0.1922967608347690 T T T
1.0003206979280788 0.2499999999999997 0.2298485269066751 T T T
0.3333333333412075 0.2499999999999993 0.2293062272461087 T T T
1.0003267591891489 0.2500000000000012 0.3810007542978572 T T T
0.8333333332999970 0.5000000000000000 0.3435890335000025 F F F
0.5000000000000000 0.5000000000000000 0.3435890335000025 F F F
0.1666666667000030 0.5000000000000000 0.3435890335000025 F F F
0.8333333332999970 0.0000000000000000 0.3435890335000025 F F F
0.5000000000000000 0.0000000000000000 0.3435890335000025 F F F
0.1666666667000030 0.0000000000000000 0.3435890335000025 F F F
0.6666666667000030 0.7500000000000000 0.3054125196999991 F F F
0.3333333332999970 0.7500000000000000 0.3054125196999991 F F F
0.0000000000000000 0.7500000000000000 0.3054125196999991 F F F
0.3333333332999970 0.2500000000000000 0.3054125196999991 F F F
0.0000000000000000 0.2500000000000000 0.3054125196999991 F F F
0.8333333332999970 0.5000000000000000 0.2672360059000027 F F F
0.5000000000000000 0.5000000000000000 0.2672360059000027 F F F
0.1666666667000030 0.5000000000000000 0.2672360059000027 F F F
0.8333333332999970 0.0000000000000000 0.2672360059000027 F F F
0.5000000000000000 0.0000000000000000 0.2672360059000027 F F F
0.1666666667000030 0.0000000000000000 0.2672360059000027 F F F
0.6663704105921965 0.7500000000000001 0.2299236064549254 T T T
0.3333333333422479 0.7499999999999996 0.2293126290533477 T T T
1.0002962560817270 0.7499999999999990 0.2299236064575615 T T T
0.6663459687351492 0.2499999999999994 0.2298485269038657 T T T
0.6663399074708257 0.2500000000000006 0.3810007543008617 T T T
0.6566811812971273 0.7499999999999999 0.5292816351135752 T T T

110_stoich/CONTCAR

Mg O
1.00857736950000
8.9333042307999992 0.0000000000000000 0.0000000000000000
0.0000000000000000 8.4223999999999997 0.0000000000000000
0.0000000000000000 0.0000000000000000 39.0000000000000000
Mg O
78 78
Selective dynamics
Direct
-0.0000023495665396 -0.0000052095438312 0.0841533752696232 T T T
0.4999995887984958 0.7500000000000003 0.4186987366522136 T T T
0.8333333333350023 0.7499999999999989 0.4186967130860433 T T T
0.9999986291206551 -0.0000024313881068 0.4552082484994159 T T T

0.8333333332999970	0.2500000000000000	0.2672360059000027	F	F	F
0.5000000000000000	0.2500000000000000	0.2672360059000027	F	F	F
0.1666666667000030	0.2500000000000000	0.2672360059000027	F	F	F
0.3333333333315970	-0.0000019321305465	0.4552081593463831	T	T	T
0.6666680375476806	-0.0000024313881270	0.4552082485003891	T	T	T
0.9999986291206564	0.5000024313881041	0.4552082484994159	T	T	T
0.6667387060396923	0.5001599805554601	0.2300476438774987	T	T	T
0.3333333333440197	0.4999927173843321	0.2300274320077182	T	T	T
-0.0000720393772463	0.5001599805478844	0.2300476438798152	T	T	T
0.3333333333315984	0.5000019321305443	0.4552081593463833	T	T	T
0.6666680375476821	0.5000024313881263	0.4552082485003889	T	T	T
0.3333333333423187	0.4999974621605203	0.3807960584262806	T	T	T
0.6667387060396900	-0.0001599805554590	0.2300476438774992	T	T	T
0.3333333333440183	1.0000072826156683	0.2300274320077181	T	T	T
-0.0000720393772476	-0.0001599805478820	0.2300476438798153	T	T	T
0.8333333333364624	0.7500000000000020	0.1922425012017810	T	T	T
0.1666666667000030	0.7500000000000000	0.2672360059000027	F	F	F
0.5000521962589372	0.7500000000000003	0.1921437092527920	T	T	T
0.5000000000000000	0.7500000000000000	0.2672360059000027	F	F	F
0.0000000000000000	0.0000000000000000	0.3054125196999991	F	F	F
0.6666572477207773	0.4999895516492268	0.3807993872966889	T	T	T
0.6666572477207786	1.0000104483507735	0.3807993872966887	T	T	T
0.3333333333423186	1.0000025378394783	0.3807960584262803	T	T	T
1.0000094189367779	1.0000104483511296	0.3807993872934219	T	T	T
0.8333333332999970	0.7500000000000000	0.3435890335000025	F	F	F
0.5000000000000000	0.7500000000000000	0.3435890335000025	F	F	F
0.1666666667000030	0.7500000000000000	0.3435890335000025	F	F	F
0.8333333332999970	0.2500000000000000	0.3435890335000025	F	F	F
0.5000000000000000	0.2500000000000000	0.3435890335000025	F	F	F
0.1666666667000030	0.2500000000000000	0.3435890335000025	F	F	F
0.1666675606719465	0.2500000000000009	0.4187007986683108	T	T	T
0.4999991059930636	0.2499999999999996	0.4187007986701076	T	T	T
0.8333333333348901	0.2499999999999968	0.4187014129631551	T	T	T
0.6666666667000030	0.5000000000000000	0.3054125196999991	F	F	F
0.3333333332999970	0.5000000000000000	0.3054125196999991	F	F	F
0.0000000000000000	0.5000000000000000	0.3054125196999991	F	F	F
0.1666670778663567	0.7499999999999996	0.4186987366503507	T	T	T
0.6666666667000030	0.0000000000000000	0.3054125196999991	F	F	F
0.3333333332999970	0.0000000000000000	0.3054125196999991	F	F	F
0.8333333333383024	0.7500000000000006	0.2677270170971028	T	T	T
0.1666144704128622	0.7500000000000011	0.1921437092540739	T	T	T
1.0000094189367810	0.4999895516488719	0.3807993872934220	T	T	T
0.0000001151690101	-0.0000000902735109	0.5266712551755695	T	T	T
0.3333333333336415	-0.0000128709315004	0.1556201639373573	T	T	T
-0.0000250700493509	-0.0000357235414280	0.1556246725923729	T	T	T
0.8333333333346753	0.7500000000000017	0.1161938866447000	T	T	T
0.4999999653291855	0.7500000000000016	0.1161784097156201	T	T	T
0.1666667013396023	0.7500000000000021	0.1161784097157979	T	T	T
0.4999991341314047	0.7500000000000000	0.4946508768619203	T	T	T
0.6666917367210626	-0.0000357235413556	0.1556246725916240	T	T	T
0.8333333333334272	0.7499999999999992	0.4946504422999604	T	T	T
0.6666690162352389	0.5000052095438456	0.0841533752695313	T	T	T

0.5000031933980595	0.2500000000000016	0.1161748475042322	T	T	T
0.1666634732706448	0.2500000000000016	0.1161748475044116	T	T	T
0.3333333333331834	1.0000000242907523	0.5266711414958881	T	T	T
0.0000001151690095	0.5000000902735118	0.5266712551755693	T	T	T
0.6666665514978560	-0.0000000902734721	0.5266712551757771	T	T	T
0.8333333333345386	0.2500000000000018	0.1161734023792432	T	T	T
0.3333333333342927	0.5000042923526887	0.0841527537331901	T	T	T
0.1666675325352563	0.7500000000000000	0.4946508768616166	T	T	T
0.3333333333342934	-0.0000042923526813	0.0841527537331902	T	T	T
0.3333333333336417	0.5000128709315038	0.1556201639373573	T	T	T
-0.0000023495665402	0.5000052095438342	0.0841533752696225	T	T	T
0.3333333333331814	0.4999999757092494	0.5266711414958878	T	T	T
0.6666665514978543	0.5000000902734715	0.5266712551757757	T	T	T
0.6666917367210626	0.5000357235413581	0.1556246725916233	T	T	T
0.8333333333354945	0.2500000000000006	0.1921360293590426	T	T	T
0.8333333333334682	0.2500000000000006	0.4946514992675450	T	T	T
0.4999998386136922	0.2499999999999993	0.4946512984999736	T	T	T
-0.0000250700493509	0.5000357235414297	0.1556246725923725	T	T	T
0.1666668280529001	0.2499999999999997	0.4946512984996931	T	T	T
0.5000491890672378	0.2500000000000012	0.1921323767507489	T	T	T
0.6666690162352372	-0.0000052095438410	0.0841533752695317	T	T	T
0.1666174776025912	0.2500000000000023	0.1921323767520338	T	T	T
0.3333333333402709	0.7500000000000000	0.3809418764953709	T	T	T
0.6666713419182900	0.7500000000000010	0.3809313809017643	T	T	T
0.1666652603634473	-0.0000022860108439	0.4185338703224029	T	T	T
0.9999998454491832	0.7500000000000004	0.5286839357321842	T	T	T
0.6666666482604942	0.2500000000000007	0.5286849433202006	T	T	T
-0.0000046752587487	0.7500000000000007	0.3809313808983792	T	T	T
0.5000014063029100	-0.0000022860107945	0.4185338703242023	T	T	T
0.6666675913550106	0.7500000000000001	0.4560489814749529	T	T	T
0.3333333333330483	0.2500000000000015	0.5286850177772111	T	T	T
0.1666662386181508	-0.0000004365981478	0.4934278087036911	T	T	T
0.5000004280482730	-0.0000004365981386	0.4934278087039860	T	T	T
0.8333333333336139	-0.0000006755079635	0.4934276586366201	T	T	T
0.1666662386181491	0.5000004365981487	0.4934278087036913	T	T	T
0.6666676221965019	0.2499999999999988	0.4560513951131458	T	T	T
0.3333333333322174	0.2499999999999973	0.4560514846003339	T	T	T
0.8333333333335249	-0.0000049872691365	0.4185320242165166	T	T	T
-0.0000009555286049	0.2500000000000006	0.4560513951125535	T	T	T
0.8333333333336118	0.5000006755079641	0.4934276586366205	T	T	T
0.3333333333321950	0.7500000000000009	0.4560510740289265	T	T	T
0.8333333333335260	0.5000049872691363	0.4185320242165164	T	T	T
0.5000014063029106	0.5000022860107936	0.4185338703242022	T	T	T
0.1666652603634478	0.5000022860108427	0.4185338703224026	T	T	T
0.0000000184064899	0.2500000000000003	0.5286849433199911	T	T	T
0.5000004280482737	0.5000004365981403	0.4934278087039863	T	T	T
0.9999990753128744	0.7500000000000003	0.4560489814742916	T	T	T
0.6666666667000030	0.2500000000000000	0.3054125196999991	F	F	F
0.3333333333403842	0.2499999999999985	0.3809440555117705	T	T	T
0.1666512764120586	0.5000218424137669	0.1922947120278191	T	T	T
0.8333333333360614	-0.0000156340684198	0.1923004547222511	T	T	T
0.5000153902584570	-0.0000218424143399	0.1922947120261968	T	T	T

0.1666512764120575	-0.0000218424137659	0.1922947120278196	T	T	T
0.6666887866677468	0.7500000000000030	0.1547956461619286	T	T	T
0.3333333333342727	0.7500000000000021	0.1547763226483835	T	T	T
-0.0000221199962605	0.7500000000000034	0.1547956461626134	T	T	T
0.6666758136251976	0.2499999999999999	0.1547723589817176	T	T	T
0.3333333333342340	0.2500000000000031	0.1547763421919000	T	T	T
-0.0000091469542626	0.2500000000000028	0.1547723589822463	T	T	T
0.5000153902584576	0.5000218424143404	0.1922947120261963	T	T	T
0.8333333333347465	0.5000010715275131	0.1173964971261046	T	T	T
0.1666643555680135	0.5000050795225446	0.1173961298212572	T	T	T
0.8333333333347431	-0.0000010715275117	0.1173964971261052	T	T	T
0.5000023111006707	-0.0000050795225380	0.1173961298210659	T	T	T
0.1666643555680095	-0.0000050795225399	0.1173961298212570	T	T	T
0.6666767232186107	0.7500000000000016	0.0821461411056569	T	T	T
0.3333333333341665	0.7500000000000016	0.0821372018393052	T	T	T
-0.0000100565497287	0.7500000000000002	0.0821461411058025	T	T	T
0.6666647587958162	0.2500000000000022	0.0821342198077234	T	T	T
0.3333333333340788	0.2500000000000018	0.0821366838518308	T	T	T
1.0000019078729538	0.2500000000000020	0.0821342198078539	T	T	T
0.5000023111006704	0.5000050795225426	0.1173961298210663	T	T	T
0.8333333333360604	0.5000156340684199	0.1923004547222509	T	T	T
-0.0000131674539022	0.2500000000000007	0.2298874888167756	T	T	T
0.3333333333410761	0.2499999999999994	0.2298805680042156	T	T	T
-0.0000051461448248	0.2500000000000001	0.3809426588286700	T	T	T
0.8333333332999970	0.5000000000000000	0.3435890335000025	F	F	F
0.5000000000000000	0.5000000000000000	0.3435890335000025	F	F	F
0.1666666667000030	0.5000000000000000	0.3435890335000025	F	F	F
0.8333333332999970	0.0000000000000000	0.3435890335000025	F	F	F
0.5000000000000000	0.0000000000000000	0.3435890335000025	F	F	F
0.1666666667000030	0.0000000000000000	0.3435890335000025	F	F	F
0.6666666667000030	0.7500000000000000	0.3054125196999991	F	F	F
0.3333333332999970	0.7500000000000000	0.3054125196999991	F	F	F
0.0000000000000000	0.7500000000000000	0.3054125196999991	F	F	F
0.3333333333330565	0.7500000000000014	0.5286849703520039	T	T	T
0.3333333332999970	0.2500000000000000	0.3054125196999991	F	F	F
0.0000000000000000	0.2500000000000000	0.3054125196999991	F	F	F
0.8333333332999970	0.5000000000000000	0.2672360059000027	F	F	F
0.5000000000000000	0.5000000000000000	0.2672360059000027	F	F	F
0.1666666667000030	0.5000000000000000	0.2672360059000027	F	F	F
0.8333333332999970	0.0000000000000000	0.2672360059000027	F	F	F
0.5000000000000000	0.0000000000000000	0.2672360059000027	F	F	F
0.1666666667000030	0.0000000000000000	0.2672360059000027	F	F	F
0.6667118722905764	0.7500000000000002	0.2299661158803470	T	T	T
0.3333333333421424	0.7500000000000011	0.2298851591290031	T	T	T
-0.0000452056172615	0.7500000000000008	0.2299661158829089	T	T	T
0.6666798341161433	0.2500000000000006	0.2298874888139988	T	T	T
0.6666718128042481	0.2499999999999986	0.3809426588316470	T	T	T
0.6666668212178372	0.7500000000000012	0.5286839357324385	T	T	T

111_2x2_octo/CONTCAR

```
Mg O
   1.00857736950000
     5.9555361538999998    0.0000000000000000    0.0000000000000000
    -2.9777680768999999    5.1576456023999997    0.0000000000000000
     0.0000000000000000    0.0000000000000000   39.1760745361000033
   Mg   O
    34    34
Selective dynamics
Direct
  0.8333333332999970  0.6666666667000030  0.3723698057999982   F   F   F
  0.9986379368212669  0.4993189684456892  0.3115054746612503   T   T   T
  0.5006810315543234  0.4993189684461652  0.3115054746611507   T   T   T
  0.3333333332999970  0.1666666667000030  0.3723698057999982   F   F   F
  0.8333333332999970  0.1666666667000030  0.3723698057999982   F   F   F
  0.3333333332999970  0.6666666667000030  0.3723698057999982   F   F   F
  0.1666666667000030  0.3333333332999970  0.4344315995000017   F   F   F
  0.6666666667000030  0.3333333332999970  0.4344315995000017   F   F   F
  0.1666666667000030  0.8333333332999970  0.4344315995000017   F   F   F
  0.6666666667000030  0.8333333332999970  0.4344315995000017   F   F   F
  0.0000000000233174 -0.0000000000236381  0.4954932617721392   T   T   T
  0.5006810315546650  0.0013620631789535  0.3115054746612409   T   T   T
  0.4993189684458810  0.9986379368221323  0.4952959306273900   T   T   T
  0.4993189684462157  0.5006810315532907  0.4952959306274810   T   T   T
  0.3369496581299979  0.1684748290394798  0.5559936944740552   T   T   T
  0.8315251709587961  0.1684748290405171  0.5559936944737430   T   T   T
  0.3333333333493717  0.6666666666499241  0.5569183726258775   T   T   T
  0.8315251709595448  0.6630503418687496  0.5559936944740232   T   T   T
  0.1571561300651340  0.3143122601031869  0.6152511153082706   T   T   T
  0.6666666666721054  0.3333333333226895  0.6222349895672199   T   T   T
  0.1571561300660698  0.8428438699302877  0.6152511153087507   T   T   T
  0.6856877398961959  0.8428438699327268  0.6152511153082910   T   T   T
  0.0000000000062494 -0.0000000000082202  0.6670401331513334   T   T   T
  0.0013620631776442  0.5006810315537649  0.4952959306273804   T   T   T
 -0.0000000000233147  0.0000000000236425  0.3113081435172049   T   T   T
  0.8428438699350070  0.1571561300686527  0.1915502899661111   T   T   T
  0.3143122601016622  0.1571561300661998  0.1915502899665713   T   T   T
  0.3333333333278908  0.6666666666773110  0.1845664157106926   T   T   T
  0.8428438699359357  0.6856877398989509  0.1915502899665911   T   T   T
 -0.0000000000062471  0.0000000000082211  0.1397612721228442   T   T   T
  0.1684748290374615  0.3369496581252677  0.2508077108057332   T   T   T
  0.6666666666506226  0.3333333333500697  0.2498830326523525   T   T   T
  0.6630503418759813  0.8315251709635063  0.2508077108057006   T   T   T
  0.1684748290382167  0.8315251709624742  0.2508077108060134   T   T   T
  0.3161484216422377  0.1580742108076542  0.6435248374598213   T   T   T
  0.6650288185351564  0.8325144092375583  0.5258240443977296   T   T   T
  0.1580742108119432  0.8419257891882511  0.1632765678120052   T   T   T
  0.1580742108114114  0.3161484216495448  0.1632765678116506   T   T   T
  0.8419257891903330  0.6838515783539419  0.6435248374597194   T   T   T
  0.4976375405522291  0.9952750810691083  0.5879497944715305   T   T   T
  0.0047249189299025  0.5023624594462199  0.5879497944712559   T   T   T
```

```
 0.4976375405546766  0.5023624594431995  0.5879497944715578   T   T   T
 0.1674855907616759  0.8325144092378941  0.5258240443976611   T   T   T
 0.8419257891897911  0.1580742108100092  0.6435248374593662   T   T   T
 0.0000000000119823 -0.0000000000135817  0.5839160470004181   T   T   T
 0.6666666666857164  0.3333333333131842  0.5261879873386793   T   T   T
 0.3333333333142860  0.6666666666868183  0.2806134179287130   T   T   T
 0.6838515783542731  0.8419257891906026  0.1632765678115497   T   T   T
 0.8325144092375427  0.1674855907628898  0.2809773608699019   T   T   T
 0.3349711814664170  0.1674855907632323  0.2809773608698339   T   T   T
 0.1667745540294813  0.3335491081361945  0.3418565469446903   T   T   T
 0.6666666666408873  0.3333333333592249  0.3420955281166855   T   T   T
 0.1667745540293742  0.8332254459708210  0.3418565469446945   T   T   T
 0.6664508918639687  0.8332254459706941  0.3418565469447133   T   T   T
 0.5023624594456315  0.4976375405564857  0.2188516107987422   T   T   T
 0.0000000000000000  0.0000000000000000  0.4034007026000026   F   F   F
 0.5000000000000000  0.0000000000000000  0.4034007026000026   F   F   F
 0.0000000000000000  0.5000000000000000  0.4034007026000026   F   F   F
 0.5000000000000000  0.5000000000000000  0.4034007026000026   F   F   F
 0.9952750810694708  0.4976375405534670  0.2188516107990438   T   T   T
 0.5023624594480806  0.0047249189315152  0.2188516107987685   T   T   T
-0.0000000000119824  0.0000000000135817  0.2228853582713501   T   T   T
 0.3335491081363010  0.1667745540294392  0.4649448583202441   T   T   T
 0.8332254459704951  0.1667745540293161  0.4649448583202620   T   T   T
 0.3333333333591177  0.6666666666407802  0.4647058771488777   T   T   T
 0.8332254459703876  0.6664508918635388  0.4649448583202662   T   T   T
 0.8325144092373250  0.6650288185342701  0.2809773608698811   T   T   T
 0.1674855907618861  0.3349711814641574  0.5258240443976824   T   T   T
```

111_stoich/CONTCAR

```
Mg O
  1.00857736950000
    5.9555361538999998    0.0000000000000000    0.0000000000000000
   -2.9777680768999999    5.1576456023999997    0.0000000000000000
    0.0000000000000000    0.0000000000000000   39.1760745361000033
  Mg   O
   40   40
Selective dynamics
Direct
 0.8333333333193380  0.6666666666666643  0.3723698058251728   F   F   F
 0.9999999999916014  0.5000000000000000  0.3103080121822259   F   F   F
 0.5000000000000000  0.5000000000000000  0.3103080121822259   F   F   F
 0.3333333333361352  0.1666666666666643  0.3723698058251728   F   F   F
 0.8333333333277366  0.1666666666666643  0.3723698058251728   F   F   F
 0.3333333333277366  0.6666666666666643  0.3723698058251728   F   F   F
 0.9999999999929392  0.4999999999990362  0.6716963878144225   T   T   T
 0.1666666666638648  0.3333333333333357  0.4344315994706704   F   F   F
 0.6666666666554733  0.3333333333333357  0.4344315994706704   F   F   F
 0.1666666666554733  0.8333333333333357  0.4344315994706704   F   F   F
 0.6666666666638719  0.8333333333333357  0.4344315994706704   F   F   F
-0.0000000000053346  0.0000000000006084  0.4952859594771548   T   T   T
 0.4999999999916014  0.0000000000000000  0.3103080121822259   F   F   F
```

0.4999999999952650	-0.0000000000009191	0.4952859594770529	T	T	T
0.4999999999930869	0.4999999999998183	0.4952859594772911	T	T	T
0.3333333333269700	0.1666666666660677	0.5557914436681882	T	T	T
0.8333333333267701	0.1666666666663931	0.5557914436681981	T	T	T
0.3333333333264862	0.6666666666662501	0.5557914436682028	T	T	T
0.8333333333267497	0.6666666666662103	0.5557914436681847	T	T	T
0.1666666666600024	0.3333333333326382	0.6147711184529615	T	T	T
0.6666666666600978	0.3333333333329327	0.6147711184529233	T	T	T
0.1666666666597766	0.8333333333327740	0.6147711184529584	T	T	T
0.6666666666601910	0.8333333333328008	0.6147711184529270	T	T	T
-0.0000000000071096	-0.0000000000009323	0.6716963878145539	T	T	T
0.4999999999929092	-0.0000000000009373	0.6716963878142794	T	T	T
0.9999999999927531	0.4999999999998651	0.4952859594769157	T	T	T
0.0000000000000000	0.0000000000000000	0.3103080121822259	F	F	F
0.4999999999929545	0.4999999999990991	0.6716963878144109	T	T	T
0.8333333333318432	0.1666666666685313	0.1860767660746104	T	T	T
0.3333333333315984	0.1666666666681268	0.1860767660746416	T	T	T
0.3333333333307892	0.6666666666680032	0.1860767660746130	T	T	T
0.5000000000007091	0.5000000000029247	0.1229167783622403	T	T	T
0.8333333333305211	0.6666666666686477	0.1860767660746397	T	T	T
0.5000000000021777	0.0000000000031371	0.1229167783622519	T	T	T
0.0000000000021612	0.0000000000036419	0.1229167783622384	T	T	T
0.1666666666629366	0.3333333333343189	0.2486047153509079	T	T	T
0.6666666666631589	0.3333333333337548	0.2486047153506463	T	T	T
1.0000000000007336	0.5000000000038612	0.1229167783622483	T	T	T
0.6666666666626719	0.8333333333344346	0.2486047153508926	T	T	T
0.1666666666629273	0.8333333333336389	0.2486047153506599	T	T	T
0.3333333333249218	0.1666666666650894	0.6485690133400568	T	T	T
0.8333333333338118	0.1666666666695025	0.1012073888193989	T	T	T
0.6666666666596315	0.8333333333328800	0.5267874799118744	T	T	T
0.3333333333323696	0.6666666666687843	0.1012073888193987	T	T	T
0.1666666666625064	0.8333333333339497	0.1603722108086415	T	T	T
0.6666666666637550	0.3333333333345722	0.1603722108086389	T	T	T
0.1666666666639347	0.3333333333339245	0.1603722108086279	T	T	T
0.8333333333251620	0.6666666666651697	0.6485690133400572	T	T	T
0.4999999999919483	-0.0000000000012510	0.5892526612588380	T	T	T
0.9999999999923163	0.4999999999988462	0.5892526612588070	T	T	T
0.4999999999920582	0.4999999999987491	0.5892526612588184	T	T	T
0.1666666666592297	0.8333333333324615	0.5267874799116471	T	T	T
0.3333333333250330	0.6666666666649926	0.6485690133400401	T	T	T
0.8333333333251827	0.1666666666650684	0.6485690133400411	T	T	T
0.8333333333322581	0.6666666666693645	0.1012073888193265	T	T	T
-0.0000000000077446	-0.0000000000011546	0.5892526612587874	T	T	T
0.6666666666600827	0.3333333333328857	0.5267874799118176	T	T	T
0.3333333333284018	0.6666666666668529	0.2805046831908102	T	T	T
0.6666666666627061	0.8333333333345411	0.1603722108086244	T	T	T
0.8333333333279640	0.1666666666666348	0.2805046831908610	T	T	T
0.3333333333294013	0.1666666666668598	0.2805046831905726	T	T	T
0.1666666666638648	0.3333333333333357	0.3413389090062537	F	F	F
0.6666666666554733	0.3333333333333357	0.3413389090062537	F	F	F
0.1666666666554733	0.8333333333333357	0.3413389090062537	F	F	F
0.6666666666638719	0.8333333333333357	0.3413389090062537	F	F	F

0.4999999999958850	0.5000000000006590	0.2199126633374705	T	T	T
0.0000000000000000	0.0000000000000000	0.4034007026491935	F	F	F
0.4999999999916014	0.0000000000000000	0.4034007026491935	F	F	F
0.9999999999916014	0.5000000000000000	0.4034007026491935	F	F	F
0.5000000000000000	0.5000000000000000	0.4034007026491935	F	F	F
0.9999999999959337	0.5000000000002601	0.2199126633374001	T	T	T
0.4999999999956372	0.0000000000004223	0.2199126633373865	T	T	T
-0.0000000000044368	0.0000000000005079	0.2199126633374831	T	T	T
0.3333333333361352	0.1666666666666643	0.4654624962921403	F	F	F
0.8333333333277366	0.1666666666666643	0.4654624962921403	F	F	F
0.3333333333277366	0.6666666666666643	0.4654624962921403	F	F	F
0.8333333333193380	0.6666666666666643	0.4654624962921403	F	F	F
0.8333333333290438	0.6666666666666425	0.2805046831906224	T	T	T
0.1666666666597565	0.3333333333324440	0.5267874799117045	T	T	T
0.3333333333337126	0.1666666666686517	0.1012073888193273	T	T	T

13. Coordinates of Fe_3O_4(111) Surfaces – MLFF optimized

```
CONTCAR_OCT1.vasp
   1.00000000000000
     6.0163998604000000    0.0000000000000000    0.0000000000000000
    -3.0081999302000000    5.2103551183999999    0.0000000000000000
     0.0000000000000000    0.0000000000000000   44.5619010925000012
   Fe   O
    27    32
Selective dynamics
Direct
  0.6666660630342550  0.3333339375923917  0.3263980238805114   T   T   T
  0.3333300053932007  0.6666700244160495  0.4310990730427591   F   F   F
  0.0000000000000000  0.0000000000000000  0.5413446903426689   F   F   F
  0.6666682156384278  0.3333318012302809  0.6423551159963450   T   T   T
  0.3333318002727711  0.6666682165614133  0.3576459704459391   T   T   T
  0.0000000000000000  0.0000000000000000  0.4586604819119771   F   F   F
  0.6666700244644730  0.3333300054475643  0.5688938202923026   F   F   F
  0.3333339386452053  0.6666660619715227  0.6736017829796225   T   T   T
 -0.0000010248545691  0.0000010338128279  0.3429612721040476   T   T   T
  0.6666700244644730  0.3333300054475643  0.4448858987153201   F   F   F
  0.3333300053932007  0.6666700244160495  0.5551192553174857   F   F   F
  0.0000010342450670 -0.0000010252896263  0.6570397175170124   T   T   T
  0.1697659413838162  0.3395322773363901  0.2942515451143734   T   T   T
  0.8324903302388874  0.6649864242503329  0.3925373001833161   T   T   T
  0.4999999999667608  0.0000000000000000  0.4999964557784509   F   F   F
  0.1675116699571912  0.3350175664030182  0.6074640692064323   T   T   T
  0.8302351156131917  0.6604698535816013  0.7057484399707462   T   T   T
  0.1697664237489432  0.8302335490677071  0.2942515623674004   T   T   T
  0.8324922209789468  0.1675077990661919  0.3925370520471826   T   T   T
  0.4999999999641886  0.4999999999616165  0.4999964557784509   F   F   F
  0.1675097822606625  0.8324902378351990  0.6074643172858402   T   T   T
  0.8302346055944380  0.1697653670644260  0.7057484220871275   T   T   T
  0.6604677327378602  0.8302340480504585  0.2942515453960717   T   T   T
  0.3350135956104939  0.1675096858772422  0.3925373002301415   T   T   T
  0.9999999999974278  0.4999999999616165  0.4999964557784509   F   F   F
  0.6649824534783552  0.8324883461963886  0.6074640692504579   T   T   T
  0.3395301565189280  0.1697648737637628  0.7057484402532470   T   T   T
  0.6666656737025014  0.3333343452843711  0.3686495572442117   T   T   T
  0.3333300053932007  0.6666700244160495  0.4739043418539026   F   F   F
  0.0000011884354916 -0.0000011716484031  0.5851351788199831   T   T   T
  0.6666664294067555  0.3333335618287490  0.6874358133686453   T   T   T
  0.8352055658358297  0.6704119755343317  0.3089654996828057   T   T   T
  0.5134674176754075  0.0269370978437026  0.4174289098867145   T   T   T
  0.1725499928098628  0.3451099992493738  0.5270680935996452   F   F   F
  0.8463219045709502  0.6926420321594546  0.6333148176517152   T   T   T
  0.8352082072040035  0.1647918101349582  0.3089661007764948   T   T   T
  0.5134675712952896  0.4865324448284253  0.4174294128486745   T   T   T
  0.1725499927475340  0.8274499774065163  0.5270680935996452   F   F   F
  0.8463234598801740  0.1536765566755201  0.6333147984648637   T   T   T
  0.3295880430865833  0.1647944498429577  0.3089655015464349   T   T   T
  0.9730629288942415  0.4865326081225831  0.4174289106889478   T   T   T
```

```
  0.6548900007813501  0.8274499774065163  0.5270680935996452   F   F   F
  0.3073579849894187  0.1536781144943916  0.6333148178296737   T   T   T
  0.1536772792135461  0.3073563178202226  0.3666856650976539   T   T   T
  0.8274499774624573  0.6548900008657839  0.4729248179572636   F   F   F
  0.4865338232411922  0.9730653618990306  0.5825716863649779   T   T   T
  0.1647929714107932  0.3295850876006617  0.6910338543050357   T   T   T
  0.3333335619475128  0.6666664293450254  0.3125645954679876   T   T   T
 -0.0000011730635332  0.0000011898127659  0.4148658111478438   T   T   T
  0.6666700244644730  0.3333300054475643  0.5261008121564572   F   F   F
  0.3333343443317068  0.6666656746748516  0.6313512615745346   T   T   T
  0.1536757220432715  0.8463242944807956  0.3666856847753142   T   T   T
  0.8274499773475057  0.1725499927682606  0.4729248179572636   F   F   F
  0.4865336576772828  0.5134663584634477  0.5825711839686822   T   T   T
  0.1647903228850427  0.8352096945278960  0.6910332551921854   T   T   T
  0.6926436992768801  0.8463227398232256  0.3666856652737954   T   T   T
  0.3451099992319087  0.1725499927682606  0.4729248179572636   F   F   F
  0.0269346648582641  0.5134662025784134  0.5825716871701205   T   T   T
  0.6704149310831096  0.8352070443453182  0.6910338561804521   T   T   T


CONTCAR_OCT2.vasp
   1.00000000000000
     6.0163998604000000    0.0000000000000000    0.0000000000000000
    -3.0081999302000000    5.2103551183999999    0.0000000000000000
     0.0000000000000000    0.0000000000000000   44.5619010925000012
   Fe   O
    31   40
Selective dynamics
Direct
  0.6666663535460077  0.3333336558907586  0.3259710248392567   T   T   T
  0.3333300053932007  0.6666700244160495  0.4310990730427591   F   F   F
  0.0000000000000000  0.0000000000000000  0.5413446903426689   F   F   F
  0.6666679424430842  0.3333320770743499  0.6473482917242332   T   T   T
  0.3333332071884592  0.6666667931668459  0.7531650476647669   T   T   T
  0.6666667931703364  0.3333332071914272  0.2468351103856691   T   T   T
  0.3333320752604368  0.6666679442090407  0.3526515395955447   T   T   T
  0.0000000000000000  0.0000000000000000  0.4586604819119771   F   F   F
  0.6666700244644730  0.3333300054475643  0.5688938202923026   F   F   F
  0.3333336553475643  0.6666663540947236  0.6740294421907205   T   T   T
  0.3333339585600896  0.6666660383636406  0.2394620569191799   T   T   T
 -0.0000004477880187  0.0000004634977455  0.3400044093194102   T   T   T
  0.6666700244644730  0.3333300054475643  0.4448858987153201   F   F   F
  0.3333300053932007  0.6666700244160495  0.5551192553174857   F   F   F
  0.0000004619682850 -0.0000004462421515  0.6599953722364781   T   T   T
  0.6666660383287855  0.3333339585675456  0.7605379655241844   T   T   T
  0.1611173952490358  0.3222358589794014  0.2878742343545879   T   T   T
  0.8331446760307292  0.6662951804271252  0.3921072728136933   T   T   T
  0.4999999999667608  0.0000000000000000  0.4999964557784509   F   F   F
  0.1668571910781764  0.3337085336657792  0.6078937349677018   T   T   T
  0.8388823892401100  0.6777637021680095  0.7121254949039746   T   T   T
  0.1611170509505526  0.8388829511964404  0.2878741929568121   T   T   T
  0.8331466544207350  0.1668533712106826  0.3921070059117369   T   T   T
```

0.4999999999641886	0.4999999999616165	0.4999964557784509	F	F	F
0.1668552123680156	0.8331448133280099	0.6078940018442517	T	T	T
0.8388827322300402	0.1611172699191540	0.7121255370270504	T	T	T
0.6777641435230900	0.8388826093135204	0.2878742347344884	T	T	T
0.3337048447639156	0.1668553495852305	0.3921072727750967	T	T	T
0.9999999999974278	0.4999999999616165	0.4999964557784509	F	F	F
0.6662914915508487	0.8331428345851519	0.6078937349262593	T	T	T
0.3222363003398646	0.1611176153295674	0.7121254952836582	T	T	T
-0.0000001956635374	0.0000001985699414	0.2674665830724335	T	T	T
0.6666659501787837	0.3333340749763675	0.3691682741293918	T	T	T
0.3333300053932007	0.6666700244160495	0.4739043418539026	F	F	F
0.0000009439445843	-0.0000009211093378	0.5852396584913232	T	T	T
0.6666672555345944	0.3333327503392230	0.6908194663515717	T	T	T
0.8428402317565046	0.6856815815928895	0.3119283046893438	T	T	T
0.5137636151780024	0.0275287649442245	0.4175263112556578	T	T	T
0.1725499928098628	0.3451099992493738	0.5270680935996452	F	F	F
0.8463059787690159	0.6926100095230860	0.6332587633089917	T	T	T
0.5082733141970737	0.0165469195960790	0.7413405550276497	T	T	T
0.8428404280215416	0.1571595811891739	0.3119282999132197	T	T	T
0.5137633301217974	0.4862366933722120	0.4175267551825253	T	T	T
0.1725499927475340	0.8274499774065163	0.5270680935996452	F	F	F
0.8463069569996585	0.1536930656787204	0.6332585892936726	T	T	T
0.5082728127130268	0.4917271864845548	0.7413403315098150	T	T	T
0.3143184260436989	0.1571597744663157	0.3119283045264091	T	T	T
0.9724712652106079	0.4862364129065793	0.4175263120852336	T	T	T
0.6548900007813501	0.8274499774065163	0.5270680935996452	F	F	F
0.3073900130882701	0.1536940445006087	0.6332587634411806	T	T	T
0.9834530775490014	0.4917266843679305	0.7413405551717898	T	T	T
0.4917275694582178	0.9834548473567570	0.2586592938620857	T	T	T
0.1536929461883499	0.3073878219661505	0.3667412951451745	T	T	T
0.8274499774624573	0.6548900008657839	0.4729248179572636	F	F	F
0.4862374690155514	0.9724733824824939	0.5824739198287380	T	T	T
0.1571601951650575	0.3143192691247326	0.6880715721856396	T	T	T
0.3333327491136940	0.6666672567368176	0.3091803305100386	T	T	T
-0.0000009228240637	0.0000009456223882	0.4147617373050453	T	T	T
0.6666700244644730	0.3333300054475643	0.5261008121564572	F	F	F
0.3333340731957414	0.6666659519883128	0.6308320650511655	T	T	T
0.0000001970154449	-0.0000001940868841	0.7325330000137378	T	T	T
0.4917280714761665	0.5082719277290438	0.2586595175350596	T	T	T
0.1536919677396081	0.8463080549103595	0.3667414693614031	T	T	T
0.8274499773475057	0.1725499927682606	0.4729248179572636	F	F	F
0.4862377475615501	0.5137622759499122	0.5824734764086312	T	T	T
0.1571600016591743	0.8428400075690224	0.6880715766263704	T	T	T
0.0165451498076785	0.5082724291269980	0.2586592940040471	T	T	T
0.6926122005959952	0.8463070770450568	0.3667412952754197	T	T	T
0.3451099992319087	0.1725499927682606	0.4729248179572636	F	F	F
0.0275266476948097	0.5137625590943252	0.5824739206603677	T	T	T
0.6856807385109809	0.8428398110607520	0.6880715720225924	T	T	T

```
CONTCAR_OXY1.vasp
   1.00000000000000
     6.0163998604000000    0.0000000000000000    0.0000000000000000
    -3.0081999302000000    5.2103551183999999    0.0000000000000000
     0.0000000000000000    0.0000000000000000   44.5619010925000012
   Fe   O
    27    40
Selective dynamics
Direct
  0.6666666032693591  0.3333334029074975  0.3235901134885674   T   T   T
  0.3333300053932007  0.6666700244160495  0.4310990730427591   F   F   F
  0.0000000000000000  0.0000000000000000  0.5413446903426689   F   F   F
  0.6666675492248997  0.3333324644708424  0.6455666902731418   T   T   T
  0.3333324630000535  0.6666675506645492  0.3544330429900720   T   T   T
  0.0000000000000000  0.0000000000000000  0.4586604819119771   F   F   F
  0.6666700244644730  0.3333300054475643  0.5688938202923026   F   F   F
  0.3333334020676479  0.6666666041123590  0.6764101311018723   T   T   T
 -0.0000000675983119  0.0000000773799095  0.3392303796255666   T   T   T
  0.6666700244644730  0.3333300054475643  0.4448858987153201   F   F   F
  0.3333300053932007  0.6666700244160495  0.5551192553174857   F   F   F
  0.0000000759837161 -0.0000000662020211  0.6607696620717985   T   T   T
  0.1713234597715578  0.3426472096215599  0.2856453548072545   T   T   T
  0.8324482341049451  0.6649015020039872  0.3919701617355643   T   T   T
  0.4999999999667608  0.0000000000000000  0.4999964557784509   F   F   F
  0.1675526901940571  0.3351003322483126  0.6080307321940021   T   T   T
  0.8286787362928913  0.6573571818644052  0.7143545713381130   T   T   T
  0.1713233410778965  0.8286766559150526  0.2856453671972368   T   T   T
  0.8324500102724932  0.1675500114162026  0.3919699096987419   T   T   T
  0.4999999999641886  0.4999999999616165  0.4999964557784509   F   F   F
  0.1675509152170120  0.8324491065296323  0.6080309841125244   T   T   T
  0.8286788521211488  0.1713211448594759  0.7143545588576073   T   T   T
  0.6573527902707240  0.8286765419418216  0.2856453548142664   T   T   T
  0.3350985193225747  0.1675517867771775  0.3919701616017711   T   T   T
  0.9999999999974278  0.4999999999616165  0.4999964557784509   F   F   F
  0.6648996891000430  0.8324473307274910  0.6080307320577345   T   T   T
  0.3426428180356382  0.1713212654023833  0.7143545713460686   T   T   T
 -0.0000001310123468  0.0000001299269445  0.2684692811849641   T   T   T
  0.6666660459982756  0.3333339746152177  0.3684072995586287   T   T   T
  0.3333300053932007  0.6666700244160495  0.4739043418539026   F   F   F
  0.0000008988955163 -0.0000008782861383  0.5850256056294029   T   T   T
  0.6666669519759250  0.3333330511932096  0.6872189853355326   T   T   T
  0.8469837853867569  0.6939678794149988  0.3124913608321974   T   T   T
  0.5143484804957685  0.0286985260015234  0.4175953740603742   T   T   T
  0.1725499928098628  0.3451099992493738  0.5270680935996452   F   F   F
  0.8472974296287710  0.6945932838987615  0.6325446488217765   T   T   T
  0.5178091086562248  0.0356176361775451  0.7321199648376512   T   T   T
  0.8469838276693448  0.1530161710943763  0.3124913578598911   T   T   T
  0.5143484473298658  0.4856515744647059  0.4175958307557459   T   T   T
  0.1725499927475340  0.8274499774065163  0.5270680935996452   F   F   F
  0.8472981196810528  0.1527018978819913  0.6325445109649162   T   T   T
  0.5178092272065773  0.4821907749028569  0.7321199583123799   T   T   T
  0.3060321250827890  0.1530162219097587  0.3124913607092301   T   T   T
```

```
 0.9713015033165331  0.4856515464730504  0.4175953746947616   T   T   T
 0.6548900007813501  0.8274499774065163  0.5270680935996452   F   F   F
 0.3054067320327061  0.1527025866805218  0.6325446490109445   T   T   T
 0.9643823627725574  0.4821908880879912  0.7321199650876735   T   T   T
 0.4821909436318944  0.9643824735674361  0.2678799862130582   T   T   T
 0.1527016984723600  0.3054049612003875  0.3674553939161556   T   T   T
 0.8274499774624573  0.6548900008657839  0.4729248179572636   F   F   F
 0.4856524061117162  0.9713032273221642  0.5824047883254889   T   T   T
 0.1530163219229127  0.3060323270513552  0.6875086547284666   T   T   T
 0.3333330506916694  0.6666669524708482  0.3127814573934165   T   T   T
-0.0000008797114557  0.0000009002887388  0.4149751395169773   T   T   T
 0.6666700244644730  0.3333300054475643  0.5261008121564572   F   F   F
 0.3333339729697796  0.6666660476668187  0.6315929560542304   T   T   T
 0.0000001296561838 -0.0000001307427841  0.7315304803134933   T   T   T
 0.4821908313903447  0.5178091707249689  0.2678799929646258   T   T   T
 0.1527010101809184  0.8472990073608269  0.3674555320517556   T   T   T
 0.8274499773475057  0.1725499927682606  0.4729248179572636   F   F   F
 0.4856524320306608  0.5143475897860618  0.5824043318915140   T   T   T
 0.1530162707705551  0.8469837280002018  0.6875086575153034   T   T   T
 0.0356175253702187  0.5178090530950620  0.2678799864617074   T   T   T
 0.6945950546958695  0.8472983178080901  0.3674553941036095   T   T   T
 0.3451099992319087  0.1725499927682606  0.4729248179572636   F   F   F
 0.0286968020165952  0.5143476208777746  0.5824047889622229   T   T   T
 0.6939676774444670  0.8469836853650961  0.6875086546054477   T   T   T

CONTCAR_OXY2.vasp
   1.00000000000000
     6.0163998604000000    0.0000000000000000    0.0000000000000000
    -3.0081999302000000    5.2103551183999999    0.0000000000000000
     0.0000000000000000    0.0000000000000000   44.5619010925000012
   Fe   O
    21    32
Selective dynamics
Direct
 0.6666744119362762  0.3333256299956150  0.3254953444216549   T   T   T
 0.3333300053932007  0.6666700244160495  0.4310990730427591   F   F   F
 0.0000000000000000  0.0000000000000000  0.5413446903426689   F   F   F
 0.6666698499554136  0.3333301711573923  0.6441189536475522   T   T   T
 0.3333301712728249  0.6666698497790970  0.3558959203574888   T   T   T
 0.0000000000000000  0.0000000000000000  0.4586604819119771   F   F   F
 0.6666700244644730  0.3333300054475643  0.5688938202923026   F   F   F
 0.3333256037647096  0.6666744390083723  0.6745066256227360   T   T   T
 0.0000017178209047 -0.0000017286474708  0.3321485583901655   T   T   T
 0.6666700244644730  0.3333300054475643  0.4448858987153201   F   F   F
 0.3333300053932007  0.6666700244160495  0.5551192553174857   F   F   F
-0.0000017324760162  0.0000017214818990  0.6678555524125694   T   T   T
 0.8344812912746143  0.6689714764043194  0.3918839198936202   T   T   T
 0.4999999999667608  0.0000000000000000  0.4999964557784509   F   F   F
 0.1655204055212806  0.3310319018644565  0.6081180950340775   T   T   T
 0.8344838335031078  0.1655161894822078  0.3918837587272648   T   T   T
 0.4999999999641886  0.4999999999616165  0.4999964557784509   F   F   F
 0.1655178663068078  0.8344821567657270  0.6081182563078890   T   T   T
```

```
  0.3310285436193299  0.1655187267596097  0.3918839199902069   T   T   T
  0.9999999999974278  0.4999999999616165  0.4999964557784509   F   F   F
  0.6689681181910612  0.8344796125763536  0.6081180951296223   T   T   T
  0.6666650824657055  0.3333349331097206  0.3702750803232518   T   T   T
  0.3333300053932007  0.6666700244160495  0.4739043418539026   F   F   F
  0.0000012717492092 -0.0000012561122056  0.5847597859248397   T   T   T
  0.6666670950374245  0.3333329117972068  0.6811986325810890   T   T   T
  0.8359877743116144  0.6719744038840213  0.3169037759720457   T   T   T
  0.5142054835970640  0.0284147804285649  0.4177505669132381   T   T   T
  0.1725499928098628  0.3451099992493738  0.5270680935996452   F   F   F
  0.8451605060267267  0.6903166095071172  0.6314267283983201   T   T   T
  0.8359837907927852  0.1640162135111012  0.3169043404923154   T   T   T
  0.5142066468340637  0.4857933727158104  0.4177511763385537   T   T   T
  0.1725499927475340  0.8274499774065163  0.5270680935996452   F   F   F
  0.8451656179320575  0.1548343987429799  0.6314279043483727   T   T   T
  0.3280255739854470  0.1640122176930302  0.3169037762985915   T   T   T
  0.9715852472096647  0.4857945438114027  0.4177505677491418   T   T   T
  0.6548900007813501  0.8274499774065163  0.5270680935996452   F   F   F
  0.3096834215242638  0.1548395203699325  0.6314267267326714   T   T   T
  0.1548417136039510  0.3096878094982689  0.3685722288534029   T   T   T
  0.8274499774624573  0.6548900008657839  0.4729248179572636   F   F   F
  0.4857945276646182  0.9715852154436970  0.5822499621392023   T   T   T
  0.1640129858815274  0.3280271141738724  0.6830985774236051   T   T   T
  0.3333329158213454  0.6666670910695039  0.3187862617916118   T   T   T
 -0.0000012569686982  0.0000012725823388  0.4152419827076551   T   T   T
  0.6666700244644730  0.3333300054475643  0.5261008121564572   F   F   F
  0.3333349331712516  0.6666650824000385  0.6297264783032418   T   T   T
  0.1548365964186038  0.8451634201724434  0.3685710516201358   T   T   T
  0.8274499773475057  0.1725499927682606  0.4729248179572636   F   F   F
  0.4857933526030875  0.5142066669858308  0.5822493533832197   T   T   T
  0.1640170010854404  0.8359830032075286  0.6830980125027302   T   T   T
  0.6903122213569710  0.8451583126685489  0.3685722271980032   T   T   T
  0.3451099992319087  0.1725499927682606  0.4729248179572636   F   F   F
  0.0284148122088111  0.5142054997688701  0.5822499629791867   T   T   T
  0.6719728631248566  0.8359870058395423  0.6830985777448144   T   T   T

CONTCAR_TET1.vasp
   1.00000000000000
     6.0163998604000000    0.0000000000000000    0.0000000000000000
    -3.0081999302000000    5.2103551183999999    0.0000000000000000
     0.0000000000000000    0.0000000000000000   44.5619010925000012
   Fe   O
    29    40
Selective dynamics
Direct
  0.6666650080185221  0.3333360190764550  0.3259794017256215   T   T   T
  0.3333300053932007  0.6666700244160495  0.4310990730427591   F   F   F
  0.0000000000000000  0.0000000000000000  0.5413446903426689   F   F   F
  0.6666682734404766  0.3333320911417957  0.6466788174436791   T   T   T
  0.3333054808376030  0.6666947780425648  0.7287749760359189   T   T   T
  0.6666932911108518  0.3333069666013084  0.2712429268273398   T   T   T
  0.3333320716804110  0.6666682886195332  0.3533220530847817   T   T   T
```

0.0000000000000000	0.0000000000000000	0.4586604819119771	F	F	F
0.6666700244644730	0.3333300054475643	0.5688938202923026	F	F	F
0.3333361701388941	0.6666648740814535	0.6740317944543914	T	T	T
0.0000054263148364	-0.0000043621548518	0.3401982241322801	T	T	T
0.6666700244644730	0.3333300054475643	0.4448858987153201	F	F	F
0.3333300053932007	0.6666700244160495	0.5551192553174857	F	F	F
-0.0000044563173852	0.0000055237402479	0.6598030255275223	T	T	T
0.1669663153498843	0.3339339751300909	0.2866532478799687	T	T	T
0.8332316976886892	0.6664750363421820	0.3922230006496743	T	T	T
0.4999999999667608	0.0000000000000000	0.4999964557784509	F	F	F
0.1667760221231610	0.3335402689665721	0.6077805761762239	T	T	T
0.8330331371197742	0.6660668165307749	0.7133497753348697	T	T	T
0.1669662824251758	0.8330322327449962	0.2866541097030332	T	T	T
0.8332354532972563	0.1667645866552217	0.3922231918242382	T	T	T
0.4999999999641886	0.4999999999616165	0.4999964557784509	F	F	F
0.1667721819389665	0.8332278595152025	0.6077803631650698	T	T	T
0.8330321443932582	0.1669663274800747	0.7133489033318314	T	T	T
0.6660671682552330	0.8330335085531646	0.2866532514984976	T	T	T
0.3335250172187055	0.1667683489513998	0.3922229818537827	T	T	T
0.9999999999974278	0.4999999999616165	0.4999964557784509	F	F	F
0.6664597845659925	0.8332240252572745	0.6077805573041857	T	T	T
0.3339343584609548	0.1669666805205136	0.7133497806839421	T	T	T
-0.0000018084176867	0.0000020846165364	0.2684426616599008	T	T	T
0.6666669375709301	0.3333331860596951	0.3693720209427628	T	T	T
0.3333300053932007	0.6666700244160495	0.4739043418539026	F	F	F
0.0000015865726196	-0.0000014449906471	0.5850408531933199	T	T	T
0.6666663105555962	0.3333341974324642	0.6895282775696902	T	T	T
0.8450065639772183	0.6900107009733714	0.3128695339914465	T	T	T
0.5139645209655274	0.0279362860853080	0.4176184419138351	T	T	T
0.1725499928098628	0.3451099992493738	0.5270680935996452	F	F	F
0.8464843221677201	0.6929599681559369	0.6328389739073657	T	T	T
0.5060135152807496	0.0120153869316101	0.7345852528141663	T	T	T
0.8450104536491387	0.1549903233829617	0.3128705394754058	T	T	T
0.5139675726182231	0.4860323509002275	0.4176188030884834	T	T	T
0.1725499927475340	0.8274499774065163	0.5270680935996452	F	F	F
0.8464848368409565	0.1535154228366230	0.6328397264032511	T	T	T
0.5060650287360015	0.4939347862446644	0.7345863121947225	T	T	T
0.3099914035417348	0.1549948752047562	0.3128693625564747	T	T	T
0.9720638028824122	0.4860354861446070	0.4176184344721574	T	T	T
0.6548900007813501	0.8274499774065163	0.5270680935996452	F	F	F
0.3070401178695954	0.1535158513743616	0.6328390242118278	T	T	T
0.9879795134607016	0.4939838391686902	0.7345854123582118	T	T	T
0.4939880361189867	0.9879877226661150	0.2654205715930331	T	T	T
0.1535129388029403	0.3070341768647786	0.3671613797884221	T	T	T
0.8274499774624573	0.6548900008657839	0.4729248179572636	F	F	F
0.4860318122172791	0.9720564391510980	0.5823828456559241	T	T	T
0.1549853479658789	0.3099722539557694	0.6871332343370017	T	T	T
0.3333341610297562	0.6666663347432911	0.3104738373676152	T	T	T
-0.0000014556646561	0.0000015964392562	0.4149616181056525	T	T	T
0.6666700244644730	0.3333300054475643	0.5261008121564572	F	F	F
0.3333331109918730	0.6666670096803319	0.6306371094265986	T	T	T
0.0000022042048821	-0.0000019207039583	0.7315600177139986	T	T	T

```
  0.4939400438003316  0.5060597598013521  0.2654196934505099   T   T   T
  0.1535124416416976  0.8464878129245677  0.3671606820458632   T   T   T
  0.8274499773475057  0.1725499927682606  0.4729248179572636   F   F   F
  0.4860286278083820  0.5139712953306890  0.5823824815954123   T   T   T
  0.1549809314493599  0.8450198547303133  0.6871320967666609   T   T   T
  0.0120072488928921  0.5060093483513838  0.2654207262276939   T   T   T
  0.6929659062935657  0.8464872307631981  0.3671614298427265   T   T   T
  0.3451099992319087  0.1725499927682606  0.4729248179572636   F   F   F
  0.0279436492135437  0.5139681944054499  0.5823828381039402   T   T   T
  0.6900298767430253  0.8450161086929734  0.6871330607464670   T   T   T

CONTCAR_TET2.vasp
   1.00000000000000
     6.0163998604000000    0.0000000000000000    0.0000000000000000
    -3.0081999302000000    5.2103551183999999    0.0000000000000000
     0.0000000000000000    0.0000000000000000   44.5619010925000012
   Fe   O
    21    24
Selective dynamics
Direct
  0.6666653686963910  0.3333346348147022  0.3262617573260913   T   T   T
  0.3333300053932007  0.6666700244160495  0.4310990730427591   F   F   F
  0.0000000000000000  0.0000000000000000  0.5413446903426689   F   F   F
  0.6666680126716144  0.3333319995502667  0.6473263337387997   T   T   T
  0.3333319990474095  0.6666680131459128  0.3526714925408342   T   T   T
  0.0000000000000000  0.0000000000000000  0.4586604819119771   F   F   F
  0.6666700244644730  0.3333300054475643  0.5688938202923026   F   F   F
  0.3333346343176402  0.6666653692290261  0.6737382793324134   T   T   T
 -0.0000005580881868  0.0000005683633654  0.3462804694706126   T   T   T
  0.6666700244644730  0.3333300054475643  0.4448858987153201   F   F   F
  0.3333300053932007  0.6666700244160495  0.5551192553174857   F   F   F
  0.0000005680926279 -0.0000005578121950  0.6537209868102768   T   T   T
  0.8302120239648216  0.6604298787259563  0.3932927075460523   T   T   T
  0.4999999999667608  0.0000000000000000  0.4999964557784509   F   F   F
  0.1697895729697665  0.3395732957767859  0.6067077617664129   T   T   T
  0.8302140784828828  0.1697859365162989  0.3932923725200145   T   T   T
  0.4999999999641886  0.4999999999616165  0.4999964557784509   F   F   F
  0.1697875159339185  0.8302124991235044  0.6067080971187253   T   T   T
  0.3395701344633974  0.1697879940105002  0.3932927078151290   T   T   T
  0.9999999999974278  0.4999999999616165  0.4999964557784509   F   F   F
  0.6604267174230466  0.8302104450381869  0.6067077620323438   T   T   T
  0.6666653481514851  0.3333346612479511  0.3669011790532698   T   T   T
  0.3333300053932007  0.6666700244160495  0.4739043418539026   F   F   F
  0.0000008826073676 -0.0000008682251030  0.5853267563140395   T   T   T
  0.5123179848110100  0.0246377971217659  0.4168060481894755   T   T   T
  0.1725499928098628  0.3451099992493738  0.5270680935996452   F   F   F
  0.8403891481253821  0.6807750593114840  0.6343119761541791   T   T   T
  0.5123174725865643  0.4876825475559003  0.4168064902239684   T   T   T
  0.1725499927475340  0.8274499774065163  0.5270680935996452   F   F   F
  0.8403890213313843  0.1596109881220797  0.6343118340439585   T   T   T
  0.9753622279655728  0.4876820347566291  0.4168060484204930   T   T   T
  0.6548900007813501  0.8274499774065163  0.5270680935996452   F   F   F
```

0.3192249514219609	0.1596108605942939	0.6343119761227199	T	T	T
0.1596120050190865	0.3192272436415971	0.3656876016405879	T	T	T
0.8274499774624573	0.6548900008657839	0.4729248179572636	F	F	F
0.4876823346932540	0.9753628311501674	0.5831937830410369	T	T	T
-0.0000008686164377	0.0000008829810281	0.4146737779521080	T	T	T
0.6666700244644730	0.3333300054475643	0.5261008121564572	F	F	F
0.3333346609486610	0.6666653484799819	0.6330988251029722	T	T	T
0.1596121343307826	0.8403878750983971	0.3656877439298987	T	T	T
0.8274499773475057	0.1725499927682606	0.4729248179572636	F	F	F
0.4876828447306106	0.5123171754263592	0.5831933405938657	T	T	T
0.6807727670843604	0.8403880036763356	0.3656876016065925	T	T	T
0.3451099992319087	0.1725499927682606	0.4729248179572636	F	F	F
0.0246371939643996	0.5123176848944633	0.5831937832747396	T	T	T

14. Coordinates of 6H_2O-MgO(001)-(3x2) Surface – MLFF MD snapshot and PBE optimized

```
H Mg O
  1.00000000000000
    5.9219999999999997   0.0000000000000000   0.0000000000000000
    0.0000000000000000   8.8829999999999991   0.0000000000000000
    0.0000000000000000   0.0000000000000000  20.0000000000000000
  H   Mg   O
   12   24   30
Selective dynamics
Direct
  0.4679368899954376  0.1811433389803675  0.4353099674447326   T   T   T
  0.2881904988512814  0.5228962946677911  0.3718111281292674   T   T   T
  0.1685468930659181  0.6728972027168065  0.4401256265176421   T   T   T
  0.5212712447718270  0.6374375076889363  0.4566071757974074   T   T   T
  0.9445553415434566  0.7760766185856000  0.4451757952465424   T   T   T
  0.9729207460900551  0.4592233637404471  0.4448097472848066   T   T   T
  0.9494780847512925  0.0986276699222825  0.4473318452869848   T   T   T
  0.1862923059210873  0.0124494319182447  0.4389143721894166   T   T   T
  0.5974928882565074  0.3374126272836324  0.4560850653436980   T   T   T
  0.1711930682479654  0.3415064457791668  0.4461112550254407   T   T   T
  0.5326452196793932  0.9599508626460121  0.4584158045517593   T   T   T
  0.2801056975503292  0.8285083450094178  0.3725757329257590   T   T   T
  0.7491203969157727  0.4987911717205378  0.2129862365628666   T   T   T
  0.0000000000000000  0.6666700399999996  0.1046949999999995   F   F   F
  0.7490560185843141  0.1672167612147101  0.2121828414408727   T   T   T
  0.2500000000000000  0.8333399799999981  0.0000050000000016   F   F   F
  0.2499858789605303  0.8353659597248706  0.2095461702228983   T   T   T
  0.7487446065313208  0.8363008290713129  0.2130999711121806   T   T   T
  0.5000000000000000  0.6666700399999996  0.1046949999999995   F   F   F
  0.7500000000000000  0.8333399799999981  0.0000050000000016   F   F   F
  0.7500000000000000  0.5000000000000000  0.0000050000000016   F   F   F
  0.5202389473732251  0.6688942887688852  0.3216861748488984   T   T   T
  0.0000000000000000  0.3333299600000004  0.1046949999999995   F   F   F
  0.5000000000000000  0.3333299600000004  0.1046949999999995   F   F   F
  0.2500000000000000  0.5000000000000000  0.0000050000000016   F   F   F
  0.9891722300521677  0.0082026833434329  0.3228631335222970   T   T   T
  0.9764155413146353  0.6675009401163543  0.3223727175523646   T   T   T
  0.2496018950740003  0.5000284188444301  0.2097562733634520   T   T   T
  0.5061613136243277  0.0105924586660319  0.3246099548658717   T   T   T
  0.2500000000000000  0.1666700399999996  0.0000050000000016   F   F   F
  0.5000000000000000  0.0000000000000000  0.1046949999999995   F   F   F
  0.2484307179142906  0.1672906072338650  0.2132470648238940   T   T   T
  0.5044723965422612  0.3261081327485893  0.3220162861048660   T   T   T
  0.9901134923272847  0.3278464664948052  0.3230367203324623   T   T   T
  0.7500000000000000  0.1666700399999996  0.0000050000000016   F   F   F
  0.0000000000000000  0.0000000000000000  0.1046949999999995   F   F   F
  0.0000000000000000  0.3333299600000004  0.0000050000000016   F   F   F
  0.2432292524505304  0.5003942556483243  0.3245349663183756   T   T   T
  0.0000000000000000  0.6666700399999996  0.0000050000000016   F   F   F
```

0.7492189601885212	0.1684759733175590	0.3163641251714048	T	T	T
0.0011654829948747	0.9983772448881688	0.2119906391295505	T	T	T
0.7466963577974303	0.8374248107722821	0.3175377602942525	T	T	T
0.5000000000000000	0.6666700399999996	0.0000050000000016	F	F	F
0.2500000000000000	0.8333399799999981	0.1046949999999995	F	F	F
0.7500000000000000	0.8333399799999981	0.1046949999999995	F	F	F
0.4952946678215767	0.6675232942222897	0.2138661539863105	T	T	T
0.0010323512690373	0.3365746411269346	0.2119116016980622	T	T	T
0.7500000000000000	0.5000000000000000	0.1046949999999995	F	F	F
0.7459846068751711	0.4978427984580801	0.3178526304565501	T	T	T
0.2430808465765639	0.8332172288567247	0.3245175811452209	T	T	T
0.4124448759100142	0.6584782686686123	0.4218393934857808	T	T	T
0.0034911794989987	0.6675299150509389	0.2143850749344678	T	T	T
0.7500000000000000	0.1666700399999996	0.1046949999999995	F	F	F
0.4970085664518854	0.3359085198162091	0.2119199781654199	T	T	T
0.2500000000000000	0.5000000000000000	0.1046949999999995	F	F	F
0.5000000000000000	0.3333299600000004	0.0000050000000016	F	F	F
0.0000000000000000	0.0000000000000000	0.0000050000000016	F	F	F
0.2500000000000000	0.1666700399999996	0.1046949999999995	F	F	F
0.5000000000000000	0.0000000000000000	0.0000050000000016	F	F	F
0.2452855014271164	0.1689478626503260	0.3161235345116218	T	T	T
0.4653173715709122	0.3017861117823938	0.4321174306846000	T	T	T
0.4968959210557951	0.9991558946936649	0.2119455978825485	T	T	T
0.9929589735892005	0.6737092822954268	0.4336699681563416	T	T	T
0.0121965577527226	0.0021945912936847	0.4334202981208418	T	T	T
0.0069729407229743	0.3522992689733179	0.4364624883509222	T	T	T
0.4426237267106775	0.0211928084694686	0.4285347713062160	T	T	T

15. Coordinates of $4H_2O$-Fe_3O_4(111)-(1x1) Surface – MLFF MD snapshot and MLFF optimized

```
CONTCAR
   1.00000000000000
     6.0163998604000000    0.0000000000000000    0.0000000000000000
    -3.0081999302000000    5.2103551183999999    0.0000000000000000
     0.0000000000000000    0.0000000000000000   21.0000000000000000
   Fe   O    H
    12    20     8
Selective dynamics
Direct
  0.5455975867312947  0.0897550377605711  0.2030113370160641   T   T   T
  0.2448827802863314  0.4696999130566084  0.4241939547289009   T   T   T
  0.5410299897525661  0.0820500031735421  0.0292368538095218   F   F   F
  0.2162466291016483  0.4335457344765066  0.2596686205700796   T   T   T
  0.2076900005136437  0.4153900146569356  0.0000000000000000   F   F   F
  0.8815409313541145  0.7615916381685143  0.2309784886528520   T   T   T
  0.0410300009220350  0.0820500031735421  0.1169657087619029   F   F   F
  0.7136917688840284  0.4281585621311850  0.3484845479492923   T   T   T
  0.0410300009149154  0.5820500253985159  0.1169657087619029   F   F   F
  0.7147739730221739  0.9531185896411782  0.3471117206228428   T   T   T
  0.5410299897454465  0.5820500253985159  0.1169657087619029   F   F   F
  0.2316877249632326  0.9518133347393366  0.3463610989851655   T   T   T
  0.8743600248008292  0.7487199901641191  0.0615840094285716   F   F   F
  0.5534932183234564  0.1093591510263305  0.2900915015402695   T   T   T
  0.7204247964478836  0.4398487849682127  0.1755259678671265   T   T   T
  0.3887974416216553  0.7856286971355612  0.3932578757442810   T   T   T
  0.7186439251086418  0.9107349520077519  0.1750336807295480   T   T   T
  0.3926257123228685  0.2983278805465981  0.3945272922263319   T   T   T
  0.1931998522068782  0.9109630563642976  0.1747615224432429   T   T   T
  0.8860554735741883  0.2772489414747735  0.3976237571940537   T   T   T
  0.3684700131513381  0.7369400263026833  0.0595165727142870   F   F   F
  0.0445881078277223  0.0886535885043036  0.2864282403214214   T   T   T
  0.2102822671723201  0.4206237671317362  0.1726664848024276   T   T   T
  0.8928904666852865  0.7833122373917834  0.3885247426476239   T   T   T
  0.3684700130669114  0.2546100019776318  0.0595165727142870   F   F   F
  0.0445824974982954  0.6080770538411692  0.2871833845733184   T   T   T
  0.8861399888442421  0.2546100019776318  0.0595165727142870   F   F   F
  0.5628124298492931  0.6099626411362311  0.2871898520659900   T   T   T
  0.5056327273613573  0.5019951345483467  0.5912075867402864   T   T   T
  0.8373792115986480  0.2688945886198429  0.5272193023166073   T   T   T
  0.4739403057266299  0.8199556014286210  0.5557219667647414   T   T   T
  0.0903516091909678  0.9424813846583172  0.4861304763495025   T   T   T
  0.4676370338662422  0.3083115973877925  0.5660097344600177   T   T   T
  0.6879720597036540  0.7477673510580641  0.5786372477608835   T   T   T
  0.7813549012932690  0.2455370813065174  0.5867160583590691   T   T   T
  0.8612609531054934  0.4649706672455836  0.4946128814389620   T   T   T
  0.2710920594056042  0.6336081865245564  0.5684639546256764   T   T   T
  0.5298822472243897 -0.0331093424408530  0.5082898423348264   T   T   T
```

0.9494617493354839 0.8441970631491341 0.4321458044672889 T T T
-0.0011906057036466 0.0641899750044270 0.4920043154099127 T T T

16. Coordinates of $4H_2O$-Fe_3O_4(111)-(1x1) Surface – MLFF MD snapshot and PBE+D2 optimized

```
CONTCAR
  1.00000000000000
    6.0163998604000000    0.0000000000000000    0.0000000000000000
   -3.0081999302000000    5.2103551183999999    0.0000000000000000
    0.0000000000000000    0.0000000000000000   21.0000000000000000
  Fe   O    H
   12   20    8
Selective dynamics
Direct
  0.5388747014393778  0.0769230056889008  0.1977193998186073   T   T   T
  0.1942505457605657  0.3905959158332553  0.4130472132478848   T   T   T
  0.5410299897525661  0.0820500031735421  0.0292368538095218   F   F   F
  0.2011666735155924  0.4022559276276526  0.2562244543658899   T   T   T
  0.2076900005136437  0.4153900146569356  0.0000000000000000   F   F   F
  0.8700768765770110  0.7392781848699304  0.2259670847194056   T   T   T
  0.0410300009220350  0.0820500031735421  0.1169657087619029   F   F   F
  0.6940475383955761  0.3937249014839196  0.3393984037934921   T   T   T
  0.0410300009149154  0.5820500253985159  0.1169657087619029   F   F   F
  0.6935753572013329  0.8942828539421065  0.3386534485225081   T   T   T
  0.5410299897454465  0.5820500253985159  0.1169657087619029   F   F   F
  0.2053070487614612  0.8983175762535888  0.3368605020988616   T   T   T
  0.8743600248008292  0.7487199901641191  0.0615840094285716   F   F   F
  0.5342367300944476  0.0659051636043679  0.2857295240925318   T   T   T
  0.7142322445383611  0.4281813662451454  0.1714867021847420   T   T   T
  0.3622233756278876  0.7232336917317416  0.3845817721226490   T   T   T
  0.7160281104483935  0.9016348783424685  0.1722642756160414   T   T   T
  0.3642528530392346  0.2293968307994594  0.3841620675389488   T   T   T
  0.1857796062991896  0.9019273458045013  0.1722583807954180   T   T   T
  0.8569097517518627  0.2268027891820095  0.3858753382005212   T   T   T
  0.3684700131513381  0.7369400263026833  0.0595165727142870   F   F   F
  0.0273605223288334  0.0549177188075372  0.2763840903780211   T   T   T
  0.2058540779030063  0.4124321770891627  0.1690463994238915   T   T   T
  0.8715396526141195  0.7324375803076498  0.3805929715297738   T   T   T
  0.3684700130669114  0.2546100019776318  0.0595165727142870   F   F   F
  0.0263174105009221  0.5761854018429925  0.2757758617655481   T   T   T
  0.8861399888442421  0.2546100019776318  0.0595165727142870   F   F   F
  0.5512944318022136  0.5757734594422297  0.2760973458923320   T   T   T
  0.6618605974519441  0.4744288751947601  0.5638671130572350   T   T   T
  0.0565851527902718  0.8079711563116303  0.4982117122665035   T   T   T
  0.5869897323949654  0.0486463150835817  0.5030097573150423   T   T   T
  0.1291459259438953  0.2636552013128871  0.5060667703498591   T   T   T
  0.6190269359182171  0.3093035471451472  0.5423087681077495   T   T   T
  0.5079492626205667  0.4881918324570769  0.5590896747276241   T   T   T
  0.9077665428179612  0.6870211819177074  0.5268961366975735   T   T   T
```

0.9828243573423021	0.7679949275639237	0.4537484433936088	T	T	T
0.3982822415067961	0.9311138448921542	0.5028585637974087	T	T	T
0.6345003474261852	0.0869859486928517	0.4576915701586476	T	T	T
0.1081215509003183	0.0792195590438542	0.5054567257857409	T	T	T
-0.0416935576735394	0.2272893123753920	0.5205415074867595	T	T	T
