# Supplementary material for: Machine Learning in Computational Surface Science and Catalysis: Case Studies on Water and Metal–Oxide Interfaces
Source: Front Chem. 2020 Nov 30;8:601029. doi: 10.3389/fchem.2020.601029 (PMC7793815; doi:10.3389/fchem.2020.601029)
Supplement: Supplementary file 3 [file Presentation_1.PPTX]

## Slide 1
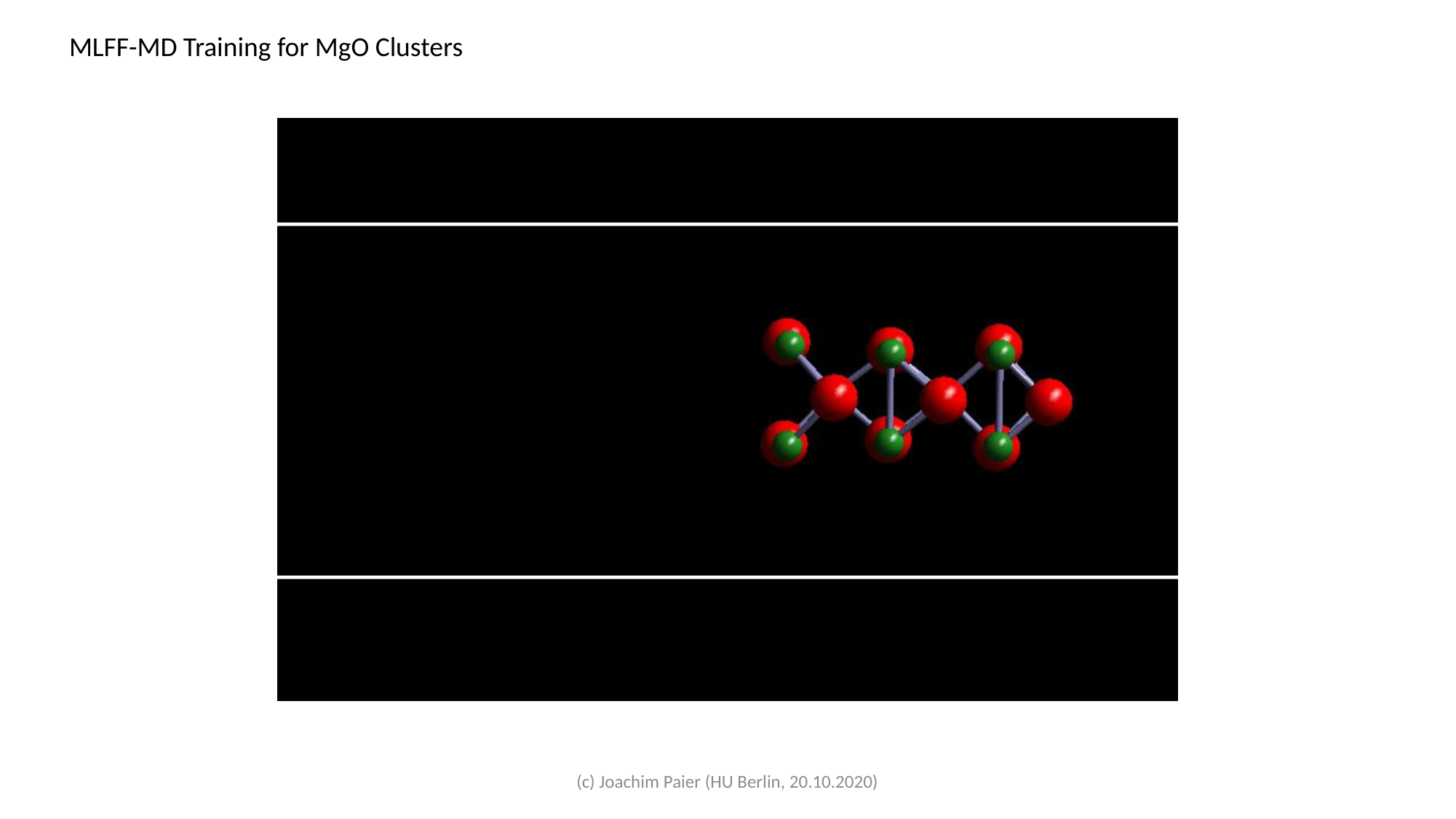

MLFF-MD Training for MgO Clusters
(c) Joachim Paier (HU Berlin, 20.10.2020)

## Slide 2
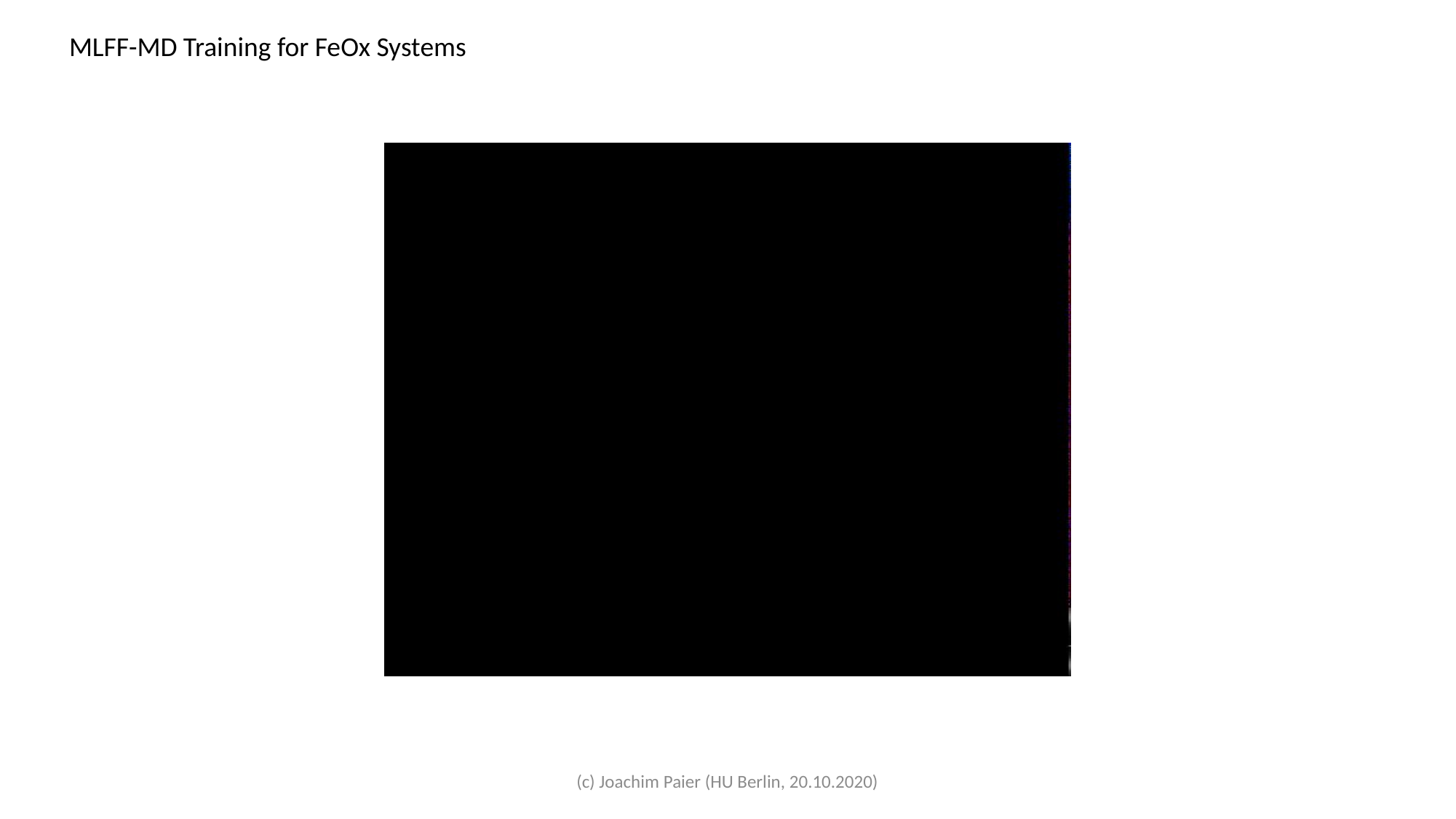

MLFF-MD Training for FeOx Systems
(c) Joachim Paier (HU Berlin, 20.10.2020)
